# A feline coronavirus nucleocapsid protein disrupts ZC3HAV1–viral RNA association to counteract host RNA-level restriction

**DOI:** 10.1371/journal.ppat.1014616

**Published:** 2026-09-18

**Authors:** Miao Zhang, Na Li, Kelimujiang Aishanjiang, Aoxing Tang, Meng Zhu, Shiqiang Zhu, Da Zhang, Lingxue Yu, Jie Zhu, Chuanfeng Li, Chunchun Meng, Yingqi Zhu, Guoxin Li, Guangqing Liu

**Affiliations:** Shanghai Institute of Infectious Disease and Biosecurity, Shanghai Veterinary Research Institute, Chinese Academy of Agricultural Sciences (CAAS), Shanghai, China; Tsinghua University, CHINA

## Abstract

**Background:**

Coronaviruses have evolved intricate mechanisms to evade host RNA surveillance systems and sustain efficient replication. The feline infectious peritonitis virus (FIPV), a highly lethal feline coronavirus that causes systemic vasculitis and fatal peritonitis, represents a major challenge in veterinary medicine and serves as a valuable model for studying conserved coronavirus–host interactions. Among coronavirus structural proteins, the nucleocapsid (N) protein not only packages the viral genome but also modulates host antiviral responses, yet its role in counteracting RNA restriction remains incompletely understood.

**Results:**

Using co-immunoprecipitation coupled with mass spectrometry screening, we identified the host RNA-binding antiviral factor ZC3HAV1 (also known as ZAP or PARP13) as a prominent N-associated host protein. ZC3HAV1 restricted FIPV replication by associating with viral RNA and reducing viral RNA accumulation and persistence. We show that the FIPV N protein associates with the RBD-containing region of ZC3HAV1 and that the ZC3HAV1-associated region of N contributes to efficient viral counter-restriction activity. Through coordinated protein–protein and protein–RNA interactions, FIPV N interferes with ZC3HAV1–viral RNA association, thereby supporting viral RNA accumulation and replication. Expression of FIPV N in a heterologous Japanese encephalitis virus system also alleviated ZC3HAV1-mediated RNA-level restriction, indicating that this antagonism activity is functionally transferable and not entirely dependent on FIPV-specific replication machinery. Despite the low CpG content of FIPV RNA, ZC3HAV1 efficiently associated with multiple viral transcripts, suggesting that ZC3HAV1–FIPV RNA recognition is not fully explained by overall CpG abundance and may also be influenced by subgenomic RNA context or structural features.

**Conclusions:**

These findings reveal a previously unrecognized mechanism by which a highly pathogenic feline coronavirus circumvents host RNA restriction. By engaging the RBD-containing region of ZC3HAV1 and disrupting ZC3HAV1–viral RNA association, the FIPV N protein effectively attenuates ZC3HAV1-associated antiviral restriction and helps preserve viral RNA accumulation and persistence. This work expands the functional understanding of coronavirus N proteins beyond genome packaging and immune modulation, and provides new insight into FIPV–host interactions that may inform future studies of RNA-level counter-restriction mechanisms in other animal and human coronaviruses.

## Author summary

Feline infectious peritonitis virus (FIPV) is a deadly coronavirus that causes severe disease in cats and is difficult to treat. One reason FIPV is so challenging to control is its ability to escape the host’s immune response. In this study, we found that FIPV uses its nucleocapsid (N) protein to interfere with a host antiviral protein called ZC3HAV1, which normally helps defend cells by binding to viral RNA and limiting viral RNA accumulation and persistence. We found that ZC3HAV1 can restrict FIPV replication even though FIPV has a low-CpG genome, a feature that might be expected to reduce recognition by a CpG-biased RNA-binding antiviral factor. Instead, ZC3HAV1 association with FIPV RNA appears to be influenced by viral RNA context, including the abundance of certain viral transcripts. Importantly, the FIPV N protein counteracts this restriction by interacting with ZC3HAV1 and disrupting ZC3HAV1–viral RNA association through coordinated protein–protein and protein–RNA interactions. This hijacking of a host defense protein represents a previously unrecognized immune evasion strategy used by FIPV. Our findings provide new insights into how coronaviruses evade RNA-level antiviral restriction and may help inform future studies aimed at identifying antiviral targets.

## Introduction

Feline infectious peritonitis (FIP) is one of the most devastating viral diseases in cats, caused by the feline infectious peritonitis virus (FIPV), a highly virulent biotype of feline coronavirus [1]. Despite decades of research, FIP remains almost uniformly fatal, with limited effective treatments available, posing a significant burden on feline health and veterinary care. Clinically, FIP manifests as pyogranulomatous inflammation, effusive peritonitis, and multiorgan failure, reflecting widespread endothelial damage and immune dysregulation [2,3]. Given its high lethality and systemic nature, FIPV provides not only an urgent clinical target but also an excellent model for dissecting coronavirus–host interactions, particularly those that determine viral RNA accumulation and host restriction evasion.

Coronaviruses have evolved intricate molecular strategies to manipulate host cells and ensure efficient replication. A critical frontier in this virus–host arms race lies in the intrinsic cellular RNA restriction system, which detects and degrades foreign RNA transcripts to prevent infection [4,5]. However, the full spectrum of viral countermeasures against this defense remains incompletely defined. Understanding how pathogenic coronaviruses, such as FIPV, subvert these RNA surveillance pathways is therefore important for explaining how they maintain viral RNA accumulation, efficient replication, and infection-associated pathogenesis.

Among coronavirus structural proteins, the nucleocapsid (N) protein has emerged as a multifunctional regulator that facilitates viral RNA synthesis by associating with both viral RNA and components of the replication–transcription complex (RTC) [6]. Through its high-affinity RNA-binding activity and interactions with nonstructural proteins such as nsp3 and nsp12, N stabilizes viral RNA templates, promotes RTC assembly, and enhances the efficiency of genome replication and subgenomic transcription [7–9]. Beyond this canonical role, the N protein has been increasingly implicated in modulating host RNA metabolism [10,11] and antiviral signaling [12,13], suggesting that it may also influence the susceptibility of viral RNAs to host RNA surveillance pathways. However, whether this putative RNA-protective function involves counteraction of host cytoplasmic RNA-level restriction, a crucial antiviral pathway that acts as an early intracellular barrier to viral replication [14], remains poorly understood. Elucidating this link would provide key insight into how coronavirus N proteins integrate viral RNA synthesis with the evasion of host RNA restriction systems.

To address this question, we performed an unbiased co-immunoprecipitation coupled with mass spectrometry (Co-IP/MS) screen in FIPV-infected cells to identify host proteins that associate with N. The analysis revealed extensive interactions with host RNA-binding factors, among which the restriction factor ZC3HAV1 (also known as ZAP or PARP13) emerged as a prominent candidate. ZC3HAV1 is an interferon-stimulated gene product that binds viral RNA and, in several viral systems, suppresses replication by recruiting RNA-surveillance and decay-associated factors [15,16]. Accumulating evidence has established ZC3HAV1 as an antiviral restriction factor against diverse viruses, including several positive-strand RNA viruses and coronaviruses. In coronaviruses, ZC3HAV1-mediated restriction has been reported for SARS-CoV-2 and porcine epidemic diarrhea virus (PEDV) [17,18], indicating that coronavirus RNAs can be susceptible to ZC3HAV1-associated RNA-level restriction despite the generally CpG-suppressed nature of many coronavirus genomes. However, the basis by which low-CpG coronavirus RNAs remain susceptible to ZC3HAV1-associated restriction remains incompletely understood, particularly with regard to whether residual CpG distribution, other RNA-context features, or virus-specific RNA-binding proteins influence this process. Moreover, previous coronavirus studies have mainly focused on the antiviral activity of ZC3HAV1 itself, whereas how coronaviruses counteract ZC3HAV1-mediated RNA restriction remains much less understood. In particular, whether coronavirus structural proteins can interfere with the ZC3HAV1–viral RNA axis has not been fully defined. Notably, FIPV possesses a low-CpG genome, yet ZC3HAV1 is classically defined as a CpG-biased RNA-binding antiviral factor [17]. This apparent paradox makes FIPV a useful system to investigate how a CpG-depleted coronavirus RNA remains sensitive to ZC3HAV1-associated RNA-level restriction and whether the coronavirus N protein preserves viral RNA accumulation and persistence by antagonizing the ZC3HAV1–viral RNA axis.

Our preliminary data suggested that ZC3HAV1 interacts with the FIPV N protein and acts as a negative regulator of viral replication. We hypothesized that FIPV N antagonizes ZC3HAV1-associated RNA-level restriction by reducing ZC3HAV1 association with viral RNA, thereby preserving viral RNA accumulation and persistence. By focusing on a clinically fatal feline coronavirus, this study not only investigates an RNA-level counter-restriction mechanism but also offers broader insight into how coronaviruses preserve transcript stability to sustain infection. We identify the ZC3HAV1 RBD-containing region as a key interface involved in FIPV N association, demonstrate that FIPV N impairs ZC3HAV1–viral RNA association, and show that ZC3HAV1 recognition of FIPV RNA is not fully explained by overall CpG abundance. Collectively, these findings bridge veterinary and molecular virology by revealing how coronavirus structural proteins subvert intrinsic RNA restriction, thereby expanding our understanding of coronavirus–host interplay at the post-transcriptional level.

## Materials and methods

### Virus, vectors, cells, and animals

SeV, FIPV strain SH2021 (GenBank accession number OR295209.1), and Japanese encephalitis virus (JEV) were provided by the Companion Animal Diseases Team, Shanghai Veterinary Research Institute (SHVRI). Plasmids pCold-SUMO-FIPV-N, pET-28a, pLKO.1, psPAX2, pMD2.G, pCAGGS, pCMV14-Flag, pCMV14-hemmagultinin (HA), pCMV14-Myc, pCMV14-green fluorescent protein (GFP), pGAS-Luc-IFN-β (human), pGL3-IFN-β (feline), and pRL-TK were also provided by SHVRI. CRFK and HEK293T cells were purchased from the Chinese Academy of Sciences Cell Bank. BHK-21 cells were provided by the Companion Animal Diseases Team at SHVRI.

### Plasmid construction

Plasmids were constructed by homologous recombination using the One Step Cloning Kit (Vazyme Biotech Co., Ltd., Nanjing, China). The N gene was amplified from the pCold-SUMO-FIPV-N plasmid, and host genes from cDNA synthesized from CRFK cell RNA. PCR was performed with 2 × Phanta Max Master Mix (Vazyme) following the manufacturer’s instructions. Plasmids were linearized by double digestion, and inserts were assembled by homologous recombination. Prokaryotic expression plasmids were transformed into *Escherichia coli* Rosetta (DE3), and eukaryotic plasmids into *E. coli* DH5α. Primer sequences are listed in S1 Table.

### Western blot (WB) analysis

Cells were seeded and transfected with plasmids using Lipofectamine 3000 (Thermo Fisher Scientific, Waltham, MA, USA) according to the manufacturer’s instructions when they reached 80–90% confluency at 16–24 h after plating. After 12 h, the medium was replaced with maintenance medium (except for 293T cells). Cells or virus-infected samples were collected at the indicated time points and lysed in RIPA buffer on ice for 30 min, followed by centrifugation at 12,000 rpm for 5 min at 4°C. Supernatants were mixed with 4 × SDS loading buffer, boiled for 10 min, and stored at −40°C. Proteins were separated by SDS-PAGE, transferred to 0.22 μm PVDF membranes (Merck Millipore, Burlington, MA, USA), blocked with 5% skim milk, and incubated with primary antibodies (as recommended by the manufacturer) overnight at 4°C or for 1 h at 25°C. After incubation with secondary antibodies, signals were detected using ECL substrate and visualized with an imaging system (Cat#4600; Biotanon, Shanghai, China).

### Determination of 50% tissue culture infectious dose (TCID_50_)

To determine the TCID_50_ of FIPV, whole-cell lysates were collected and subjected to three freeze-thaw cycles, followed by centrifugation at 3,000 × *g* for 10 min at 4°C. The supernatants were collected and serially diluted 10-fold (10^-1^ to 10^-8^) in Eagle’s minimum essential medium. CRFK cells seeded in 96-well plates were washed to remove the culture medium. One hundred microliters of each virus dilution was added to each well using eight replicates per dilution. Negative control wells were included without virus inoculation. Plates were incubated at 37°C in a 5% CO_2_ atmosphere for 5 days. The cytopathic effect (CPE) was recorded, and the TCID_50_ value was calculated using the Reed–Muench method.

### RT-qPCR

To investigate host transcriptional responses to FIPV infection, CRFK cells were infected with FIPV (multiplicity of infection [MOI] = 0.01) or mock-treated. Total RNA was extracted at 12 and 24 h post-infection (hpi) using TRIzol reagent (Accurate Biotechnology Co., Ltd., Shanghai, China), reverse transcribed into cDNA with Evo M-MLV (Accurate Biotechnology Co., Ltd.), and subjected to qPCR using SYBR Green Pro Taq HS Mix (Accurate Biotechnology Co., Ltd.) and gene-specific primers (S2 Table). Relative mRNA levels were normalized to glyceraldehyde 3-phosphate dehydrogenase (GAPDH) and calculated using the 2^−ΔΔCt^ method.

For absolute quantification, FIPV RNA was quantified using primer sets targeting either the N gene or the untranslated region (UTR). For N gene-based quantification, the primers were forward 5′-TAAGGAACTCGCTGAGAGGTGG-3′ and reverse 5′-CTGAAACTGTGGCGGTATCTTA-3′. A standard curve ranging from 10² to 10⁸ copies/μL was generated (y = −3.3351x + 37.367, R² = 0.9958). For UTR-based quantification, the primers were forward 5′-TCCGAGGAATTACTGGTCATC-3′ and reverse 5′-CACTAGATCCAGACGTTAGCTC-3′. A standard curve ranging from 10² to 10⁸ copies/μL was generated (y = −3.3141x + 36.214, R² = 0.9947). Viral RNA loads were calculated from the corresponding standard curves and expressed as copies per μg of total RNA.

### Immunoprecipitation (IP) and co-immunoprecipitation (Co-IP)

IP was performed using either anti-N monoclonal antibodies or tag-based magnetic beads. For direct IP, CRFK cells infected with FIPV (MOI = 0.01) were lysed 16 hpi, and cell lysates were incubated with anti-FIPV N monoclonal antibody (clone 10E7), followed by Protein A/G agarose beads (Cat# 36403ES25; Yeasen Biotech Co., Ltd., Shanghai, China), according to the manufacturer’s instructions. Eluted proteins were analyzed by WB and MS.

For Co-IP, CRFK or 293T cells were transfected with tagged protein constructs using Lipofectamine 3000. Where indicated, CRFK cells were infected with FIPV after transfection. At 24 h post-transfection, cells were lysed, and tagged proteins were immunoprecipitated using anti-tag magnetic beads (ShareBio, Shanghai, China) following the manufacturer’s instructions. After washing, bound proteins were eluted for SDS-PAGE, followed by WB or MS.

### Liquid chromatography (LC)-MS

The target gel bands were excised and subjected to in-gel tryptic digestion. Peptides were analyzed by LC-tandem MS (LC-MS/MS) at Shanghai APT Biotechnology Co., Ltd. (Shanghai, China) for protein identification.

### Screening of host proteins

To identify host proteins interacting with FIPV N protein, IP MS data of N protein complexes were analyzed. Highly abundant host proteins were selected for further study. Overexpression plasmids for the host proteins were constructed and transfected into CRFK cells. After culturing CRFK cells to 80%–90% confluence, plasmids expressing different host proteins or an empty pCAGGS control plasmid were transfected using Lipofectamine 3000. Following a 12-h transfection, the culture medium was replaced with maintenance medium. After 24 h, the cells were washed and infected with FIPV at an MOI of 0.01. After 16 h of infection, cells underwent freeze-thaw cycles, and the supernatant was collected by centrifugation. FIPV titers in the supernatant were determined using the TCID_50_ method.

To analyze the effect of FIPV infection on host gene expression, qPCR was performed to measure transcriptional changes in host genes during the infection process. CRFK cells were seeded in 6-well plates and infected with FIPV at an MOI of 0.01, with uninfected cells serving as controls. Samples were collected at 12 and 24 h post-infection (hpi). Total RNA was extracted using TRIZOL, and cDNA was synthesized using the Evo M-MLV reverse transcription kit. qPCR was then conducted using the SYBR Green Pro Taq HS qPCR kit to assess the transcriptional levels of host genes potentially involved in FIPV replication.

### Effect of ZC3HAV1 overexpression on FIPV replication

To investigate the role of ZC3HAV1 in FIPV replication, CRFK cells were transfected with pCAGGS-ZC3HAV1 or control plasmid pCAGGS-Flag. At 12 h post-transfection, the medium was replaced, and cells were subsequently infected with FIPV (MOI = 0.01). At the indicated time points post-infection, cells were harvested for analysis of FIPV N protein by Western blotting, viral RNA levels by absolute RT-qPCR, and viral titers by TCID50 assay. In addition, dose-dependent effects of ZC3HAV1 were assessed by transfecting increasing amounts of ZC3HAV1 expression plasmid, followed by analysis of FIPV N protein at 16 h post-infection.

To evaluate whether FIPV infection affects endogenous ZC3HAV1 expression, CRFK cells were infected with FIPV (MOI = 0.01) and collected at the indicated time points for analysis by RT-qPCR and Western blotting. Furthermore, to assess whether FIPV N protein directly regulates ZC3HAV1 expression, CRFK cells were co-transfected with Flag-ZC3HAV1 and increasing amounts of HA-tagged N plasmids, and ZC3HAV1 protein levels were determined by Western blotting.

### Effect of knockdown of endogenous ZC3HAV1 on FIPV replication

Three small interfering RNA (siRNA) sequences targeting ZC3HAV1 were designed and synthesized. Their sequences are listed in S3 Table. CRFK cells were transfected with each siRNA or a non-targeting control (siNC), and knockdown efficiency was evaluated by qPCR at 48 h post-transfection and by WB at 60 h to identify the most effective siRNA. To assess its effect on FIPV replication, CRFK cells were transfected with the optimal siRNA or siNC, and infected with FIPV (MOI = 0.01) 48 h later. Samples were collected at 12, 16, and 24 hpi for WB analysis of N protein and endogenous ZC3HAV1 expression. Viral RNA levels and titers were quantified by qPCR and TCID_50_ assay, respectively.

### Dual-luciferase reporter gene assay

Effect of ZC3HAV1 and FIPV N on IFN-β transcription: 293T cells were seeded in 24-well plates and co-transfected with pGAS-Luc-IFN-β (60 ng), pRL-TK (10 ng), and either pCAGGS-Flag (400 ng), pCAGGS-FIPV N (400 ng), or pCAGGS-ZC3HAV1 (400 ng). After 18 h, cells were stimulated with SeV for 12 h, and luciferase activity was measured using the Dual-Luciferase Reporter Gene Assay Kit (Easen Biotechnology Co., Ltd., Shanghai, China) according to the manufacturer’s instructions.

Antagonistic effect of ZC3HAV1 on FIPV N-mediated IFN-β regulation: 293T cells were seeded in 24-well plates and transfected at 80–90% confluency with pGAS-Luc-IFN-β (human, 60 ng), pRL-TK (10 ng), and one of the following plasmid combinations: pCAGGS-Flag (400 ng); pCAGGS-FIPV N (200 ng, 400 ng, or 800 ng); pCAGGS-ZC3HAV1 (200 ng) + pCAGGS-Flag (200 ng); or pCAGGS-FIPV N (200 ng) + pCAGGS-ZC3HAV1 (200 ng). Each condition was performed in triplicate. After 16 h of transfection, cells were stimulated with SeV for an additional 12 h. Luciferase activity was measured using the Dual-Luciferase Reporter Gene Assay Kit according to the manufacturer’s instructions. For CRFK cells, the same procedure was applied, except that pGAS-Luc-IFN-β (human) was replaced with pGL3-IFN-β (feline) for species-specific validation.

### IP and Co-IP verification of the interaction between ZC3HAV1 and FIPV N

The interaction between ZC3HAV1 and the FIPV N protein was validated through IP and Co-IP assays. To exclude the possibility that RNA mediates the interaction, RNase was added to the cell lysate (final concentration: 50 µg/mL) during lysis in the Co-IP experiments.

### Confocal microscopy analysis

To examine the subcellular localization and colocalization of ZC3HAV1 and FIPV N, CRFK cells were seeded on glass coverslips and transfected with the following combinations of expression plasmids: pCMV14-Myc-FIPV N + pCMV14-HA (control), pCMV14-HA-ZC3HAV1 + pCMV14-Myc (control), or pCMV14-Myc-FIPV N + pCMV14-HA-ZC3HAV1 (experimental group). At 24 h post-transfection, cells were fixed with 4% paraformaldehyde, permeabilized with 2% Triton X-100, and stained using anti-hemagglutinin (HA) and anti-Myc antibodies followed by AF594- and AF488-conjugated secondary antibodies. Nuclei were counterstained with DAPI. After mounting, cells were visualized by confocal laser scanning microscopy.

In parallel, to confirm the subcellular localization of endogenous proteins under physiological infection conditions, CRFK cells infected with FIPV (MOI = 0.01) were fixed with 4% paraformaldehyde and permeabilized with 2% Triton X-100. Mock-infected cells were included as negative controls. After blocking with 5% BSA, cells were incubated with primary antibodies against endogenous ZC3HAV1 (mouse polyclonal antibody, kindly provided by the Companion Animal Research Group, Shanghai Veterinary Research Institute) and FIPV N protein overnight at 4°C, followed by incubation with Alexa Fluor–conjugated secondary antibodies. Nuclei were stained with DAPI, and images were acquired using a laser-scanning confocal microscope.

### Glutathione S-transferase (GST) pull-down assay

To determine whether the interaction between ZC3HAV1 and FIPV N is direct, an in vitro GST pull-down assay was performed. Recombinant ZC3HAV1 and FIPV N proteins were expressed in *E. coli* Rosetta (DE3) cells using the pET-28a-ZC3HAV1, pEGX-4T-1-FIPV N, and empty pEGX-4T-1 plasmids. To enhance the yield of ZC3HAV1 in the supernatant during prokaryotic expression, cultures were induced with 1 mM IPTG at 16°C for 16 h. Bacterial pellets were harvested by sonication, and the supernatants were collected for SDS-PAGE and WB analysis. For the pull-down assay, GST magnetic beads (Cat#C650031; BBI Life Sciences, Shanghai, China) were incubated with bacterial lysates expressing GST or GST-FIPV N for 1 h, washed twice, and then incubated with His-ZC3HAV1 lysates for an additional 1 h at 25°C. After five washes, the bound proteins were eluted by boiling in SDS loading buffer and analyzed by WB.

### Identification of interaction domains between FIPV N protein and ZC3HAV1

Based on the evidence of direct interaction, further experiments were performed to map the binding regions between ZC3HAV1 and FIPV N. In one set of experiments, HEK293T cells were co-transfected with pCMV14-Myc-FIPV N and a series of ZC3HAV1 truncation mutants (Z1–Z6). In a parallel set, cells were co-transfected with pCMV14-HA-ZC3HAV1 and a panel of FIPV N truncation constructs (N1–N5). All constructs were cloned into pCAGGS. GFP-expressing cells (pCAGGS-GFP) were included as negative controls in both assays. At 24 h post-transfection, cell lysates were subjected to immunoprecipitation using anti-Myc or anti-HA conjugated magnetic beads, followed by Western blot analysis.

In addition, protein–protein interaction models between ZC3HAV1 and FIPV N were predicted using the AlphaFold3 online server (https://alphafoldserver.com/). Model confidence was assessed using predicted Local Distance Difference Test (pLDDT) scores. The resulting models were further visualized using PyMOL software to assess the spatial localization of binding domains.

### Generation of stable ZC3HAV1-knockdown cells and rescue experiments

CRFK cells with stable ZC3HAV1 knockdown were generated using a lentivirus-mediated shRNA approach. Briefly, pLKO.1-ZC3HAV1 was co-transfected with psPAX2 and pMD2.G into HEK293T cells to produce lentiviral particles. Supernatants containing lentiviral particles were collected and used to transduce CRFK cells. Transduced cells were selected with puromycin (6 μg/mL) (Cat# ST551; Biotanon, Shanghai, China) and maintained under puromycin selection for ten consecutive passages to generate stable knockdown cell lines. shRNA sequences are listed in Supplementary S1 Table. Knockdown efficiency was confirmed by Western blotting and RT-qPCR.

For rescue experiments, CRFK-shZC3HAV1 cells were transfected with plasmids expressing wild-type ZC3HAV1 or the ZC3HAV1 truncation mutant Z5, which lacks the RBD-containing region involved in FIPV N association, with or without co-expression of FIPV N protein. At 16 h post-transfection, cells were infected with FIPV (MOI = 0.01). Samples were collected at the indicated time points, and viral replication was evaluated by Western blotting, RT-qPCR, and TCID_50_ assays.

To further evaluate whether the ZC3HAV1-associated region of FIPV N contributes to counteracting ZC3HAV1-mediated antiviral activity, CRFK-shZC3HAV1 cells were transfected with wild-type ZC3HAV1 together with full-length FIPV N or the indicated N-protein truncation mutants (N1–N5). Cells transfected with the corresponding empty vectors served as controls. At 16 h post-transfection, cells were infected with FIPV (MOI = 0.01). Samples were collected at the indicated time points. Protein expression was analyzed by Western blotting, while viral RNA levels and infectious virus titers were determined by RT-qPCR using FIPV-specific primers and TCID_50_ assays, respectively.

### Construction and validation of an RNA-binding-deficient FIPV N mutant

To generate an RNA-binding-deficient mutant of the FIPV N protein, three conserved residues (Y93, R117, and K123) within the N-terminal RNA-binding domain were substituted with alanine using overlap PCR with specifically designed mutagenic primers. The resulting mutant (Mut-N; Y93A/R117A/K123A) was cloned into the pCMV-Myc expression vector and confirmed by DNA sequencing. Primer sequences are listed in Supplementary S1 Table.

To validate RNA-binding activity, wild-type N and Mut-N proteins were expressed in HEK293T cells. The interaction between Mut-N and ZC3HAV1 was first examined by Co-IP assays to determine whether the introduced mutations affected protein–protein interaction. RNA-binding capacity was then assessed by RNA immunoprecipitation (RIP) assays as described below. Viral RNA enrichment was quantified by RT-qPCR and normalized to input RNA.

### Establishment of qPCR detection methods for FIPV gRNA and sgRNA

To investigate whether ZC3HAV1 regulates FIPV genomic RNA (gRNA) replication and affects sgRNA transcription, qPCR assays were developed based on the coronavirus discontinuous transcription mechanism and the shared 5¢ untranslated region (UTR) leader sequence in gRNA and all sgRNAs. The 5¢ UTR leader sequence of FIPV was first identified by PCR using multiple forward primers spanning the entire 5¢ UTR (primer sequences are listed in S4 Table) and a reverse primer targeting the 5¢ proximal region of the N gene open reading frame (ORF), with cDNA synthesized from FIPV-infected CRFK total RNA as the template. Amplification indicated that the upstream sequences were part of the leader region.

Once the leader sequence was confirmed, specific sgRNA detection primers were designed with the forward primer located within the 5¢ UTR leader sequence and the reverse primer targeting the 5¢ region of the desired ORF, ensuring amplification of only the sgRNA of interest. For gRNA-specific detection, primers were designed within the 3¢ region of the pp1a/pp1b ORF (forward) and the 5¢ region of the S gene ORF (reverse), avoiding amplification of sgRNAs. Detailed primer sequences are provided in S4 Table.

### Identification of sgRNA transcription levels during FIPV infection

The synthesis of sgRNA during coronavirus replication follows a temporal order, with sgRNAs from different genes potentially exhibiting distinct expression patterns at various stages of viral replication, including early, middle, and late phases. In subsequent analyses of the impact of ZC3HAV1 on the relative expression of FIPV sgRNA to gRNA, the differences in sgRNA expression at various time points during FIPV replication may have an impact on the results. To explore the transcriptional differences of FIPV sgRNAs, we further investigated sgRNA expression at 12 and 16 hpi in CRFK cells. CRFK cells in good growth condition were seeded in 12-well plates. Upon reaching 100% confluence, FIPV suspension (MOI = 0.01) was added, with an uninfected control group included. Samples were collected at 12 and 16 hpi, and RNA was extracted and reverse-transcribed. qPCR was used to detect FIPV gRNA, and sgRNA was used to assess expression levels.

### Quantitative assessment of ZC3HAV1 effects on FIPV subgenomic RNA expression

To investigate whether ZC3HAV1 regulates the transcription of FIPV sgRNAs, CRFK cells were infected with FIPV (MOI = 0.01). Total RNA was collected at 12 and 16 hpi. The expression levels of sgRNAs were analyzed using a qPCR method established to distinguish between gRNA and sgRNAs. To determine the specific regulatory effect of ZC3HAV1, overexpression and knockdown experiments were performed using pCAGGS-ZC3HAV1 and siRNAs, respectively. sgRNA transcription levels were quantified following normalization to both GAPDH and viral gRNA. Specifically, ΔCt values were calculated relative to GAPDH. The sgRNA levels were normalized to gRNA using the ΔΔCt method: ΔΔCt_sgRNA/gRNA_ = (ΔCt_sgRNA_ − ΔCt_gRNA_)_treatment_−(ΔCt_sgRNA_ − ΔCt_gRNA_)_control._ The relative expression of sgRNA was calculated as 2^−ΔΔCt sgRNA/gRNA^, allowing assessment of ZC3HAV1-mediated regulation independent of overall viral RNA replication.

### GS-441524-based assay for FIPV RNA persistence

To determine whether ZC3HAV1 regulates the abundance of FIPV RNAs independently of ongoing viral RNA synthesis, the RNA-dependent RNA polymerase inhibitor GS-441524 was used to block de novo viral RNA synthesis. CRFK cells were infected with FIPV (MOI = 0.01), and GS-441524 was added at a final concentration of 200 μM at 4 hpi. An untreated infected group served as a positive control. Cells were harvested at 0, 4, 8, and 12 h post-treatment, and total RNA was extracted for RT-qPCR-based quantification of viral genomic RNA (gRNA) and subgenomic RNAs (sgRNAs), which was used to determine the optimal time window for subsequent experiments.To investigate the role of ZC3HAV1, both gain- and loss-of-function approaches were performed in parallel. For overexpression, CRFK cells were transfected with pCMV14-HA-ZC3HAV1 or empty vector 12 h prior to infection. For knockdown, cells were transfected with siZC3HAV1 or negative control siRNA (siNC) 60 h prior to infection. Cells were then infected with FIPV (MOI = 0.01), and GS-441524 (200 μM) was added at 4 hpi to inhibit de novo viral RNA synthesis. Cells were harvested 10 h after drug addition for total RNA extraction and RT-qPCR-based quantification of viral gRNA and sgRNAs.

### Analysis of CpG distribution in FIPV and comparative genomic analysis with other coronaviruses

To investigate whether ZC3HAV1 preferentially targets FIPV RNA via CpG recognition, we analyzed CpG distribution in the FIPV genome and performed a comparative analysis with other coronaviruses. Full-length genomic sequences of FIPV and five representative coronaviruses—HCoV-229E (GenBank: KU291448.1), CCoV (KP981644.1), SARS-CoV-2 (MZ054889.1), IBV (OR824987.1), and PDCoV (MN942260.2)—were retrieved from GenBank. Using R, CpG frequency was calculated in 100 bp non-overlapping windows across genomes, and visualized to assess interspecies differences. For FIPV, CpG observed/expected (O/E) ratios were further calculated in defined genomic regions (e.g., 5′ UTR, ORF1a/1ab, S, N) using sliding window analysis. Regional distributions were visualized with violin-box plots, and the 5′ UTR was analyzed in 10 bp windows to capture CpG variation near the leader sequence.

### RNA IP (RIP) assay of ZC3HAV1 binding to genomic and subgenomic RNAs of FIPV

To verify whether ZC3HAV1 targets coronavirus gRNA and sgRNA, RIP was performed using the BeyoRIP RIP Assay Kit (P0107; Beyotime) following the manufacturer’s instructions. CRFK cells were seeded in 6-well plates and transfected at 90% confluence with 2 μg of pCAGGS-ZC3HAV1, pCAGGS-ZC3HAV1-Z2 (RBD domain), pCAGGS-ZC3HAV1-Z4 (WWE+PARP-like domains), pCMV14-Flag-FIPV N, or pCAGGS-Flag, with non-transfected cells as controls. After 20 h, cells were infected with FIPV (MOI = 0.01) for 16 h. Lysates were prepared in RIP lysis buffer supplemented with dithiothreitol (DTT), RNase inhibitor, and phenylmethanesulfonyl fluoride (PMSF), followed by IP using anti-Flag antibody (F1804; Sigma-Aldrich) or mouse IgG isotype control (M07001; Abmart, Berkeley Heights, NJ, USA) prebound to Protein A/G agarose beads (P2012; Beyotime). RNA was eluted at 55°C and purified using RNAeasy Animal RNA Extraction Kit (R0027; Beyotime), with 20 μL reserved for input validation by WB. Co-precipitated RNA was reverse transcribed and analyzed by qPCR to detect FIPV N sgRNA. The relative enrichment of target RNAs in RIP samples was calculated using the %Input and Fold Change methods: ΔCtRIP = CtRIP−(CtInput−log_2_(Input dilution factor)); %Input = 2^^−ΔCtRIP^; ΔΔCtRIP/Flag = CtRIP − CtFlag; Fold Change = 2^^−ΔΔCtRIP/Flag^.

### Competitive RIP assay to evaluate interference of FIPV N protein with ZC3HAV1–viral RNA interaction

To investigate whether FIPV N protein can antagonize the interaction between ZC3HAV1 and viral sgRNA, a competitive RIP assay was performed in vitro. Since N protein binds to viral RNA and ZC3HAV1 directly interacts with N protein, the experimental procedures were optimized to avoid interference from N protein binding to FIPV RNA. Briefly, CRFK cells were transfected with pCAGGS-ZC3HAV1, pCMV14-Myc, and pCMV14-Myc-N plasmids and incubated for 24 h before harvesting. The cells were lysed using lysis buffer containing DTT, RNase inhibitor, and PMSF, and the cell lysates were collected. For RNA preparation, CRFK cells were infected with FIPV (MOI = 0.01) for 16 h, and total RNA was extracted using the RNAeasy Animal RNA Extraction Kit. Protein A/G Agarose was pre-incubated with HA antibody to capture ZC3HAV1. The protein complexes were then incubated with cell lysates expressing ZC3HAV1 for 1 h at 25°C, followed by washing. Subsequently, Myc-N protein was added to the experimental group and Myc protein to the control group, and the mixtures were incubated for an additional 1 h. After washing, FIPV RNA (5 µg) was added to both groups, and the reactions were incubated for 4 h at 4°C. RIP RNA was analyzed using the ΔΔCt method to calculate the enrichment fold change of FIPV genomic RNA (gRNA) and sgRNA in both the experimental and control groups. This experimental setup allowed for the assessment of whether FIPV N protein competes with ZC3HAV1 for binding to viral RNA.

To determine whether FIPV N protein could displace viral RNA from preformed ZC3HAV1–RNA complexes, HA-ZC3HAV1 protein complexes were immunoprecipitated from lysates of transfected CRFK cells using anti-HA antibody pre-bound to Protein A/G agarose beads. Cells transfected with empty HA vector (HA control) were included as negative controls. A total of 5 µg RNA, extracted from CRFK cells infected with FIPV (MOI = 0.01) for 16 h, was incubated with bead-bound HA-ZC3HAV1 protein for 4 h at 4°C to allow formation of ZC3HAV1–RNA complexes. After washing, equivalent amounts of Myc-N, Myc-Mut-N, or Myc proteins were added. Samples were incubated for an additional 1 h at 25°C. After extensive washing, RNA was extracted from the immunoprecipitated beads using the RNAeasy Animal RNA Extraction Kit. Viral RNAs were quantified by RT-qPCR, and the abundance of FIPV gRNA and sgRNAs was normalized to input RNA.

To evaluate whether FIPV N protein can disrupt ZC3HAV1 binding to host mRNA, HSPG2 mRNA, a previously reported ZC3HAV1-associated host transcript, was used as a representative host target. The same procedure was performed, except that the RNA used for complex formation was 5 µg total RNA extracted from uninfected CRFK cells. All subsequent steps were performed as described above. HSPG2 mRNA levels were quantified by RT-qPCR.

### Analysis of the antagonistic effect of FIPV N on ZC3HAV1-mediated viral suppression

To assess whether FIPV N protein can antagonize the antiviral effect of ZC3HAV1, we used JEV as a model. ZC3HAV1 has been shown to target the 3¢ UTR of JEV RNA to inhibit replication. BHK-21 cells were transfected with pCAGGS-Flag, pCMV14-Flag-FIPV N, pCAGGS-ZC3HAV1, or combinations of these plasmids. After 12 h, cells were infected with JEV (MOI = 0.1) and collected at 12, 24, and 36 hpi. WB analysis was performed to measure JEV NS1 protein expression, indicating viral replication levels. This setup allowed an examination of whether FIPV N protein can counteract the antiviral activity of ZC3HAV1.

### AlphaFold3 structural prediction

The interaction model of FIPV N and ZC3HAV1 was predicted using the AlphaFold3 online server. The confidence of the predicted structure was evaluated using the predicted local distance difference test (pLDDT) score provided by AlphaFold3, with pLDDT > 90 indicating very high confidence, 70–90 indicating confident prediction, 50–70 indicating low confidence, and < 50 indicating very low confidence. The pLDDT score was visualized by color coding in the predicted model. The resulting structures were visualized using PyMOL. Because the predicted complex was not validated by residue-level mutagenesis or structural experiments, the AlphaFold3 model was used to guide interpretation of the domain-mapping results rather than to define experimentally confirmed amino acid contacts.

### Statistical analysis

All experimental data were statistically analyzed using GraphPad Prism version 9 (GraphPad Software, San Diego, CA, USA). Differences between groups were evaluated using multiple t-tests, with p < 0.05 considered statistically significant.

## Results

### Proteomic profiling identifies ZC3HAV1 as a key N-associated host restriction factor

**Fig 1:**
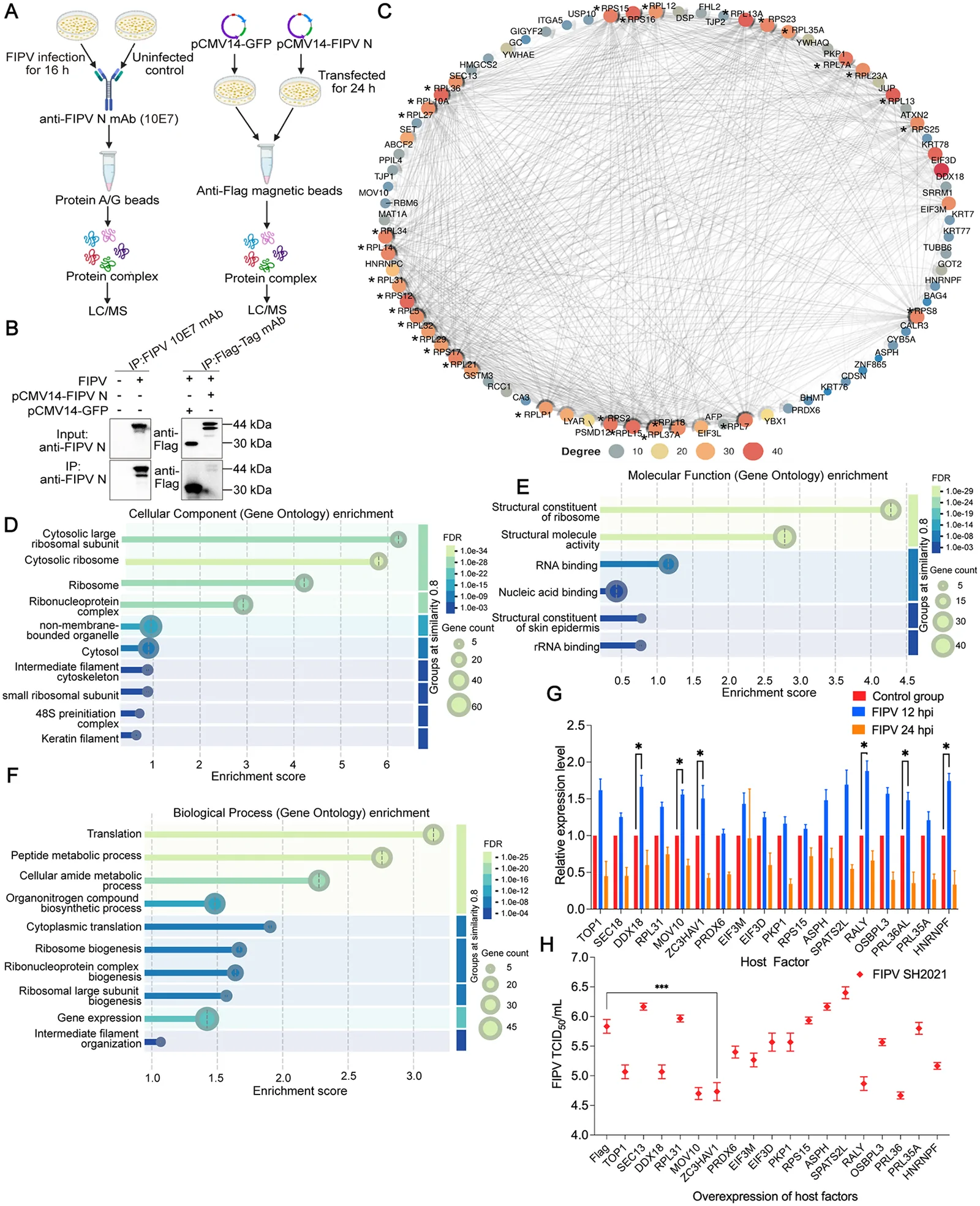
Identification and characterization of host proteins associated with the FIPV N protein. **(A)** Schematic workflow of the co-immunoprecipitation and mass spectrometry (Co-IP/MS) strategy used to identify host proteins associated with the FIPV nucleocapsid (N) protein. In the infection-based workflow, FIPV-infected and uninfected CRFK cells were subjected to immunoprecipitation using the anti-FIPV N monoclonal antibody 10E7. In the transfection-based workflow, cells expressing pCMV14-FIPV N or pCMV14-GFP were subjected to immunoprecipitation using anti-Flag magnetic beads. Immunoprecipitated protein complexes were analyzed by LC–MS. Created in BioRender. Li, **N.** (2026) https://BioRender.com/lc4m22y. **(B)** Western blot validation of N protein immunoprecipitation. In the infection-based assay, endogenous FIPV N was immunoprecipitated using anti-FIPV N monoclonal antibody 10E7 and detected using anti-FIPV N antibody. In the transfection-based assay, Flag-tagged proteins were immunoprecipitated using anti-Flag antibody and detected using anti-Flag antibody. **(C)** STRING-based protein–protein interaction (PPI) network of host proteins identified from the N-associated protein dataset. Ribosomal proteins of the RPL and RPS families and representative translation-related factors are labeled in the network. Asterisks indicate ribosomal proteins highlighted in the text. Node size indicates degree. **(D–F)** Gene Ontology (GO) enrichment analyses of N-associated host proteins categorized by cellular component **(D)**, molecular function **(E)**, and biological process **(F)**. **(G)** RT-qPCR analysis of relative mRNA expression levels of selected host genes in control and FIPV-infected CRFK cells at 12 and 24 h post-infection. **(H)** TCID₅₀ assay measuring infectious FIPV titers in CRFK cells overexpressing the indicated host factors. Data are presented as mean ± SEM (n = 3). Statistical significance: *P < 0.05, ***P < 0.001.

To systematically map host factors interacting with the FIPV nucleocapsid (N) protein, we employed two complementary co-immunoprecipitation (Co-IP) strategies coupled with mass spectrometry (MS) (Fig 1A). In the first approach, endogenous N-associated host proteins were co-precipitated from FIPV-infected CRFK cells using the anti-N monoclonal antibody 10E7. In parallel, ectopically expressed Flag-tagged N was immunoprecipitated from transfected cells with an anti-Flag antibody to capture host interactors under controlled expression conditions. Western blot analysis confirmed that both antibodies efficiently and specifically pulled down N along with associated host proteins, with minimal nonspecific background in the controls (Fig 1B), validating the reliability of both pipelines for proteomic screening of N-interacting host factors.

Label-free LC–MS analysis identified 2,197, 1,975, 3,589, and 3,630 protein entries in the CRFK, CRFK + FIPV, pCMV14-FIPV N, and pCMV14-GFP groups, respectively (S5 Table). Notably, the number of protein entries detected in FIPV-infected cells was approximately 10% lower than that in uninfected cells. This reduction may be attributed to multiple factors, including virus-induced alterations in host protein abundance, solubility, or subcellular localization, competition or epitope-masking effects during immunoprecipitation, and infection-associated changes in sample complexity or cellular stress. To define candidate N-associated host proteins, we calculated the enrichment ratio for each protein relative to its corresponding control. Proteins with an enrichment ratio >2 in the FIPV-infected anti-N IP sample relative to the uninfected CRFK control IP sample, or in the Flag-FIPV N IP sample relative to the Flag-GFP control IP sample, were retained as candidate interactors. Proteins detected exclusively in the control samples or lacking enrichment over the corresponding background control were excluded as nonspecific background proteins. The filtered protein lists from the infection- and transfection-based datasets were then integrated and manually curated to remove redundant entries, resulting in a final set of 98 high-confidence N-associated host proteins for subsequent functional analyses.

To explore the biological context of host factors interacting with the FIPV N protein, we constructed a protein–protein interaction (PPI) network using the STRING database (Fig 1C). The network revealed a highly interconnected architecture dominated by explicitly labeled ribosomal proteins of the RPL and RPS families, together with representative translation-related factors such as EIF3D and EIF3M, indicating that N interacts extensively with the host translational machinery. Several RNA-associated factors, including ZC3HAV1, MOV10, HNRNPF, DDX18, and RALY, also occupied central nodes within the network, suggesting potential links between viral RNA handling and host RNA-binding processes.

Gene Ontology (GO) enrichment analysis further characterized the molecular features of these interactors across three functional dimensions. In the cellular component category, proteins were mainly enriched in the cytosolic ribosome and ribonucleoprotein complexes (Fig 1D), indicating that many N-associated host factors are integral parts of translation assemblies. In the molecular function category, enrichment was dominated by structural constituents of ribosomes, RNA-binding, and nucleic acid-binding activities (Fig 1E), reflecting a strong association with RNA metabolism and translational regulation. In the biological process category, significant enrichment was observed for translation, peptide and amide metabolic processes, cytoplasmic translation, and ribosome biogenesis (Fig 1F), revealing that N-interacting proteins converge functionally on protein synthesis and RNA processing pathways.

Together, these integrated proteomic and bioinformatic analyses demonstrate that the FIPV N protein engages a dense network of host factors involved in translation and RNA metabolism—two pathways that often overlap with intrinsic post-transcriptional restriction and antiviral RNA surveillance. This suggests that N may occupy a pivotal functional interface between viral RNA synthesis and host RNA-regulatory systems, potentially enabling the virus to couple efficient genome replication with evasion of cellular RNA-level restriction. From the 98 N-associated host proteins identified by MS analysis, we next prioritized 18 candidates based on their annotated functions in RNA metabolism, translation regulation, or antiviral defense S6 Table. Transcriptional profiling during FIPV infection revealed a characteristic biphasic pattern—most genes were transiently upregulated at 12 h post-infection and subsequently downregulated by 24 h (Fig 1G). This temporal pattern may represent an initial host effort to stabilize RNA metabolism and restrict viral replication, which is subsequently overridden as the virus gains control over the host translational machinery.

To evaluate their functional relevance, each of the 18 candidates was cloned into pCAGGS for overexpression in CRFK cells, followed by FIPV challenge and viral titer quantification. Six RNA-associated proteins—DDX18, MOV10, RPL36, ZC3HAV1, HNRNPF, and RALY—significantly reduced viral replication (Fig 1H), identifying them as putative restriction factors within the N interactome. Notably, several of these factors, such as MOV10 and ZC3HAV1, have been implicated in post-transcriptional antiviral restriction in other RNA viruses [19,20], reinforcing the possibility that FIPV engages components of the host RNA surveillance network.

Among these, ZC3HAV1 stood out for its well-established role in RNA-targeted restriction across diverse viral families, including coronaviruses [18,21,22]. Its dual identification as an N-associated host protein and as a negative regulator of FIPV replication prompted us to investigate how ZC3HAV1 restricts FIPV and how FIPV N counteracts this restriction.

### ZC3HAV1 restricts FIPV replication through an IFN-independent RNA-level mechanism

**Fig 2:**
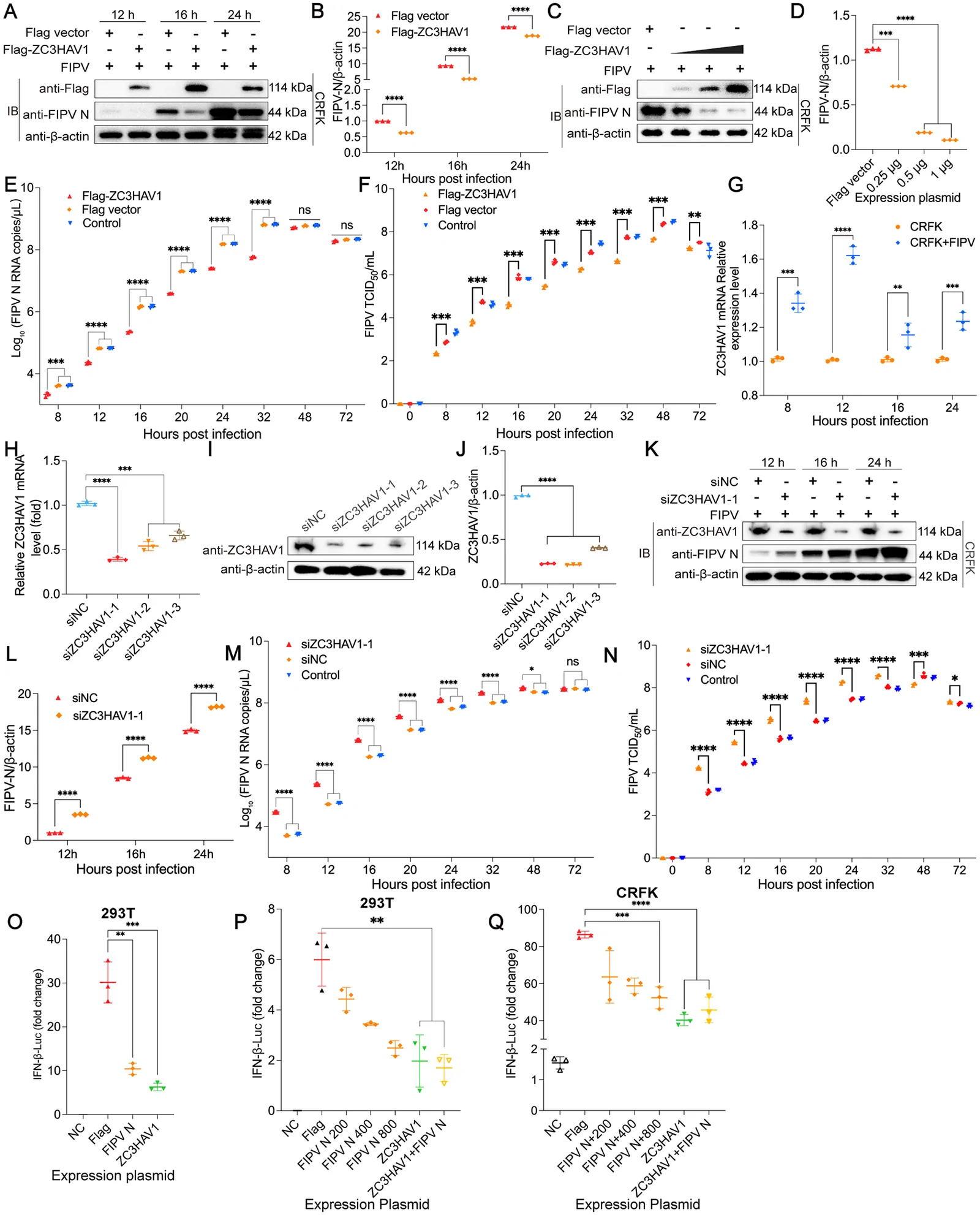
ZC3HAV1 suppresses FIPV replication through an IFN-independent antiviral mechanism. **(A)** Western blot analysis of FIPV N protein expression in FIPV-infected CRFK cells transfected with Flag vector or Flag-ZC3HAV1 at 12, 16, and 24 h post-infection (hpi). Flag-ZC3HAV1 was detected using anti-Flag antibody, FIPV N was detected using anti-FIPV N antibody, and β-actin served as the loading control. **(B)** Densitometric quantification of FIPV N protein levels shown in panel **A. (C)** Western blot analysis of FIPV N protein expression in FIPV-infected CRFK cells transfected with increasing amounts of Flag-ZC3HAV1 plasmid. **(D)** Densitometric quantification of FIPV N protein levels shown in panel **C. (E)** Absolute RT-qPCR analysis of FIPV N RNA copies in FIPV-infected CRFK cells assigned to the Control, Flag vector, or Flag-ZC3HAV1 group. Here, Control refers to FIPV-infected cells without plasmid transfection. **(F)** TCID₅₀ assay measuring infectious FIPV titers in the same treatment groups as in panel **E. (G)** Time-course RT-qPCR analysis of endogenous ZC3HAV1 mRNA expression in CRFK cells with or without FIPV infection. **(H)** RT-qPCR analysis of ZC3HAV1 mRNA levels in CRFK cells transfected with control siRNA or ZC3HAV1-targeting siRNAs. **(I)** Western blot analysis of endogenous ZC3HAV1 protein levels following siRNA-mediated knockdown. ZC3HAV1 was detected using anti-ZC3HAV1 antibody, and β-actin served as the loading control. **(J)** Densitometric quantification of ZC3HAV1 protein levels shown in panel **I. (K)** Western blot analysis of FIPV N protein expression in CRFK cells transfected with control siRNA or siZC3HAV1-1, followed by FIPV infection at 12, 16, and 24 hpi. Endogenous ZC3HAV1 was detected using anti-ZC3HAV1 antibody, FIPV N was detected using anti-FIPV N antibody, and β-actin served as the loading control. **(L)** Densitometric quantification of FIPV N protein levels shown in panel **K. (M)** Absolute RT-qPCR analysis of FIPV N RNA copies in FIPV-infected CRFK cells assigned to the Control, siNC, or siZC3HAV1-1 group. Here, Control refers to FIPV-infected cells without siRNA transfection. **(N)** TCID₅₀ assay measuring infectious FIPV titers in the same treatment groups as in panel **M. (O)** Dual-luciferase reporter assay measuring Sendai virus (SeV)-induced IFN-β promoter activity in 293T cells expressing Flag vector, FIPV N, or ZC3HAV1. **(P–Q)** Dual-luciferase reporter assays measuring SeV-induced IFN-β promoter activity in 293T (P) and CRFK (Q) cells co-expressing ZC3HAV1 and increasing amounts of FIPV **N.** In panels P and Q, N 200, N 400, and N 800 indicate transfection with 200 ng, 400 ng, and 800 ng of the FIPV N expression plasmid, respectively. Data are presented as mean ± SEM (n = 3). Statistical significance: *P < 0.05, **P < 0.01, ***P < 0.001, ****P < 0.0001; ns, not significant.

Having identified ZC3HAV1 as a potent N-binding restriction factor, we next sought to validate its antiviral activity and elucidate its mechanism of action. To determine whether the host restriction factor ZC3HAV1 affects FIPV replication, CRFK cells were transfected with a ZC3HAV1 expression plasmid and subsequently infected with FIPV (MOI = 0.01). Samples were collected at indicated time points for Western blotting, TCID₅₀ assays, and absolute RT–qPCR quantification. Western blot analysis showed that ZC3HAV1 overexpression markedly suppressed FIPV replication, as reflected by the decreased N protein abundance at 12, 16, and 24 hpi (Fig 2A–B). A dose-dependent reduction in N expression was also observed upon increasing amounts of ZC3HAV1 plasmid (Fig 2C–D). Absolute RT–qPCR confirmed a significant decrease in viral N transcripts from 8 to 32 h (Fig 2E), while TCID₅₀ assays further demonstrated a corresponding reduction in viral titers (Fig 2F). Together, these data indicate that ZC3HAV1 acts as a potent suppressor of FIPV replication.

Endogenous ZC3HAV1 mRNA expression increased following FIPV infection, with the highest induction observed at 12 hpi and sustained elevation still detectable at 24 hpi (Fig 2G). These data indicate that ZC3HAV1 is transcriptionally responsive to FIPV infection. Because ectopically expressed ZC3HAV1 showed reduced protein abundance at later infection time points (Fig 2A), we further examined whether this decrease could be attributed to direct degradation mediated by FIPV N. Co-transfection of HA-ZC3HAV1 with increasing amounts of Flag-FIPV N did not noticeably reduce HA-ZC3HAV1 protein levels, and densitometric analysis showed no significant decrease in HA-ZC3HAV1 abundance (S1A-B Fig). These results do not support a model in which FIPV N alone directly induces ZC3HAV1 degradation.

We also examined endogenous ZC3HAV1 protein levels during FIPV infection. In contrast to the late decrease observed for ectopically expressed ZC3HAV1, endogenous ZC3HAV1 protein was increased in FIPV-infected cells compared with mock-infected cells at 12, 16, and 24 hpi, accompanied by increasing FIPV N expression as a marker of productive infection (S1C-D Fig). Together with the ZC3HAV1 mRNA induction shown in Fig 2G, these data indicate that FIPV infection induces endogenous ZC3HAV1 expression rather than suppressing it. Therefore, the late decrease in ectopically expressed ZC3HAV1 likely reflects late post-transfection protein turnover or infection-associated changes in cellular protein homeostasis, rather than direct degradation by FIPV N.

To further validate its inhibitory role, three siRNAs were designed to knock down ZC3HAV1 expression. Among them, siZC3HAV1–1 achieved the highest silencing efficiency at both mRNA and protein levels (Fig 2H–J). Knockdown of ZC3HAV1 significantly enhanced FIPV replication, as evidenced by increased N protein expression (Fig 2K–L), elevated viral RNA levels (Fig 2M), and higher viral titers (Fig 2N). These complementary overexpression and knockdown results consistently support ZC3HAV1 as a negative regulator of FIPV replication.

Because ZC3HAV1 is an interferon-stimulated gene (ISG), its antiviral activity could either arise indirectly through type I interferon (IFN) pathway activation or directly through intrinsic RNA-targeted restriction. To distinguish between these possibilities, we next examined whether the antiviral effect of ZC3HAV1 depends on IFN signaling activation or instead reflects an intrinsic post-transcriptional restriction mechanism. Dual-luciferase reporter assays revealed that both ZC3HAV1 and the FIPV N protein independently suppressed Sendai virus (SeV)–induced IFN-β promoter activity in 293T cells (Fig 2O). However, co-expression experiments in both 293T and CRFK cells showed no synergistic or antagonistic interaction between the two proteins in regulating IFN-β transcription (Fig 2P–Q). These results indicate that, under the conditions of this reporter assay, ZC3HAV1-mediated suppression of FIPV replication is unlikely to be explained by type I interferon (IFN) pathway activation. We therefore next examined whether ZC3HAV1 restricts FIPV through direct association with viral RNA.

### ZC3HAV1 reduces the accumulation and persistence of FIPV genomic and subgenomic RNAs

**Fig 3:**
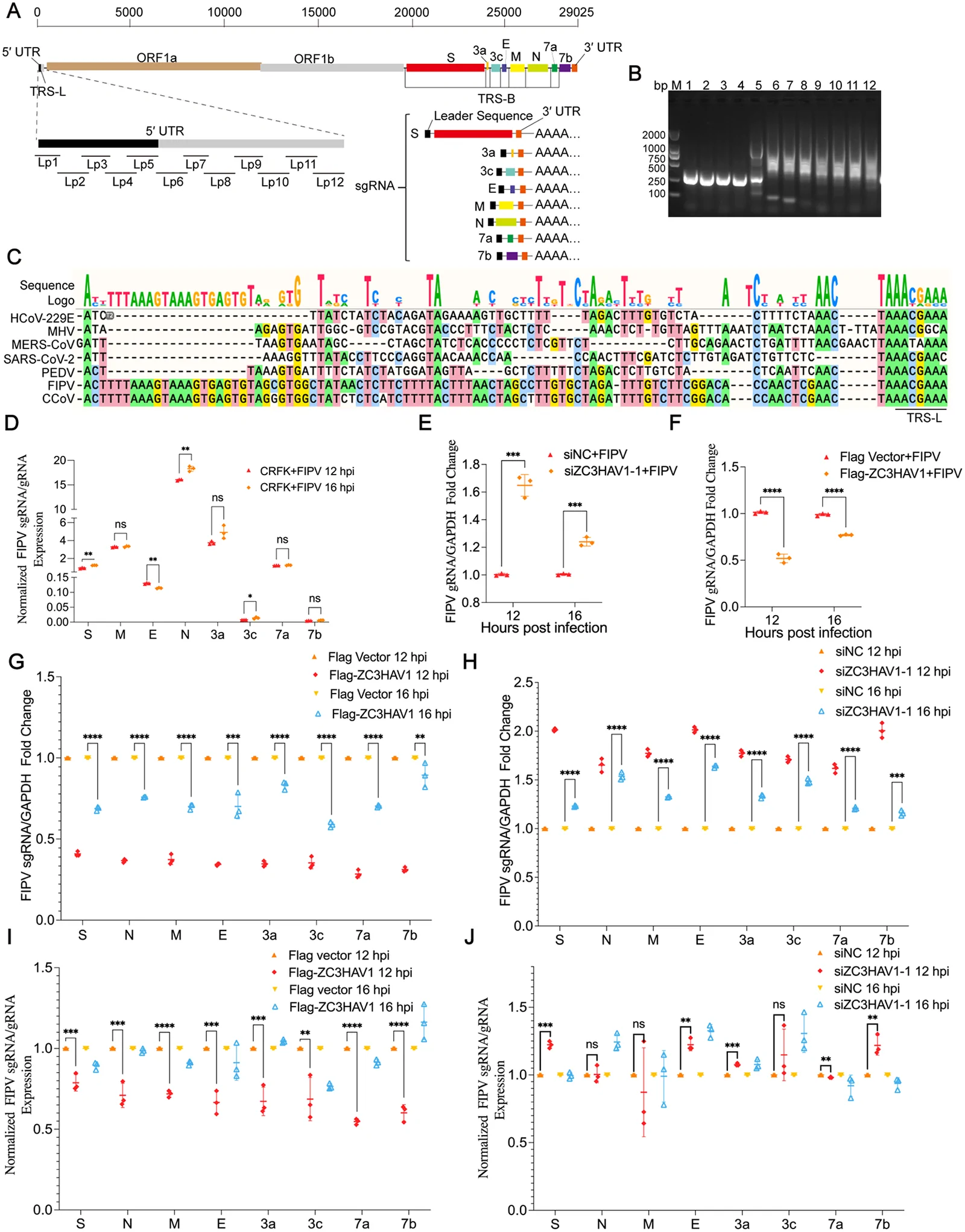
ZC3HAV1 reduces the accumulation and persistence of FIPV genomic and subgenomic RNAs. **(A)** Schematic of primer design strategy targeting the FIPV 5′ UTR leader sequence. **(B)** PCR amplification identifying the leader-containing region using Lp1–Lp12 forward primers paired with N-specific reverse primer. **(C)** Sequence alignment of the 5’ UTR leader region between FIPV and representative coronaviruses. **(D)** RT-qPCR analysis of normalized FIPV sgRNA/gRNA expression ratios for structural and accessory genes at 12 and 16 h post-infection (hpi). **(E)** RT-qPCR analysis of FIPV gRNA levels in CRFK cells transfected with siNC or siZC3HAV1-1, followed by FIPV infection. **(F)** RT-qPCR analysis of FIPV gRNA levels in CRFK cells transfected with Flag vector or Flag-ZC3HAV1, followed by FIPV infection. **(G)** RT-qPCR analysis of FIPV sgRNA levels in Flag vector- or Flag-ZC3HAV1-transfected cells following FIPV infection. **(H)** RT-qPCR analysis of FIPV sgRNA levels in siNC- or siZC3HAV1-1-transfected cells following FIPV infection. **(I)** Relative sgRNA/gRNA expression ratios following ZC3HAV1 overexpression. **(J)** Relative sgRNA/gRNA expression ratios following ZC3HAV1 knockdown. For this analysis, each sgRNA level was normalized to the corresponding gRNA level from the same sample. This normalization controls for differences in overall viral genome abundance and allows comparison of the relative sgRNA composition under different ZC3HAV1 expression conditions. Data are presented as mean ± SEM (n = 3). *P < 0.05, **P < 0.01, ***P < 0.001, ****P < 0.0001; ns, not significant.

Given its potent antiviral activity, we next investigated whether ZC3HAV1 restricts FIPV replication at the RNA level through direct interference with viral transcript accumulation. To this end, we first established a quantitative detection system that distinguishes between genomic RNA (gRNA) and individual subgenomic RNAs (sgRNAs) produced through the coronavirus discontinuous transcription mechanism. As illustrated in Fig 3A, a series of forward primers (Lp1–Lp12) within the 5’ UTR were paired with a reverse primer located at the 5’ end of the N gene. PCR amplification using viral cDNA as the template yielded distinct products with Lp1–Lp5 (Fig 3B), indicating that the leader sequence of FIPV resides within this region. Sequence alignment with representative coronaviruses revealed that the FIPV transcription-regulatory sequence in the leader (TRS-L) is ACGAAA, and the complete leader sequence was determined (Fig 3C).

Based on this mapping, we designed specific primer sets for each sgRNA (S, 3a, 3c, E, M, N, 7a, and 7b) and one for gRNA detection. Each pair amplified a single, expected band, confirming primer specificity and establishing a robust assay for quantifying FIPV gRNA and sgRNAs.

We next analyzed the relative accumulation pattern of FIPV sgRNAs by normalizing each sgRNA to gRNA levels. Because gRNA reflects overall viral genome abundance and serves as the template for sgRNA synthesis, the sgRNA/gRNA ratio provides a normalized readout of sgRNA accumulation after accounting for differences in total viral RNA levels. Among the detected sgRNAs, the N sgRNA showed the highest sgRNA/gRNA ratio, followed by M and S, whereas several accessory sgRNAs displayed lower relative abundance (Fig 3D). This pattern is consistent with canonical coronavirus discontinuous transcription, in which structural gene sgRNAs generally accumulate more prominently than accessory transcripts. The sgRNA/gRNA ratios of most transcripts remained broadly comparable between 12 h and 16 h post-infection, with only modest transcript-specific fluctuations (Fig 3D). Thus, Fig 3D establishes a reference profile of sgRNA-to-gRNA relationships during FIPV infection, allowing us to assess whether ZC3HAV1 alters overall viral RNA accumulation or selectively reshapes the relative sgRNA profile.

To assess whether ZC3HAV1 influences these viral RNAs, CRFK cells were transfected with ZC3HAV1 expression plasmids or siRNAs prior to infection. Overexpression of ZC3HAV1 markedly reduced FIPV gRNA levels at 12 h and 16 h (Fig 3E), whereas its knockdown had the opposite effect (Fig 3F). A similar pattern was observed for sgRNAs: ZC3HAV1 overexpression significantly decreased, and knockdown increased, the transcript abundance of multiple structural and accessory sgRNAs (Fig 3G–H). These data indicate that ZC3HAV1 reduces the steady-state accumulation of both genomic and subgenomic FIPV RNAs.

Because gRNA serves as the template for sgRNA synthesis, we further normalized sgRNA abundance to gRNA levels to evaluate whether ZC3HAV1 altered the relative sgRNA profile independently of changes in overall viral genome abundance. Although ZC3HAV1 overexpression reduced, and ZC3HAV1 knockdown increased, the absolute levels of both gRNA and multiple sgRNAs (Fig 3E–H), the sgRNA/gRNA ratios showed only limited changes for most sgRNA species (Fig 3I–J). These results suggest that ZC3HAV1 primarily reduces overall FIPV RNA accumulation rather than markedly reshaping the relative abundance of individual sgRNAs. Thus, the decrease in sgRNA levels is largely consistent with a broad reduction in viral RNA abundance, rather than a selective alteration in the relative sgRNA composition.

However, steady-state RT-qPCR analysis alone cannot determine whether reduced viral RNA abundance results from impaired viral RNA synthesis or from reduced persistence of already synthesized viral RNAs. To determine whether ZC3HAV1 affects the persistence of already synthesized viral RNAs, we performed a GS-441524-based RNA persistence assay after FIPV RNAs had accumulated. GS-441524 treatment effectively suppressed the continued accumulation of FIPV N RNA and reduced normalized sgRNA/gRNA expression over time, confirming that ongoing RdRp-dependent RNA synthesis was strongly inhibited under these conditions (S2A–B Fig). In this replication-suppressed setting, ZC3HAV1 overexpression reduced the levels of FIPV gRNA and multiple sgRNAs, whereas ZC3HAV1 knockdown increased their abundance (S2C–E Fig). After normalization to gRNA, ZC3HAV1 overexpression also decreased, whereas ZC3HAV1 knockdown increased, the relative abundance of several sgRNAs (S2F–G Fig). These results indicate that ZC3HAV1-mediated restriction is not explained solely by reduced viral RNA synthesis or altered sgRNA transcription, but involves reduced persistence of already synthesized FIPV genomic and subgenomic RNAs.

Interestingly, the suppressive effect of ZC3HAV1 on viral RNA accumulation weakened at later infection time points, suggesting that FIPV may counteract ZC3HAV1-associated RNA-level restriction as infection progresses. Together, these findings identify ZC3HAV1 as a restriction factor that limits FIPV genomic and subgenomic RNA accumulation, at least in part, by reducing viral RNA persistence after RNA synthesis has occurred. Although these data are consistent with destabilization or degradation of ZC3HAV1-associated viral RNAs, they do not define the precise downstream RNA-decay machinery involved. Together, these findings identify ZC3HAV1 as a restriction factor that limits FIPV genomic and subgenomic RNA accumulation primarily by reducing viral RNA persistence after RNA synthesis has occurred. Because our proteomic analysis identified FIPV N as a major ZC3HAV1-associated viral protein, this RNA-level restriction phenotype provided the rationale for examining whether FIPV N counteracts ZC3HAV1-mediated control of viral RNA persistence.

### FIPV N engages ZC3HAV1 and counteracts ZC3HAV1-mediated antiviral restriction

**Fig 4:**
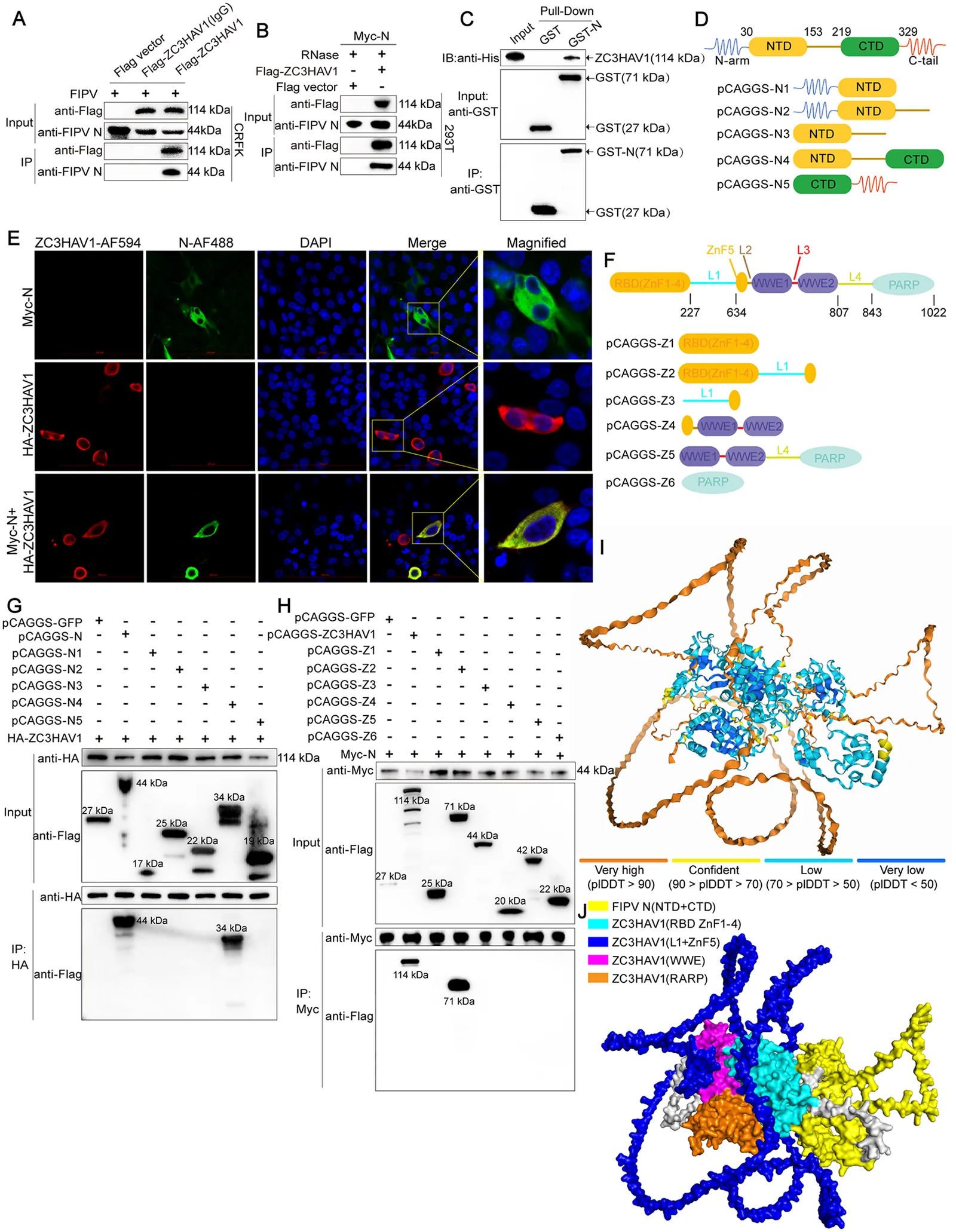
ZC3HAV1 directly interacts with the FIPV N protein via its RBD-containing domain. **(A)** Co-immunoprecipitation analysis of Flag-ZC3HAV1 and infection-derived FIPV N in FIPV-infected CRFK cells. Flag-ZC3HAV1 was immunoprecipitated using anti-Flag antibody, with IgG serving as the immunoprecipitation control, and the recovered proteins were detected using anti-Flag and anti-FIPV N antibodies. **(B)** Co-immunoprecipitation analysis of Flag-ZC3HAV1 and FIPV N in 293T cells co-expressing Flag-ZC3HAV1 and Myc-N, with or without RNase treatment. Flag-ZC3HAV1 was immunoprecipitated using anti-Flag antibody, and immunoprecipitated proteins were detected using anti-Flag and anti-Myc antibodies. **(C)** GST pull-down assay using purified GST, GST-N, and His-ZC3HAV1 proteins. His-ZC3HAV1 was detected using anti-His antibody, and GST or GST-N was detected using anti-GST antibody. **(D)** Schematic diagram of full-length and truncated FIPV N constructs used for domain mapping. **(E)** Confocal microscopy analysis of overexpressed HA-ZC3HAV1 and Myc-N in transfected cells. ZC3HAV1 was detected with AF594, FIPV N was detected with AF488, and nuclei were stained with DAPI. Magnified images show the boxed regions. **(F)** Schematic representation of ZC3HAV1 functional regions and truncation constructs used for domain mapping. The five CCCH-type zinc finger motifs within the RBD are labeled ZnF1–ZnF5 to distinguish them from the ZC3HAV1 truncation constructs Z1–Z6. **(G)** Co-immunoprecipitation analysis of HA-ZC3HAV1 binding to full-length and truncated FIPV N constructs. GFP was included as an unrelated protein negative control. HA-ZC3HAV1 was immunoprecipitated using anti-HA antibody, and recovered N constructs were detected using anti-Flag antibody. **(H)** Co-immunoprecipitation analysis of Myc-N binding to full-length and truncated ZC3HAV1 constructs. GFP was included as an unrelated protein negative control. Myc-N was immunoprecipitated using anti-Myc antibody, and recovered ZC3HAV1 constructs were detected using anti-Flag antibody. **(I)** AlphaFold3-predicted structural model of the FIPV N–ZC3HAV1 complex, with the pLDDT confidence score indicated by color. **(J)** PyMOL visualization of the predicted interaction model highlighting the putative interface between FIPV N and the ZC3HAV1 RBD-containing region. Specific interacting residues were not annotated because residue-level contacts were not experimentally validated in this study.

To test our hypothesis that FIPV N interferes with ZC3HAV1-associated RNA-level restriction, we next examined whether N interacts with ZC3HAV1. In CRFK cells infected with FIPV (MOI = 0.01), co-immunoprecipitation (Co-IP) assays revealed that Flag-tagged ZC3HAV1 efficiently pulled down the endogenous N protein, whereas no interaction was detected in IgG or vector controls (Fig 4A). This interaction was further confirmed in 293T cells co-expressing Myc-tagged N and Flag-ZC3HAV1, indicating that their association is not limited to the CRFK cell background (Fig 4B). Importantly, treatment with RNase did not abolish the interaction, suggesting that the association is not primarily mediated by RNA bridging (Fig 4B, right).

To further examine whether ZC3HAV1 and FIPV N can associate in vitro, His-ZC3HAV1 was incubated with clarified soluble bacterial lysates containing GST-N or GST control protein. GST pull-down followed by immunoblotting showed that His-ZC3HAV1 was recovered with GST-N but not with GST alone, supporting an in vitro association between ZC3HAV1 and FIPV N under the conditions of this assay (Fig 4C). Confocal immunofluorescence analysis showed partial cytoplasmic overlap between overexpressed ZC3HAV1 and FIPV N (Fig 4E), consistent with the possibility that the interaction occurs in the cytoplasm, where viral RNA replication and RNA-level restriction take place. To further evaluate whether this spatial association could also be observed under infection conditions, we examined endogenous ZC3HAV1 and infection-derived FIPV N in CRFK cells. In uninfected CRFK cells, endogenous ZC3HAV1 showed cytoplasmic staining, whereas FIPV N staining was not detected. Upon FIPV infection, N protein accumulated mainly in the cytoplasm and showed partial overlap with endogenous ZC3HAV1-positive signals (S3 Fig). These data support the infection-context relevance of the ZC3HAV1–N spatial association observed in the overexpression system.

To map the regions required for this interaction, we performed reciprocal domain mapping using truncation mutants of both proteins. For FIPV N, six constructs were generated (N1–N6) encompassing different combinations of its N-terminal domain (NTD) and C-terminal domain (CTD) (Fig 4D). To exclude nonspecific recovery of overexpressed proteins, GFP was included as an unrelated protein negative control in the Co-IP assay. Co-IP assays revealed that ZC3HAV1 interacted efficiently with full-length N and the N4 fragment containing both NTD and CTD, whereas GFP and N fragments lacking either region showed weak or undetectable recovery (Fig 4F). Thus, efficient association with ZC3HAV1 was observed only when both the NTD and CTD were present. These data suggest that the structural integrity of both the NTD and CTD is required for efficient interaction with ZC3HAV1. Because deletion of either domain may also affect N folding, oligomerization, or RNA-binding properties, these truncation results were interpreted as supportive domain-mapping evidence rather than definitive identification of a minimal binding interface.

Similarly, ZC3HAV1 truncation constructs were designed to refine the N-interacting region. The RBD of ZC3HAV1 contains five CCCH-type zinc finger motifs, designated ZnF1–ZnF5, whereas the ZC3HAV1 truncation constructs are designated Z1–Z6 (Fig 4G). GFP was included as an unrelated protein negative control to evaluate nonspecific recovery in the Co-IP assay. FIPV N efficiently co-immunoprecipitated full-length ZC3HAV1 and the RBD-containing construct encompassing ZnF1–ZnF5, whereas the other truncation constructs were not detectably recovered (Fig 4H). These results indicate that the RBD-containing region of ZC3HAV1, which encompasses ZnF1–ZnF5, is required for efficient association with FIPV N.

To further visualize the putative molecular interface, an AlphaFold3-predicted structural model was generated and analyzed in PyMOL (Fig 4I–J). The model suggested that the NTD and CTD of the FIPV N protein may form a putative composite surface positioned near the N-terminal region of ZC3HAV1 encompassing the RBD (residues 227–634). The linker L1 and the fifth CCCH-type zinc finger motif were positioned near the predicted contact region, providing a structural hypothesis consistent with the domain-mapping results. Because this model is computationally predicted, it was used to guide interpretation of the domain-mapping data rather than as definitive structural evidence.

Together, these findings establish that FIPV N associates with ZC3HAV1 and that efficient interaction requires the RBD-containing region of ZC3HAV1 and the intact structural organization of FIPV N. We next investigated whether this interaction contributes to the ability of FIPV N to counteract ZC3HAV1-mediated antiviral restriction.

To further assess the functional relevance of the N–ZC3HAV1 interaction, we performed rescue experiments in ZC3HAV1-depleted CRFK-shZC3HAV1 cells. Full-length HA-ZC3HAV1 or HA-Z5, a truncation lacking the RBD-containing region involved in FIPV N interaction, was reconstituted in these cells, and Myc-FIPV N was co-expressed with full-length HA-ZC3HAV1 where indicated, followed by FIPV infection. Reconstitution with full-length ZC3HAV1 restored antiviral restriction, as indicated by reduced FIPV-M protein expression at 12 and 24 hpi and decreased viral titers over time (S4A–C Fig). Co-expression of FIPV N partially reversed the inhibitory effect of full-length ZC3HAV1, resulting in increased FIPV M protein abundance and higher viral titers compared with ZC3HAV1 reconstitution alone (S4A–C Fig). These results support a functional antagonistic effect of FIPV N on ZC3HAV1-mediated restriction.

In contrast, HA-Z5 showed markedly impaired antiviral activity compared with full-length ZC3HAV1, as reflected by higher FIPV M protein levels and increased viral titers (S4A–C Fig). This finding indicates that the RBD-containing region is required not only for FIPV N association but also for the intrinsic antiviral function of ZC3HAV1. Therefore, HA-Z5 was interpreted as a functionally impaired truncation rather than a strict interaction-deficient but restriction-competent mutant.

To further determine whether the ZC3HAV1-associated region of FIPV N contributes to its antagonistic activity, we performed additional functional analyses using the N truncation mutants identified in the interaction mapping assay (Fig 4D and F). Full-length N and individual N truncation mutants were evaluated in the ZC3HAV1 rescue system. Expression analysis confirmed that the N fragments were detectable under the experimental conditions, allowing functional comparison among different constructs (S4D Fig). Compared with full-length N, the antagonistic activity of individual N truncation mutants varied according to their retained structural regions. Among the truncation mutants, the N4 fragment, which showed efficient ZC3HAV1 association in the mapping assay, displayed relatively stronger capacity to relieve ZC3HAV1-mediated restriction, whereas fragments with weak or undetectable ZC3HAV1 association showed reduced antagonistic activity, as measured by viral protein accumulation and viral RNA levels (S4E and F Fig.). Consistently, analysis of infectious virus production showed a similar pattern among N truncation mutants (S4G Fig). These results suggest that the ZC3HAV1-associated region of FIPV N contributes to efficient antagonism of ZC3HAV1-mediated antiviral restriction.

Together, these rescue analyses support a model in which the N–ZC3HAV1 interaction contributes to FIPV N-mediated antagonism of host RNA-level restriction, while the mechanism by which N disrupts ZC3HAV1–viral RNA association was further investigated below.

### ZC3HAV1 targets FIPV genomic and subgenomic RNAs through its RBD-containing region despite low CpG frequency

**Fig 5:**
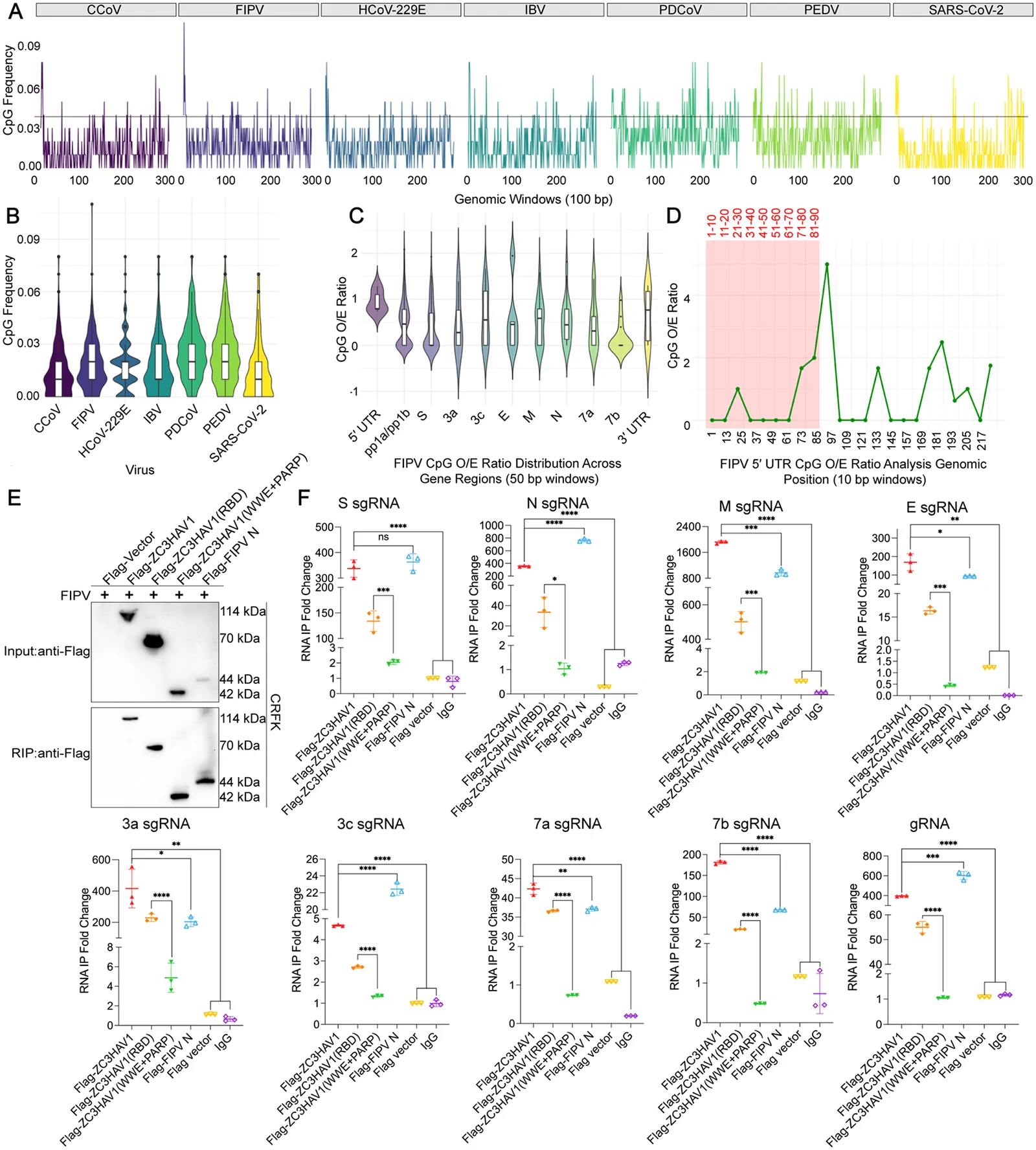
ZC3HAV1 binds FIPV genomic and subgenomic RNAs through its RBD-containing region despite the overall low CpG frequency of the FIPV genome. **(A)** Genome-wide CpG frequency distribution across representative coronaviruses. **(B)** Comparison of CpG frequency among coronavirus genomes, highlighting the low-CpG pattern of FIPV and HCoV-229E. **(C)** CpG observed/expected (O/E) ratio analysis of the FIPV genome, showing localized enrichment near the 5′ UTR. **(D)** CpG O/E ratio within the FIPV 5′ UTR. **(E)** Western blot analysis of input and immunoprecipitated fractions verifying recovery of the indicated Flag-tagged proteins used in the RIP assays, including Flag-FIPV N, full-length ZC3HAV1, the RBD-containing region, and the WWE + PARP region. Flag-tagged proteins were immunoprecipitated using anti-Flag antibody and detected by western blotting with anti-Flag antibody. **(F)** RIP–qPCR analysis of FIPV genomic RNA (gRNA) and the indicated subgenomic RNAs (sgRNAs) enriched in immunoprecipitates from cells expressing Flag-FIPV N, full-length ZC3HAV1, the RBD-containing region, or the WWE + PARP region, with IgG and Flag vector included as negative controls. Data are presented as mean ± SEM (n = 3). *P < 0.05, **P < 0.01, ***P < 0.001, ****P < 0.0001; ns, not significant.

ZC3HAV1 has been classically defined as a CpG-biased antiviral factor that preferentially associates with CpG-enriched viral RNAs and restricts their accumulation. To determine whether this canonical CpG-biased recognition model applies to FIPV, we next examined genome-wide CpG distribution patterns across FIPV and related coronaviruses. Genome-wide profiling revealed that the overall CpG frequency of FIPV was markedly low, with enrichment confined to the 5′ region (Fig 5A–B). In contrast, porcine coronaviruses such as PDCoV and PEDV displayed higher and more uniform CpG densities, while HCoV-229E and FIPV shared a similar pattern characterized by a single 5′-end CpG cluster and sparse distribution elsewhere.

A more refined window analysis of the FIPV genome (Fig 5C) confirmed a localized CpG enrichment near the 5′ untranslated region (UTR), with the remainder of the genome showing substantial CpG depletion. Because all coronavirus sgRNAs share a common 5′ leader derived from this UTR, we hypothesized that ZC3HAV1 might recognize sgRNAs through CpG-rich leader sequences. However, mapping the CpG composition within the FIPV 5′UTR (Fig 5D) revealed that CpG enrichment occurs outside the canonical leader–body junction, suggesting that ZC3HAV1–RNA recognition in FIPV is not fully explained by local CpG enrichment within the leader-containing region.

To experimentally verify whether ZC3HAV1 binds FIPV RNA, we performed RNA immunoprecipitation (RIP) assays in FIPV-infected CRFK cells overexpressing different ZC3HAV1 domain constructs—full-length ZC3HAV1, the isolated RNA-binding domain (RBD), or the WWE + PARP region—along with FIPV N and Flag controls. Western blotting confirmed efficient precipitation of each Flag-tagged construct (Fig 5E). Subsequent RT-qPCR analysis of immunoprecipitated RNA revealed that full-length ZC3HAV1 and its RBD markedly enriched multiple FIPV RNAs, including gRNA and sgRNAs encoding structural (S, M, N, E) and accessory proteins (3a, 3c, 7a, 7b) (Fig 5F). The enrichment signal varied among individual sgRNAs, with some accessory sgRNAs, such as 3c, showing relatively low recovery. This variation may reflect differences in intracellular transcript abundance, reduced assay sensitivity for low-copy RNA species, or weaker association with ZC3HAV1-containing ribonucleoprotein complexes. In contrast, the WWE + PARP fragment showed minimal enrichment, comparable to the IgG and Flag controls. These RIP data support ZC3HAV1 association with FIPV RNAs in infected cells, but do not by themselves define the precise CpG motif, RNA structure, or downstream fate of ZC3HAV1-bound viral RNAs.

Consistent with previous reports that coronavirus N proteins can associate with both genomic and subgenomic viral RNAs in infected cells, FLAG-FIPV N also enriched multiple FIPV RNA species in the RIP assay, including gRNA and several sgRNAs (Fig 5F). Because the RIP assay was performed using infected cell lysates, these signals reflect intracellular N-associated viral RNAs during infection. The relatively strong sgRNA signals are consistent with the high intracellular abundance of coronavirus sgRNAs generated during discontinuous transcription.

These findings show that ZC3HAV1 associates with FIPV genomic and subgenomic RNAs through its RBD-containing region despite the overall low CpG frequency of the FIPV genome. Given the low CpG density across most of the FIPV genome, ZC3HAV1 recognition may involve additional RNA features, such as local RNA structure, subgenomic RNA abundance, local sequence context, localized CpG-enriched regions, or viral ribonucleoprotein organization, rather than being explained solely by global CpG frequency. Thus, our data support a noncanonical or CpG-limited mode of ZC3HAV1 engagement, but do not establish complete CpG independence.

### FIPV N disrupts ZC3HAV1–viral RNA association to attenuate RNA-level restriction

**Fig 6:**
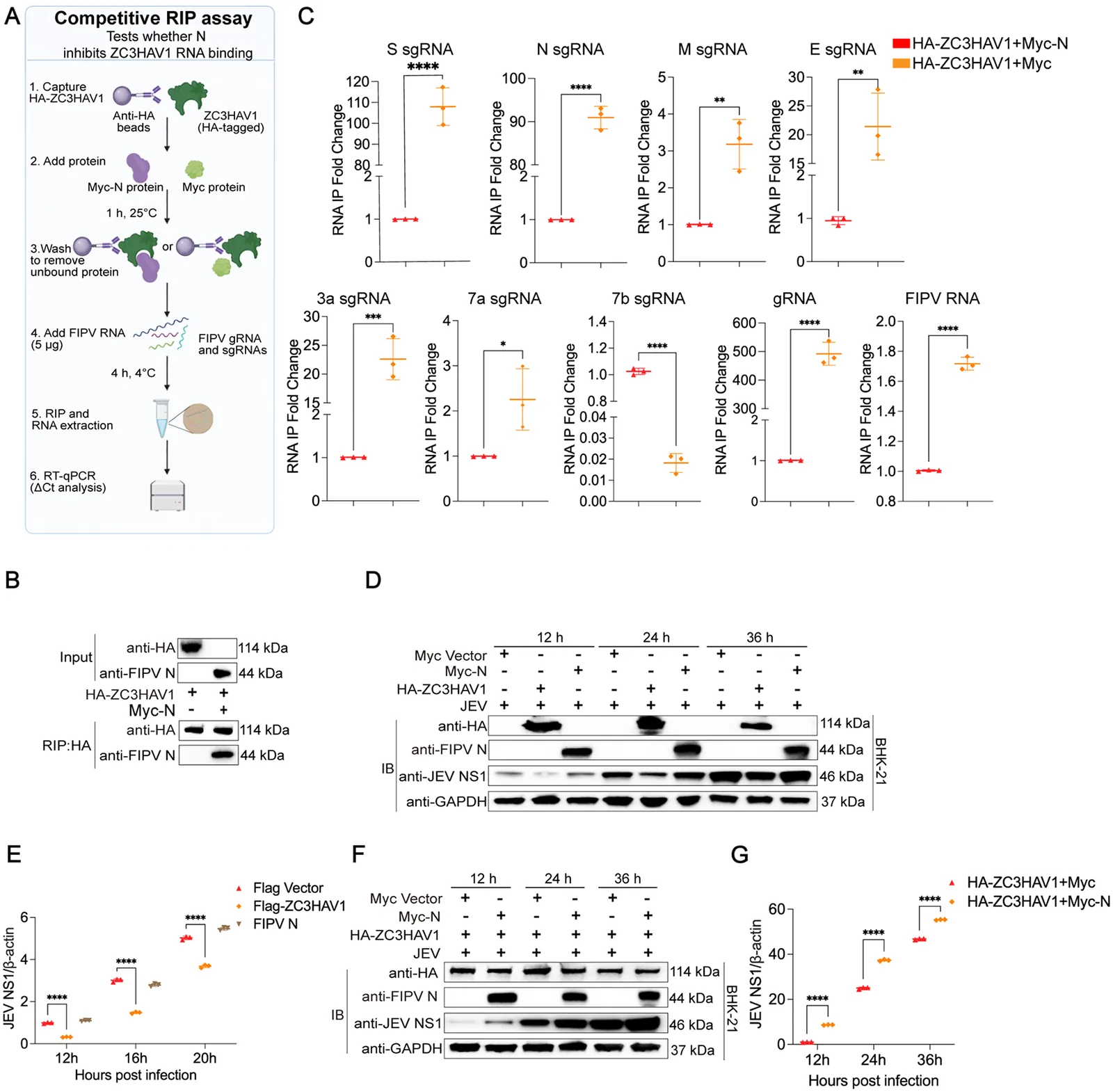
FIPV N disrupts ZC3HAV1–viral RNA association to attenuate ZC3HAV1-mediated RNA-level restriction. **(A)** Schematic diagram of the RIP-competition assay used to examine whether FIPV N affects ZC3HAV1-associated viral RNA recovery. HA-ZC3HAV1 was immobilized using anti-HA antibody-coupled Protein A/G beads and incubated with Myc control or Myc-N before addition of FIPV RNA. After washing and RNA capture, ZC3HAV1-associated viral RNAs were quantified by RT-qPCR. Created in BioRender. Li, **N.** (2026) https://BioRender.com/of6gq93. **(B)** Western blot verification of HA-ZC3HAV1 and Myc-N recovery in the RIP-competition assay. Input and anti-HA immunoprecipitated fractions were analyzed using anti-HA and anti-FIPV N antibodies. **(C)** RIP-qPCR analysis of FIPV genomic RNA (gRNA), total FIPV RNA, and the indicated subgenomic RNAs (sgRNAs) recovered with HA-ZC3HAV1 in the presence of Myc control or Myc-N. **(D)** Western blot analysis of JEV NS1 protein expression in BHK-21 cells transfected with Myc vector, HA-ZC3HAV1, or Myc-N, followed by JEV infection. HA-ZC3HAV1 was detected using anti-HA antibody, Myc-N was detected using anti-FIPV N antibody, JEV NS1 was detected using anti-JEV NS1 antibody, and GAPDH served as the loading control. **(E)** Densitometric quantification of JEV NS1 protein levels normalized to GAPDH from panel **D. (F)** Western blot analysis of JEV NS1 protein expression in BHK-21 cells co-expressing HA-ZC3HAV1 with Myc control or Myc-N, followed by JEV infection. **(G)** Densitometric quantification of JEV NS1 protein levels normalized to GAPDH from panel **F.** JEV was used as a heterologous positive-strand RNA virus model to evaluate whether FIPV N-mediated attenuation of ZC3HAV1-associated restriction could be observed outside the FIPV replication context. Data are mean ± SEM (n = 3). Statistics: *P < 0.05, **P < 0.01, ***P < 0.001, ****P < 0.0001; ns, not significant.

Having established that FIPV N interacts with ZC3HAV1 and that ZC3HAV1 associates with FIPV genomic and subgenomic RNAs, we next investigated whether N interferes with ZC3HAV1–viral RNA association. We first performed a competitive RIP assay in which HA-ZC3HAV1 was pre-bound to Protein A/G–coupled anti-HA beads and then incubated with Myc-tagged FIPV N or Myc control before RNA capture (Fig 6A). Under these conditions, N readily associated with bead-bound HA-ZC3HAV1 (Fig 6B), but its presence significantly reduced the recovery of multiple FIPV transcripts in the ZC3HAV1 RIP, including gRNA and several sgRNAs (Fig 6C). These results indicate that pre-associated FIPV N can impair the ability of ZC3HAV1 to capture FIPV genomic and subgenomic RNAs. However, because this assay mainly tested whether N bound to ZC3HAV1 before RNA exposure limits subsequent RNA recovery, it did not determine whether N can disrupt pre-formed ZC3HAV1–viral RNA complexes.

To address this issue, we next performed a displacement RIP assay using WT FIPV N and an RNA-binding-impaired N mutant. Mut-N was generated by substituting three conserved RNA-binding residues in FIPV N, Y93, R117, and K123, with alanine (Y93A/R117A/K123A) (S5A Fig.). Under RNase-treated conditions, Mut-N retained detectable interaction with HA-ZC3HAV1, comparable to WT N, indicating that these mutations did not abolish N–ZC3HAV1 association (S5B Fig). However, RIP-qPCR analysis showed that Mut-N displayed markedly reduced enrichment of FIPV gRNA and multiple sgRNAs compared with WT N, confirming that the mutant was impaired in viral RNA association while retaining ZC3HAV1-binding capacity (S5C Fig).

We then designed a displacement RIP assay to test whether FIPV N can reduce viral RNA association after ZC3HAV1–RNA complexes have already formed. In this assay, bead-bound HA-ZC3HAV1 was first incubated with FIPV RNA or host RNA to allow RNA binding, unbound RNA was removed by washing, and Myc-tagged WT N or Mut-N was subsequently added before RIP-qPCR analysis (S5D Fig). WT N markedly reduced the levels of ZC3HAV1-associated FIPV gRNA and multiple sgRNAs, whereas Mut-N showed substantially weaker displacement activity (S5E Fig). These results demonstrate that FIPV N can reduce ZC3HAV1-associated viral RNA after ZC3HAV1–viral RNA complexes have formed, and that efficient disruption requires the viral RNA-binding capacity of N.

To determine whether this effect reflected nonspecific disruption of ZC3HAV1-bound RNAs, we included HSPG2 mRNA as a representative ZC3HAV1-associated host RNA control. In contrast to the viral RNA results, neither WT N nor Mut-N significantly reduced ZC3HAV1 association with HSPG2 mRNA (S5F Fig). Although analysis of a single host transcript cannot exclude effects on all cellular RNAs, this result argues against a general nonspecific dissociation of ZC3HAV1–RNA complexes under our assay conditions. Together, the competitive RIP and displacement RIP experiments support a viral RNA-biased antagonism model in which FIPV N interferes with ZC3HAV1–viral RNA association through coordinated ZC3HAV1 interaction and viral RNA-binding activity.

To determine whether FIPV N-mediated attenuation of ZC3HAV1-associated restriction was strictly dependent on the FIPV replication context, we used Japanese encephalitis virus (JEV) as a heterologous positive-strand RNA virus model. JEV was selected because it replicates in the cytoplasm and provides an experimentally tractable RNA virus system in which ZC3HAV1-associated antiviral activity can be evaluated independently of FIPV replication complexes. In BHK-21 cells infected with JEV, ZC3HAV1 expression reduced JEV NS1 protein accumulation, whereas FIPV N expression was associated with increased NS1 protein levels relative to the ZC3HAV1-expressing condition (Fig 6D–E). When FIPV N was co-expressed with ZC3HAV1, JEV NS1 protein expression was partially restored compared with ZC3HAV1 expression alone (Fig 6F–G). These results indicate that FIPV N can attenuate ZC3HAV1-associated restriction in a heterologous RNA virus system at the level of viral protein accumulation, suggesting that this counter-restriction activity is not strictly limited to the FIPV replication context.

Overall, these results support a coordinated antagonism model in which FIPV N uses both protein–protein and protein–RNA interactions to counteract ZC3HAV1-associated RNA-level restriction. The N–ZC3HAV1 interaction likely positions N at the ZC3HAV1–viral RNA interface, whereas the intrinsic viral RNA-binding activity of N contributes to reducing ZC3HAV1-associated viral RNA. Thus, FIPV N does not appear to antagonize ZC3HAV1 solely by sterically blocking the ZC3HAV1 RBD-containing region; rather, it can disrupt pre-formed ZC3HAV1–viral RNA complexes in a manner that depends, at least in part, on N-mediated viral RNA binding. The HSPG2 control further supports that this effect is biased toward viral RNA rather than reflecting global disruption of ZC3HAV1-associated RNAs. The heterologous JEV assay provides additional evidence that FIPV N-mediated attenuation of ZC3HAV1-associated antiviral activity can be observed outside the FIPV infection system.

## Discussion

Coronaviruses have evolved intricate molecular strategies to evade host RNA surveillance and preserve viral RNA accumulation and persistence. These RNA surveillance and decay systems represent an essential first line of intracellular defense, capable of recognizing and degrading non-self RNAs before the activation of interferon signaling [23,24]. Among coronavirus structural proteins, the nucleocapsid (N) protein has been increasingly implicated in this process, functioning not only in RNA encapsidation and replication but also in modulating host antiviral pathways [25]. In the case of feline infectious peritonitis virus (FIPV), a coronavirus responsible for severe and often fatal systemic disease in cats, understanding how N circumvents host RNA restriction is particularly important, as efficient control of viral RNA synthesis and turnover is closely linked to the viral load dynamics and immunopathological outcome of feline infectious peritonitis (FIP). This study therefore used FIPV as a pathogenic model to dissect how coronavirus structural proteins antagonize host RNA-level defenses. Through proteomic screening and functional validation, we identified the host antiviral restriction factor ZC3HAV1 as a major host factor targeted by FIPV N, revealing a previously unrecognized post-transcriptional counter-restriction mechanism.

Proteomic profiling revealed that the FIPV N protein engages an extensive network of host RNA-binding and translational regulators, forming an interaction landscape partially overlapping with those described for SARS-CoV-2 [26] and mouse hepatitis virus (MHV) [27]. In these viruses, N has been shown to interact with ribosomal proteins and stress-granule components, reshaping host RNA regulatory environments to favor viral translation and RNA stability [28,29]. Functional validation confirmed that several of these N-associated host proteins—including ZC3HAV1 and MOV10, both known for their post-transcriptional antiviral roles [30,31]—restricted FIPV replication. Among these candidates, ZC3HAV1 was particularly notable because of its established role as an RNA-binding restriction factor, prompting us to further define how ZC3HAV1 restricts FIPV and how FIPV N counteracts this restriction.

Given its established role as an RNA-binding antiviral factor, we next examined the level at which ZC3HAV1 restricts FIPV replication. ZC3HAV1 reduced both genomic and subgenomic viral RNA accumulation, consistent with its previously reported restriction of SARS-CoV-2 [32]. Importantly, reduced steady-state gRNA and sgRNA levels measured by RT-qPCR alone cannot determine whether ZC3HAV1 acts by inhibiting viral RNA synthesis or by reducing the stability of already synthesized viral RNAs. To distinguish these possibilities, we performed a GS-441524-based RNA persistence assay. Because GS-441524 strongly inhibits coronavirus RNA-dependent RNA polymerase activity [33,34], treatment after viral RNAs had accumulated limited ongoing viral RNA synthesis, thereby enabling assessment of pre-existing FIPV RNA persistence. Under these conditions, ZC3HAV1 accelerated the loss of FIPV genomic and subgenomic RNAs, indicating that ZC3HAV1-mediated restriction is not primarily attributable to reduced viral RNA synthesis or sgRNA transcription. Rather, these data support a post-synthetic RNA destabilization mechanism in which ZC3HAV1 decreases the persistence of FIPV RNAs after they are produced. Although this finding is consistent with direct or cofactor-assisted destabilization of ZC3HAV1-bound viral RNAs, the specific RNA-decay pathway and host cofactors involved in FIPV RNA loss remain to be identified. This mechanistic refinement is important because it places ZC3HAV1 restriction at the level of viral RNA persistence and provides a direct rationale for why FIPV N, by disrupting ZC3HAV1–viral RNA association, can preserve viral RNA accumulation during infection.

Having established that ZC3HAV1 restricts FIPV replication at the RNA level, we next sought to elucidate how it recognizes viral transcripts. Remarkably, ZC3HAV1 efficiently restricted FIPV despite the virus’s low genomic CpG content, a feature shared by many coronaviruses, including SARS-CoV-2 [17,35]. In our CpG profiling analysis, FIPV showed broad CpG depletion across most of the genome, with only localized enrichment near the 5′ region. Further mapping of the 5′ UTR showed that this CpG enrichment did not coincide with the canonical leader–body junction shared by coronavirus sgRNAs. These observations suggest that global CpG frequency, and even local CpG distribution within the leader-containing region, is insufficient to fully explain ZC3HAV1-associated restriction of FIPV RNA. This interpretation is consistent with previous studies showing that ZC3HAV1-mediated restriction is not always strictly predicted by global CpG abundance and that ZC3HAV1 can restrict viruses with relatively low CpG frequencies or through context-dependent RNA recognition modes [36,37]. Therefore, rather than demonstrating complete CpG independence, our data support a noncanonical mode of ZC3HAV1 engagement in a CpG-suppressed coronavirus RNA context.

Our RIP assays further showed that full-length ZC3HAV1 and its RBD-containing region enriched both FIPV gRNA and multiple sgRNAs, whereas the WWE + PARP region showed minimal enrichment. These data indicate that the RBD-containing region is required for efficient association of ZC3HAV1 with FIPV RNAs in infected cells. Notably, several sgRNAs encoding structural proteins, including N and S, were strongly enriched, which may reflect their high intracellular abundance and/or RNA-context features that promote ZC3HAV1 engagement. Because RIP does not define the precise RNA motif or structure recognized by ZC3HAV1, additional features—including localized CpG-enriched regions, RNA abundance, secondary structure, local sequence context, or viral ribonucleoprotein organization—may contribute to ZC3HAV1 engagement of FIPV RNAs [15,38]. This provides a mechanistic framework for understanding how FIPV, despite overall CpG depletion, remains sensitive to ZC3HAV1-associated RNA-level restriction.

The localized CpG enrichment near the FIPV 5′ region may also contribute to ZC3HAV1 engagement. Although this enriched region does not overlap precisely with the canonical leader–body junction, it could influence ZC3HAV1 recognition through local RNA structure, shared 5′ RNA architecture, or viral ribonucleoprotein organization. Thus, our data do not exclude a role for the 5′ CpG-enriched region, but rather indicate that FIPV RNA recognition is not explained by global CpG frequency alone or by a simple leader-junction CpG model. Targeted mutational mapping of the 5′ CpG-enriched region, leader-containing reporter RNAs, or FIPV minigenome/replicon systems will be useful to determine whether this region directly contributes to ZC3HAV1 binding and restriction.

These observations also suggest that ZC3HAV1 recognition during FIPV infection is unlikely to be restricted to a single viral RNA species or dictated solely by a simple global CpG threshold. The ability of FIPV N to attenuate ZC3HAV1-mediated restriction in a heterologous JEV system, together with previous observations for PEDV N in an influenza virus system [18], supports the view that ZC3HAV1 is capable of engaging viral RNAs in different experimental contexts. In this context, our RIP-qPCR data identify FIPV gRNA and sgRNAs as relevant ZC3HAV1-associated viral RNA species during FIPV infection. Importantly, HSPG2 was included as a representative ZC3HAV1-associated non-viral RNA control in the displacement RIP assay. The finding that FIPV N efficiently reduced ZC3HAV1-associated FIPV RNAs but did not significantly reduce ZC3HAV1 association with HSPG2 suggests that N-mediated displacement is not a general nonspecific disruption of all ZC3HAV1–RNA complexes. Instead, this result supports a viral RNA-biased antagonism model in which FIPV N preferentially interferes with ZC3HAV1 association with viral RNAs, likely through coordinated N–ZC3HAV1 interaction and N-mediated viral RNA binding. Nevertheless, transcriptome-wide approaches such as ZC3HAV1 RIP-seq, CLIP-seq, or related RNA interactome analyses will be required to define the full range of viral RNA substrates and binding features recognized by ZC3HAV1 during coronavirus infection [38].

**Fig 7:**
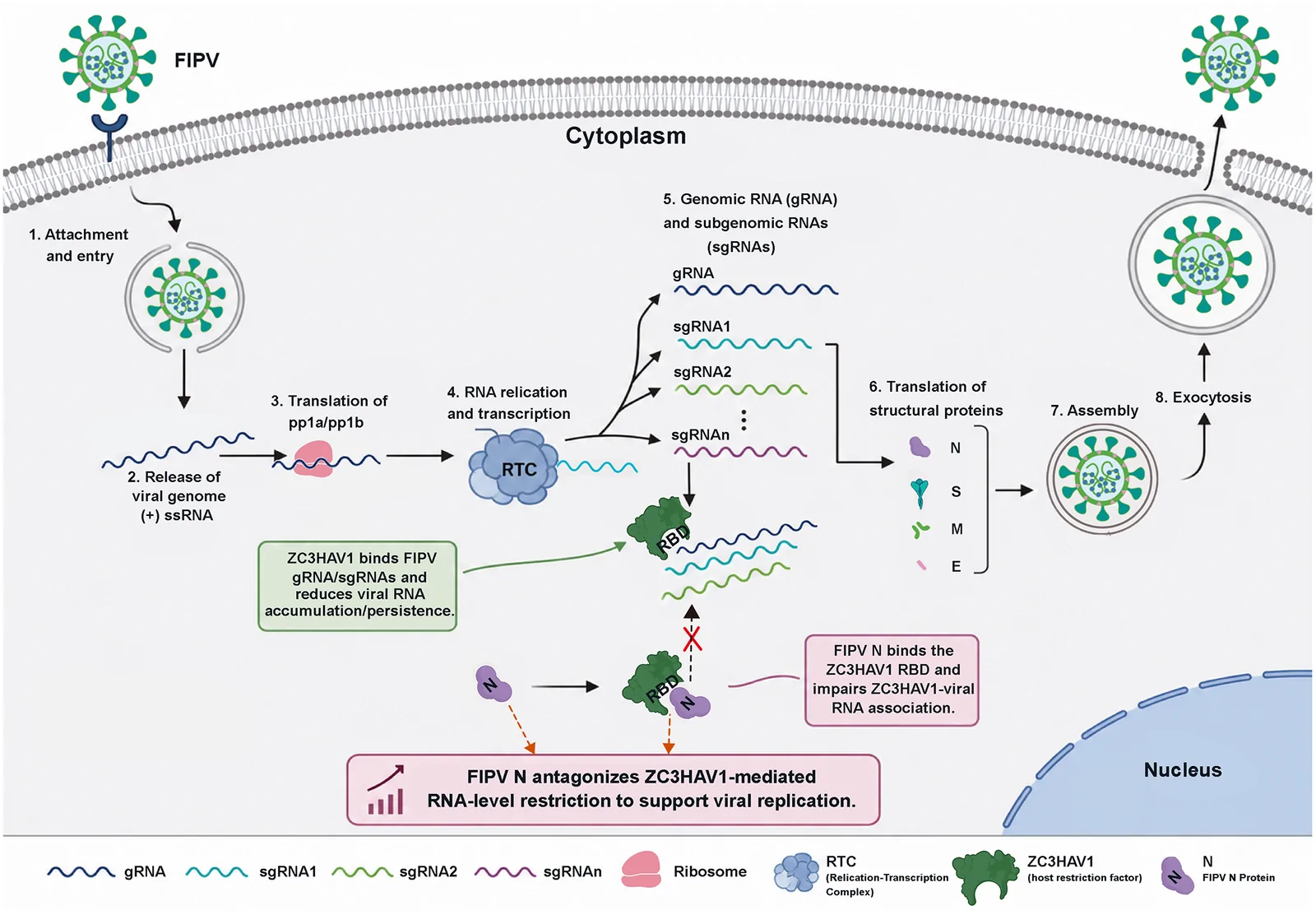
Proposed model of FIPV N-mediated counteraction of ZC3HAV1-associated RNA-level restriction. ZC3HAV1 associates with FIPV genomic and subgenomic RNAs through its RBD-containing region and reduces viral RNA accumulation and persistence. FIPV N interacts with the RBD-containing region of ZC3HAV1 and disrupts ZC3HAV1–viral RNA association through coordinated protein–protein and protein–RNA interactions, thereby helping preserve viral RNA persistence during infection. This counter-restriction mechanism supports sustained viral RNA accumulation and productive FIPV replication. Created in BioRender. Li, **N.** (2026) https://BioRender.com/j2tfld0.

Our data further refine the mechanism by which FIPV N counteracts ZC3HAV1-mediated restriction. A simple competition model, in which N simply masks the ZC3HAV1 RNA-binding region and prevents viral RNA access, is insufficient to explain the displacement RIP results. In the redesigned assay, WT N reduced ZC3HAV1-associated FIPV RNAs after ZC3HAV1–viral RNA complexes had already formed, whereas the RNA-binding-impaired Mut-N retained detectable ZC3HAV1 interaction but showed markedly weaker displacement activity. These results indicate that N-mediated antagonism requires more than protein–protein association with ZC3HAV1. Instead, FIPV N likely acts through a coordinated mechanism in which its interaction with ZC3HAV1 positions N at the ZC3HAV1–viral RNA interface, while its intrinsic viral RNA-binding activity contributes to reducing ZC3HAV1-associated viral RNA. This model also explains why disruption of ZC3HAV1–viral RNA association, rather than degradation of ZC3HAV1 itself, represents a major antagonistic mechanism mediated by FIPV N. Mechanistically, this antagonism expands the known spectrum of viral countermeasures against ZC3HAV1. We show that the FIPV N protein associates with the RBD-containing region of ZC3HAV1 and impairs its association with viral RNA. Together with the displacement RIP and Mut-N analyses, these findings support a coordinated antagonistic mechanism in which FIPV N uses both ZC3HAV1 association and intrinsic viral RNA-binding activity to reduce ZC3HAV1-associated viral RNA recovery. This mechanism differs from previously described antagonists such as influenza A virus NS1, which suppresses ZC3HAV1/ZAP-mediated RNA-level restriction indirectly by disrupting cofactor-dependent pathways [39], and vaccinia virus C16, which sequesters ZC3HAV1/ZAP into cytoplasmic puncta to attenuate its antiviral activity [40]. To our knowledge, these findings provide evidence that a coronavirus structural protein can counteract ZC3HAV1-associated RNA restriction by targeting the ZC3HAV1–viral RNA axis, rather than through proteolytic or transcriptional suppression of ZC3HAV1. Through these coupled activities, N may function not only as an RNA chaperone but also as a viral antagonist that helps preserve viral RNA accumulation and persistence under ZC3HAV1-mediated restriction. This mechanism identifies a previously underappreciated RNA-level counter-restriction strategy by which FIPV N helps sustain viral RNA accumulation during infection (Fig 7).

A remaining question is which downstream host cofactors execute ZC3HAV1-associated restriction of FIPV RNAs. In other viral systems, ZC3HAV1 has been reported to cooperate with RNA-surveillance or decay-associated factors, including TRIM25, KHNYN, OAS3, and related host proteins, to promote antiviral RNA restriction [41–43]. Although these cofactors provide important candidates for future investigation, the present study focuses on defining the upstream virus–host interface that enables ZC3HAV1-mediated restriction and FIPV N-mediated counter-restriction. Specifically, ZC3HAV1 associates with FIPV genomic and subgenomic RNAs, reduces their accumulation and persistence, and FIPV N counteracts this process by disrupting ZC3HAV1–viral RNA association. By combining RNA persistence assays, RIP-qPCR, and displacement RIP analyses, our study moves beyond steady-state viral RNA quantification and links ZC3HAV1-associated RNA restriction to a specific viral antagonism mechanism. Further mechanistic resolution will require targeted depletion or rescue of candidate cofactors, broader interactome analyses, and RNA-centered approaches such as FIPV replicon systems, transcription-defective mutants, metabolic RNA labeling, or Northern blotting. These approaches would help determine whether canonical ZC3HAV1 cofactors or FIPV-specific RNA-decay pathways execute viral RNA loss and would further clarify the relative contributions of viral RNA synthesis, processing, and decay to ZC3HAV1-mediated restriction.

To further test whether FIPV N-mediated attenuation of ZC3HAV1-associated restriction was strictly confined to the FIPV replication context, we used JEV as a heterologous positive-strand RNA virus model. The ability of FIPV N to partially alleviate ZC3HAV1-mediated restriction of JEV suggests that this counter-restriction activity can be observed outside the FIPV infection system. This finding supports the functional transferability of FIPV N-mediated ZC3HAV1 antagonism and indicates that the activity is not entirely dependent on FIPV-specific replication machinery. However, additional coronaviruses and unrelated RNA viruses will be needed to define the breadth of viral RNA contexts in which FIPV N can counteract ZC3HAV1-associated restriction.

Collectively, these findings suggest that the FIPV N protein has an additional RNA-protective role beyond its established functions in viral RNA packaging, replication support, and immune modulation [44,45]. This work links the classical RNA-binding function of coronavirus N proteins to the evasion of cellular RNA-level restriction and identifies N-mediated disruption of the ZC3HAV1–viral RNA interface as a previously underappreciated mode of coronavirus counter-restriction.

## Conclusions

In summary, this study uses a clinically fatal feline coronavirus as a model to reveal an RNA-level counter-restriction mechanism in which the nucleocapsid protein engages ZC3HAV1 through its interaction with the RNA-binding domain-containing region and functionally counteracts ZC3HAV1-mediated antiviral restriction. Functional analysis of N truncation mutants further indicates that the ZC3HAV1-associated region of FIPV N contributes to efficient antagonism of ZC3HAV1-mediated restriction. In addition, FIPV N interferes with ZC3HAV1–viral RNA association, thereby helping preserve viral RNA accumulation and persistence. These findings provide a mechanistic explanation for how FIPV helps sustain abundant genomic and subgenomic RNAs despite the presence of a host RNA-binding restriction factor, and extend the functional role of coronavirus N protein from viral RNA packaging and replication support to active modulation of host RNA-level restriction. Importantly, our data support a model in which ZC3HAV1-mediated restriction acts, at least in part, at a post-synthetic stage of the viral RNA life cycle, limiting the persistence of already synthesized FIPV RNAs rather than being explained solely by reduced viral RNA synthesis or altered sgRNA transcription. Although the precise downstream RNA-decay machinery remains to be defined, the coordinated targeting of the ZC3HAV1–viral RNA restriction axis by FIPV N represents a specific host–virus interface that may provide a conceptual basis for future antiviral strategies aimed at restoring intrinsic RNA restriction and limiting coronavirus RNA persistence.

## Supporting information

S1 Fig FIPV N does not directly reduce ZC3HAV1 protein abundance, and FIPV infection induces endogenous ZC3HAV1 expression. (A) Western blot analysis of HA-ZC3HAV1 protein levels in CRFK cells co-transfected with HA-ZC3HAV1 and increasing amounts of Flag-FIPV N plasmid. Flag vector was used as the control. HA-ZC3HAV1 was detected using anti-HA antibody, Flag-FIPV N was detected using anti-Flag antibody, and β-actin served as the loading control. (B) Densitometric quantification of HA-ZC3HAV1 protein levels shown in panel A, normalized to β-actin. (C) Western blot analysis of endogenous ZC3HAV1 protein expression in mock-infected and FIPV-infected CRFK cells at 12, 16, and 24 h post-infection. Endogenous ZC3HAV1 was detected using anti-ZC3HAV1 antibody, FIPV N was detected using anti-FIPV N antibody as a marker of productive viral infection, and β-actin served as the loading control. (D) Densitometric quantification of endogenous ZC3HAV1 protein levels shown in panel C, normalized to β-actin. Data are presented as mean ± SEM (n = 3). Statistical significance: **P < 0.01; ns, not significant.
(TIF)

S2 Fig ZC3HAV1 reduces the persistence of FIPV genomic and subgenomic RNAs under GS-441524-mediated replication suppression. (A) Time-course absolute RT-qPCR analysis of FIPV N RNA copies in FIPV-infected CRFK cells treated with or without GS-441524. GS-441524 treatment markedly suppressed the continued accumulation of FIPV RNA. (B) Time-course analysis of normalized FIPV sgRNA/gRNA expression in FIPV-infected CRFK cells treated with or without GS-441524. (C) RT-qPCR analysis of FIPV gRNA levels in GS-441524-treated cells transfected with HA vector or HA-ZC3HAV1, or with control siRNA (siNC) or siZC3HAV1. Viral RNA levels were normalized to GAPDH. (D) RT-qPCR analysis of FIPV sgRNA levels in GS-441524-treated cells transfected with HA vector or HA-ZC3HAV1. The indicated sgRNAs include S, N, M, E, 3a, 3c, 7a, and 7b. Viral RNA levels were normalized to GAPDH. (E) RT-qPCR analysis of FIPV sgRNA levels in GS-441524-treated cells transfected with siNC or siZC3HAV1. Viral RNA levels were normalized to GAPDH. (F) Relative sgRNA/gRNA ratios in GS-441524-treated cells transfected with HA vector or HA-ZC3HAV1. (G) Relative sgRNA/gRNA ratios in GS-441524-treated cells transfected with siNC or siZC3HAV1. Data are presented as mean ± SEM (n = 3). Statistical significance: *P < 0.05, **P < 0.01, ***P < 0.001, ****P < 0.0001; ns, not significant.
(TIF)

S3 Fig Immunofluorescence analysis of endogenous ZC3HAV1 and infection-derived FIPV N in CRFK cells. Mock-infected CRFK cells were included as a negative control for FIPV N staining. Endogenous ZC3HAV1 was detected in the AF594 channel, FIPV N was detected in the AF488 channel, and nuclei were stained with DAPI. Upon FIPV infection, infection-derived FIPV N accumulated mainly in the cytoplasm and showed partial overlap with endogenous ZC3HAV1-positive signals. Magnified images show the boxed regions.
(TIF)

S4 Fig Rescue analysis of ZC3HAV1-mediated restriction and functional evaluation of FIPV N truncation mutants in ZC3HAV1-depleted CRFK-shZC3HAV1 cells. (A) Western blot analysis of FIPV M protein expression in ZC3HAV1-depleted CRFK-shZC3HAV1 cells reconstituted with HA vector, full-length HA-ZC3HAV1, or HA-Z5, with Myc-N co-expressed in the full-length HA-ZC3HAV1 group where indicated, followed by FIPV infection. Cells were collected at 12 and 24 h post-infection. HA-tagged proteins were detected using anti-HA antibody, Myc-N was detected using anti-Myc antibody, FIPV M was detected using anti-FIPV M antibody, and β-actin served as the loading control. HA-Z5 represents a ZC3HAV1 truncation lacking the RBD-containing region involved in FIPV N interaction. (B) Densitometric quantification of FIPV M/β-actin levels from panel A. (C) TCID₅₀ assay measuring infectious FIPV titers in the indicated treatment groups at different time points post-infection. (D) Expression and functional analysis of full-length FIPV N and N truncation mutants in the ZC3HAV1 rescue system. ZC3HAV1-depleted CRFK-shZC3HAV1 cells were transfected with empty vector or HA-ZC3HAV1, followed by co-expression of full-length FIPV N or individual N truncation mutants (N1–N5) where indicated. Cells were subsequently infected with FIPV, and viral replication was evaluated by western blot analysis of FIPV M protein. HA-ZC3HAV1 was detected using anti-HA antibody, N constructs were detected using anti-flag antibody, FIPV M was detected using anti-FIPV M antibody, and β-actin served as the loading control. (E) Densitometric quantification of FIPV M/β-actin levels from panel D. (F) RT-qPCR analysis of FIPV RNA levels in the indicated treatment groups. (G) TCID₅₀ assay measuring infectious FIPV titers in the indicated treatment groups. Data are presented as mean ± SEM (n = 3). Statistical significance: **P < 0.01, ***P < 0.001, ****P < 0.0001; ns, not significant.
(TIF)

S5 Fig FIPV N disrupts pre-formed ZC3HAV1–viral RNA complexes in an RNA-binding-dependent manner. (A) Sequence alignment of WT FIPV N and the RNA-binding-impaired FIPV N mutant Mut-N. The substituted residues Y93A, R117A, and K123A are highlighted. (B) Co-immunoprecipitation analysis of HA-ZC3HAV1 with Myc-tagged WT N or Mut-N under RNase-treated conditions. HA-ZC3HAV1 was immunoprecipitated using anti-HA antibody, and input and immunoprecipitated proteins were detected using anti-HA and anti-Myc antibodies. (C) RIP-qPCR analysis of FIPV genomic RNA (gRNA) and indicated subgenomic RNAs (sgRNAs) enriched by WT N or Mut-N, showing reduced viral RNA association by Mut-N compared with WT N. (D) Schematic diagram of the displacement RIP assay. Flag-ZC3HAV1 was first incubated with FIPV RNA or host RNA to allow pre-formed ZC3HAV1–RNA complex formation, followed by addition of WT N or Mut-N before anti-Flag immunoprecipitation and RT-qPCR analysis. Created in BioRender. Li, N. (2026) https://BioRender.com/652dje3. (E) RIP-qPCR analysis of FIPV gRNA and indicated sgRNAs remaining associated with Flag-ZC3HAV1 after addition of WT N or Mut-N in the displacement RIP assay. (F) RIP-qPCR analysis of HSPG2 mRNA, a representative ZC3HAV1-associated host transcript, under the same displacement RIP conditions. Data are presented as mean ± SEM (n = 3). Statistical significance: **P < 0.01, ***P < 0.001, ****P < 0.0001; ns, not significant.
(TIF)

S6 Fig Graphical Abstract. Schematic model of FIPV N-mediated antagonism of ZC3HAV1-associated RNA-level restriction. This graphical abstract illustrates how the FIPV nucleocapsid (N) protein counteracts ZC3HAV1-mediated RNA-level restriction during the FIPV life cycle. After receptor-mediated entry, the FIPV genomic RNA is released into the cytoplasm, translated to produce viral replicase proteins, and subsequently replicated and discontinuously transcribed into genomic RNA (gRNA) and multiple subgenomic RNAs (sgRNAs). ZC3HAV1 associates with FIPV gRNA and sgRNAs through its RBD-containing region, thereby reducing viral RNA accumulation and persistence. The FIPV N protein directly interacts with the RBD-containing region of ZC3HAV1 and impairs the association between ZC3HAV1 and viral RNAs. Through this coordinated protein–protein and protein–RNA interference mechanism, FIPV N attenuates ZC3HAV1-associated RNA-level restriction and helps preserve viral RNA accumulation and persistence. This counter-restriction activity thereby supports viral RNA accumulation, structural protein production, and progeny virus generation. This model highlights a counter-restriction mechanism by which the FIPV N protein interferes with the ZC3HAV1–viral RNA axis to facilitate viral replication, with potential relevance to other coronaviruses. Created in BioRender. Li, N. (2026) https://BioRender.com/j2tfld0.
(TIF)

S1 Raw Images Original uncropped and unadjusted blot and gel images. his file contains the original uncropped and unadjusted images underlying all blot and gel results reported in the main figures and supporting figures. The corresponding figure panels, detected targets, and molecular weight markers are indicated where applicable.
(PDF)

S1 Table Primers used for plasmid construction. This table lists the primer sequences used for homologous recombination-based plasmid construction, including primers for FIPV N expression constructs, ZC3HAV1 full-length and truncation constructs, selected host protein expression constructs, FIPV N truncation and mutant constructs, and the pLKO-shZC3HAV1 construct. Primer sequences are shown in the 5′–3′ orientation.
(DOCX)

S2 Table Primers used for RT-qPCR analysis of host gene transcription. This table lists the primer sequences used for RT-qPCR detection of host gene mRNA levels in CRFK cells after FIPV infection or related treatments. GAPDH was used as the internal reference gene. Primer sequences are shown in the 5′–3′ orientation.
(DOCX)

S3 Table siRNA sequences targeting ZC3HAV1. This table lists the sequences of three siRNAs designed to knock down endogenous ZC3HAV1 expression in CRFK cells. Sense and antisense sequences are shown for each siRNA in the 5′–3′ orientation.
(DOCX)

S4 Table Primers used to establish qPCR assays for FIPV gRNA and sgRNAs. This table lists the primer sequences used to identify the FIPV 5′ UTR leader sequence and to establish qPCR assays for FIPV genomic RNA (gRNA) and subgenomic RNAs (sgRNAs). Leader-region primers were used to map the 5′ UTR leader sequence, sgRNA-specific reverse primers were designed within the 5′ regions of the corresponding ORFs, and gRNA-specific primers were designed to avoid amplification of sgRNAs. Primer sequences are shown in the 5′–3′ orientation.
(DOCX)

S5 Table Complete processed IP-MS dataset for identification of FIPV N-associated host proteins. This table provides the processed peptide-group-level label-free LC–MS dataset used to identify host proteins associated with FIPV N in infection-based and transfection-based IP-MS analyses. The dataset includes peptide sequences, modification information, protein accessions, peptide-related parameters, Mascot search scores, and grouped abundance values for the CRFK, CRFK + FIPV, pCMV14-GFP, and pCMV14-FIPV N groups. These data were used to evaluate protein identification profiles, calculate enrichment relative to the corresponding background controls, and generate the final list of high-confidence FIPV N-associated host proteins for downstream functional analysis.
(XLSX)

S6 Table Functional overview of 18 representative FIPV N-associated host proteins selected for validation. This table summarizes 18 representative host proteins selected from the FIPV N-associated IP-MS dataset for subsequent validation. The table includes UniProt accession numbers, protein names, annotated functions, and LC–MS peptide coverage values. These candidates were prioritized based on their annotated roles in translation, RNA binding, RNA metabolism, cellular metabolism, or antiviral defense.
(DOCX)
