# Supplementary material for: A feline coronavirus nucleocapsid protein disrupts ZC3HAV1–viral RNA association to counteract host RNA-level restriction
Source: PLoS Pathog. 2026 Sep 18;22(9):e1014616. doi: 10.1371/journal.ppat.1014616 (PMC13600618; doi:10.1371/journal.ppat.1014616)
Supplement: Supplementary file 1 [file ppat.1014616.s007.pdf]

FIG 1B (left upper panel)

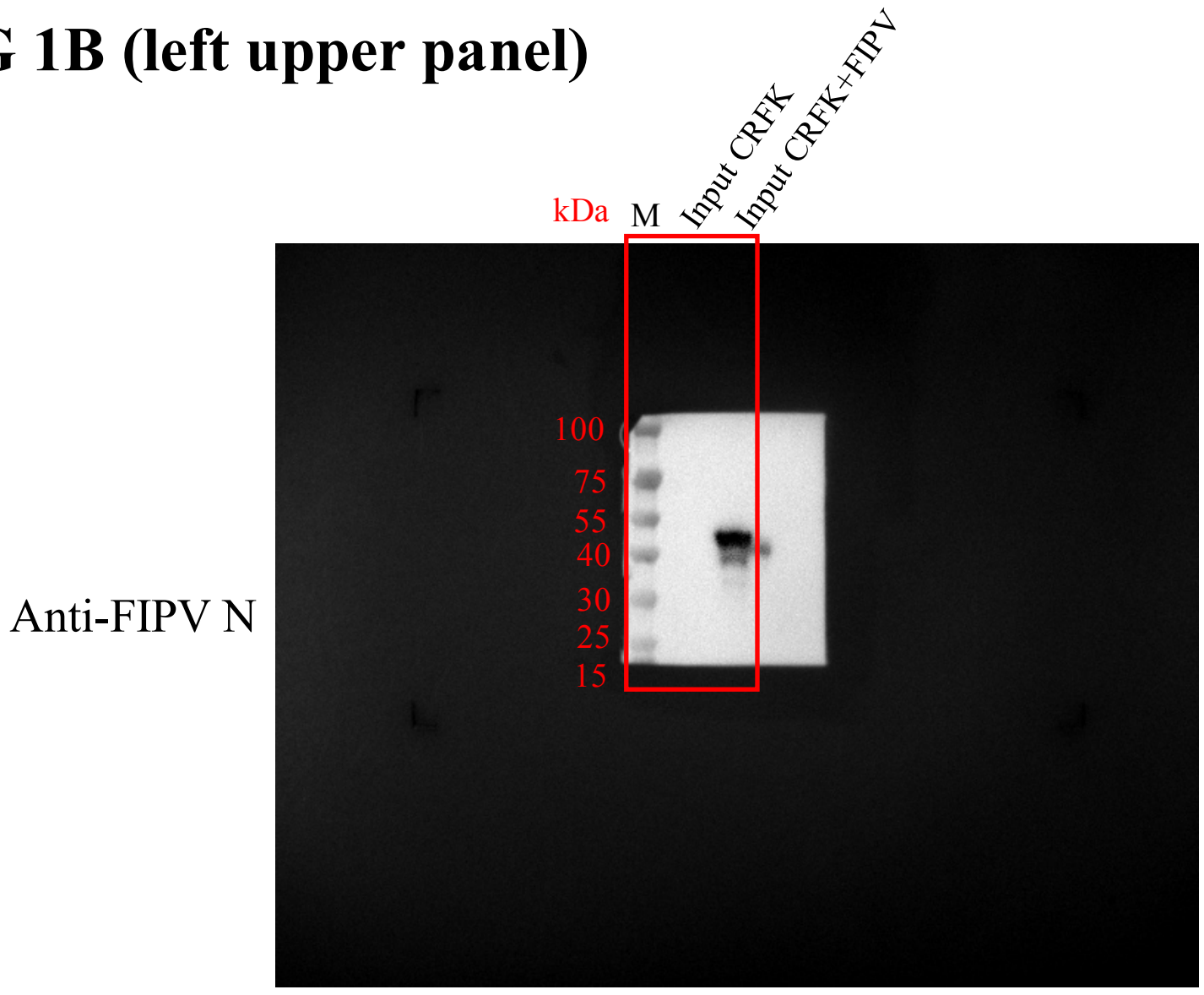

FIG 1B (left lower panel)

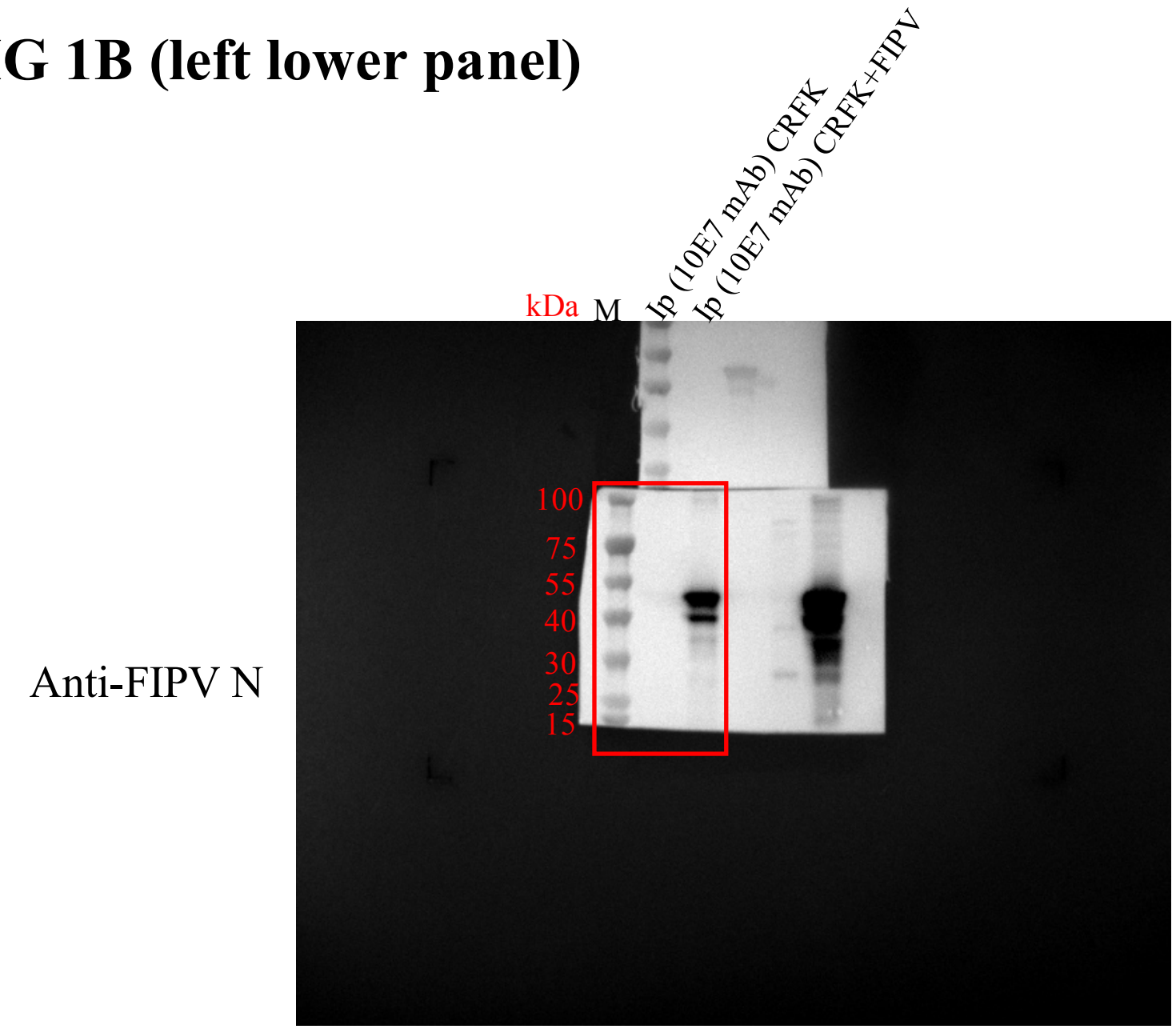

FIG 1B (right upper panel)

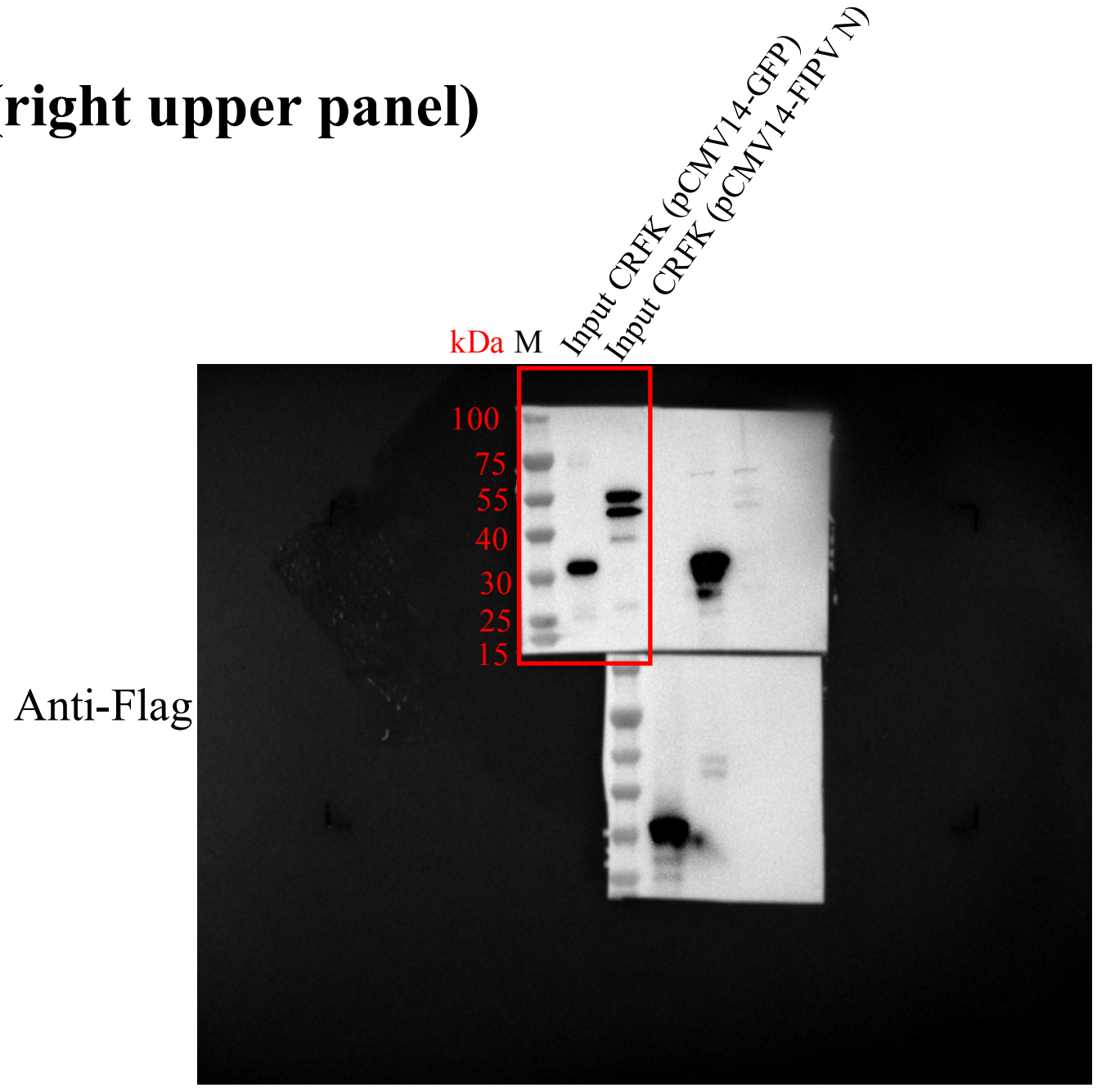

FIG 1B (right lower panel)

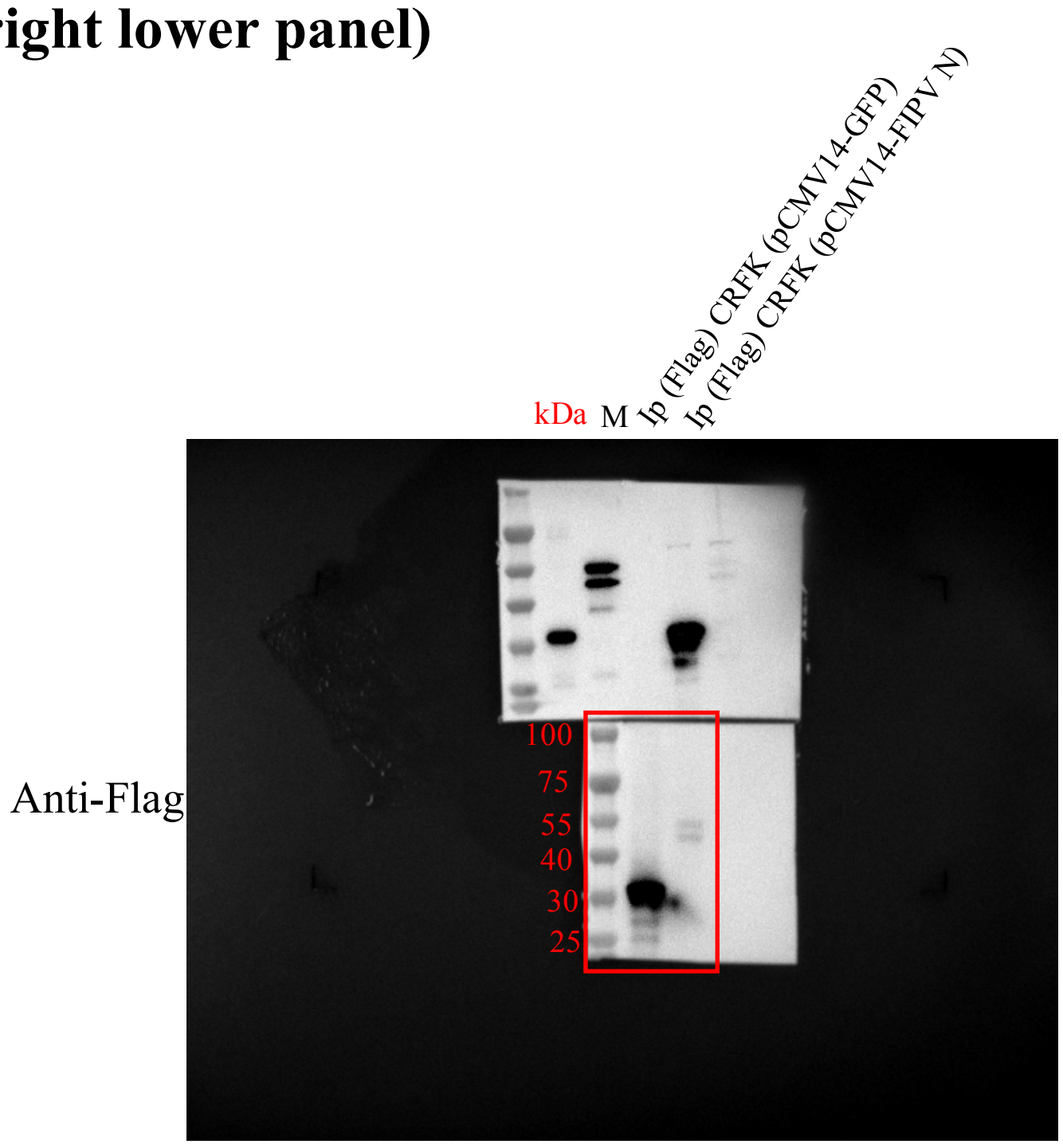

FIG 2A (panel 1)

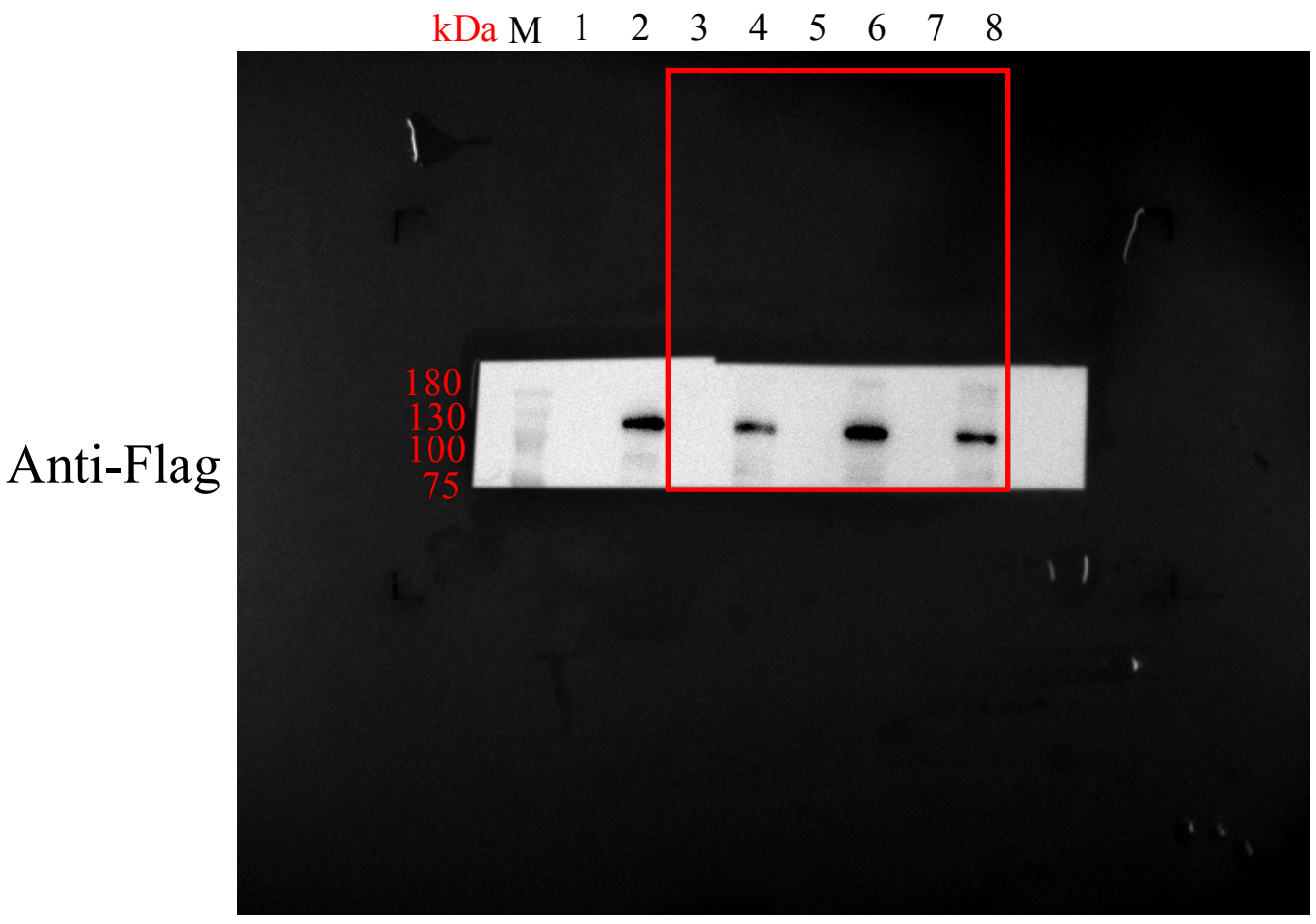

1: CRFK 8 hpi (Mock)
2: CRFK +FIPV 8 hpi
3: CRFK 12 hpi (Mock)
4: CRFK +FIPV 12 hpi
5: CRFK 16 hpi (Mock)
6: CRFK +FIPV 16 hpi
7: CRFK 24 hpi (Mock)
8: CRFK +FIPV 24 hpi

FIG 2A (panel 2)

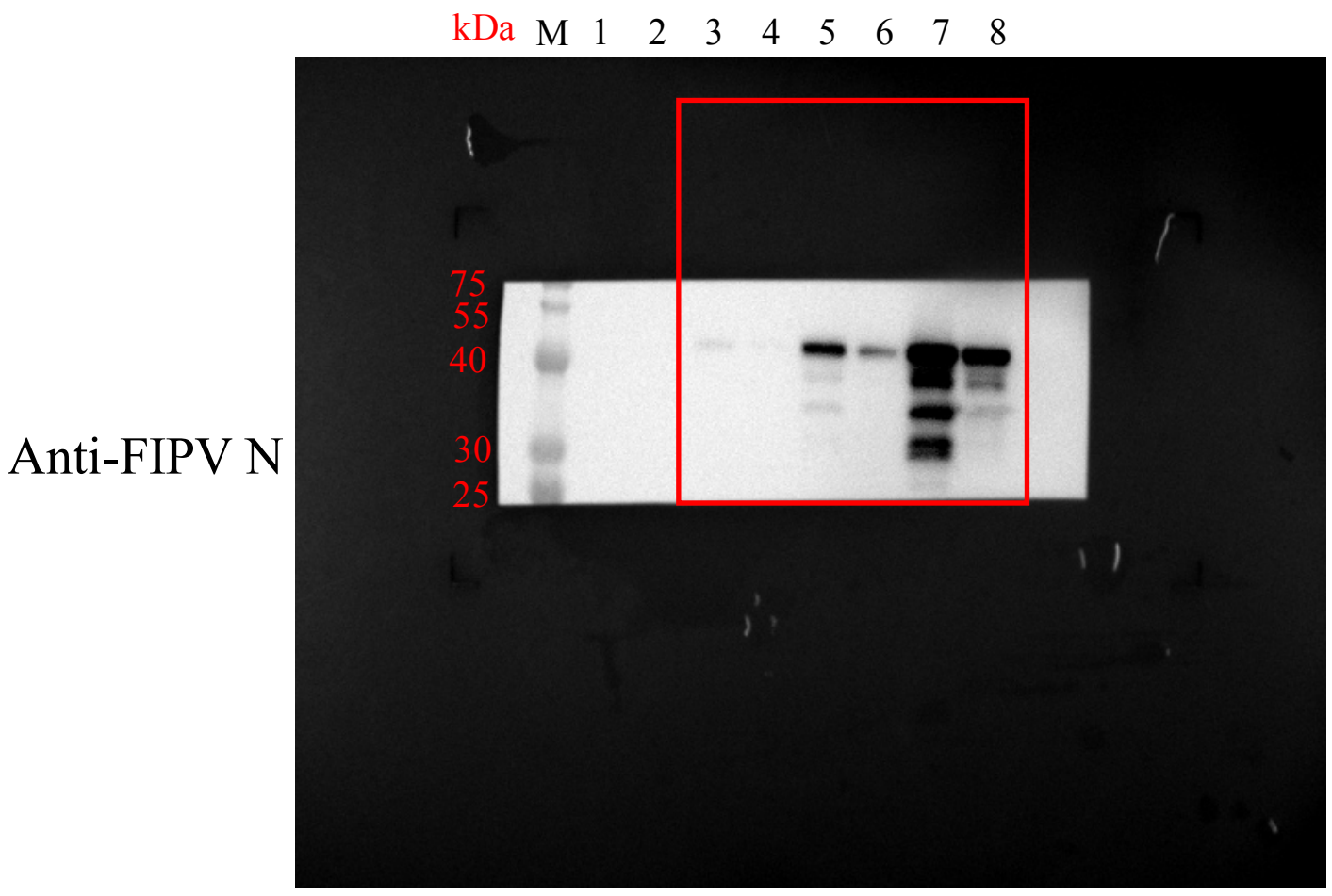

1: CRFK 8 hpi (Mock)
2: CRFK +FIPV 8 hpi
3: CRFK 12 hpi (Mock)
4: CRFK +FIPV 12 hpi
5: CRFK 16 hpi (Mock)
6: CRFK +FIPV 16 hpi
7: CRFK 24 hpi (Mock)
8: CRFK +FIPV 24 hpi

FIG 2A (panel 3)

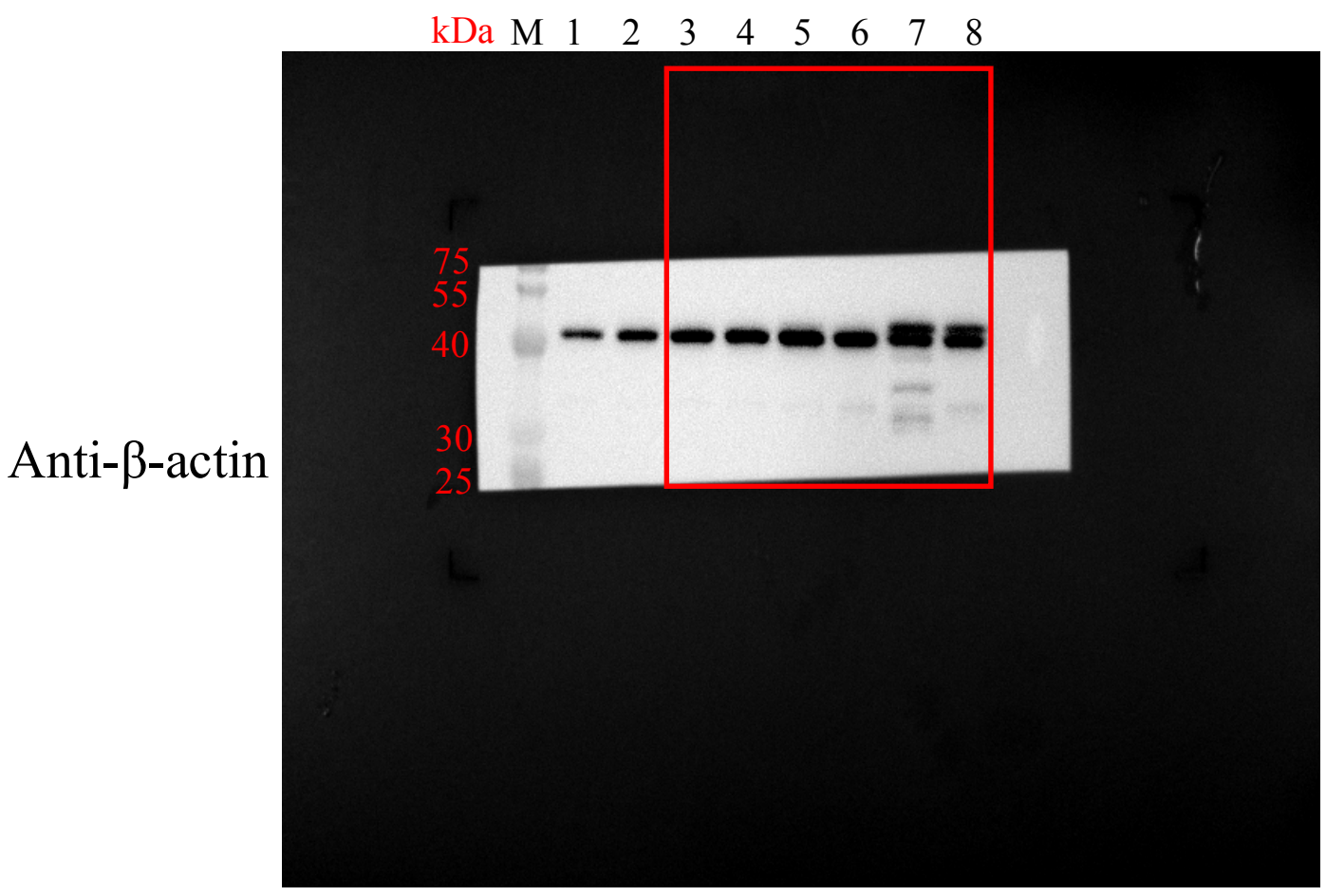

1: CRFK 8 hpi (Mock)
2: CRFK +FIPV 8 hpi
3: CRFK 12 hpi (Mock)
4: CRFK +FIPV 12 hpi
5: CRFK 16 hpi (Mock)
6: CRFK +FIPV 16 hpi
7: CRFK 24 hpi (Mock)
8: CRFK +FIPV 24 hpi

FIG 2C (panel 1)

Anti-Flag

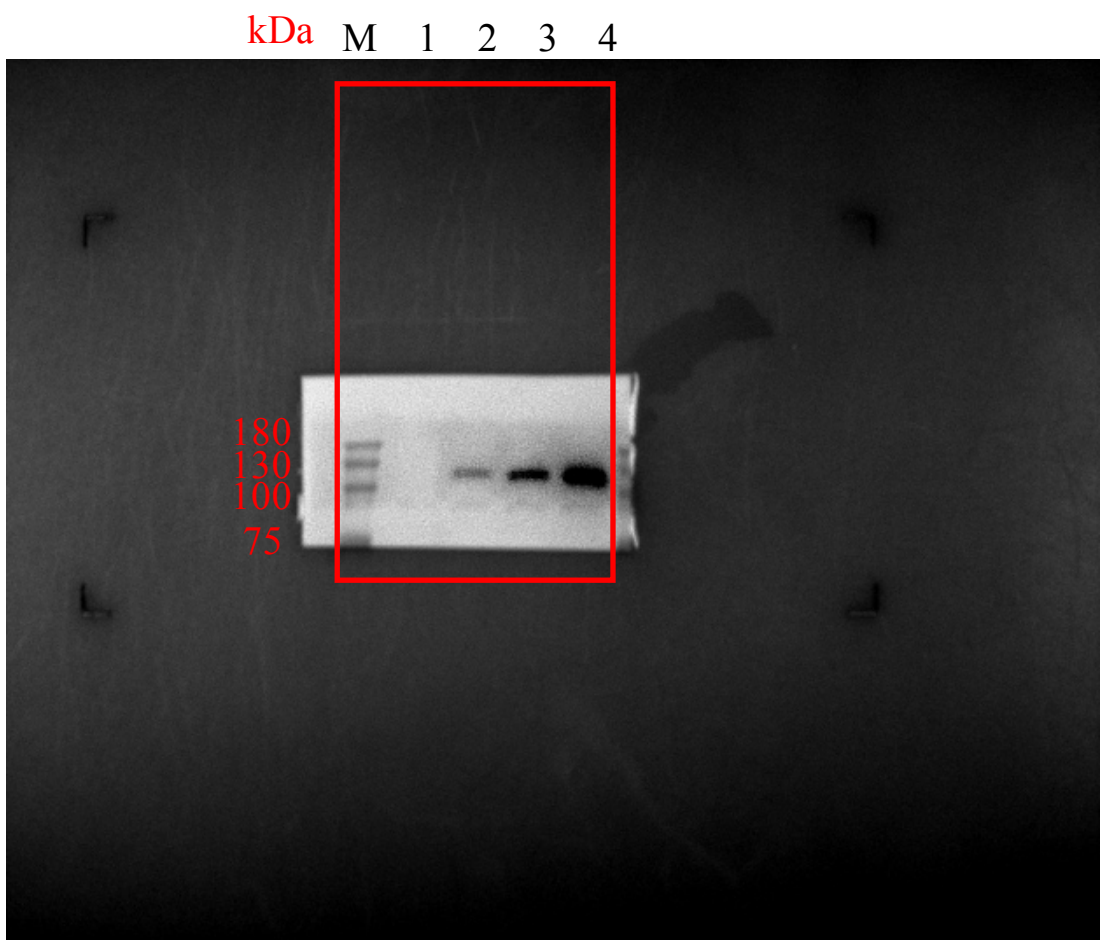

1: CRFK +pCAGGS-Flag (Mock)
2: CRFK +0.25 μg pCAGGS-ZC3HAV1
3: CRFK +0.5 μg pCAGGS-ZC3HAV1
4: CRFK +1 μg pCAGGS-ZC3HAV1

FIG 2C (panel 2)

Anti-FIPV N

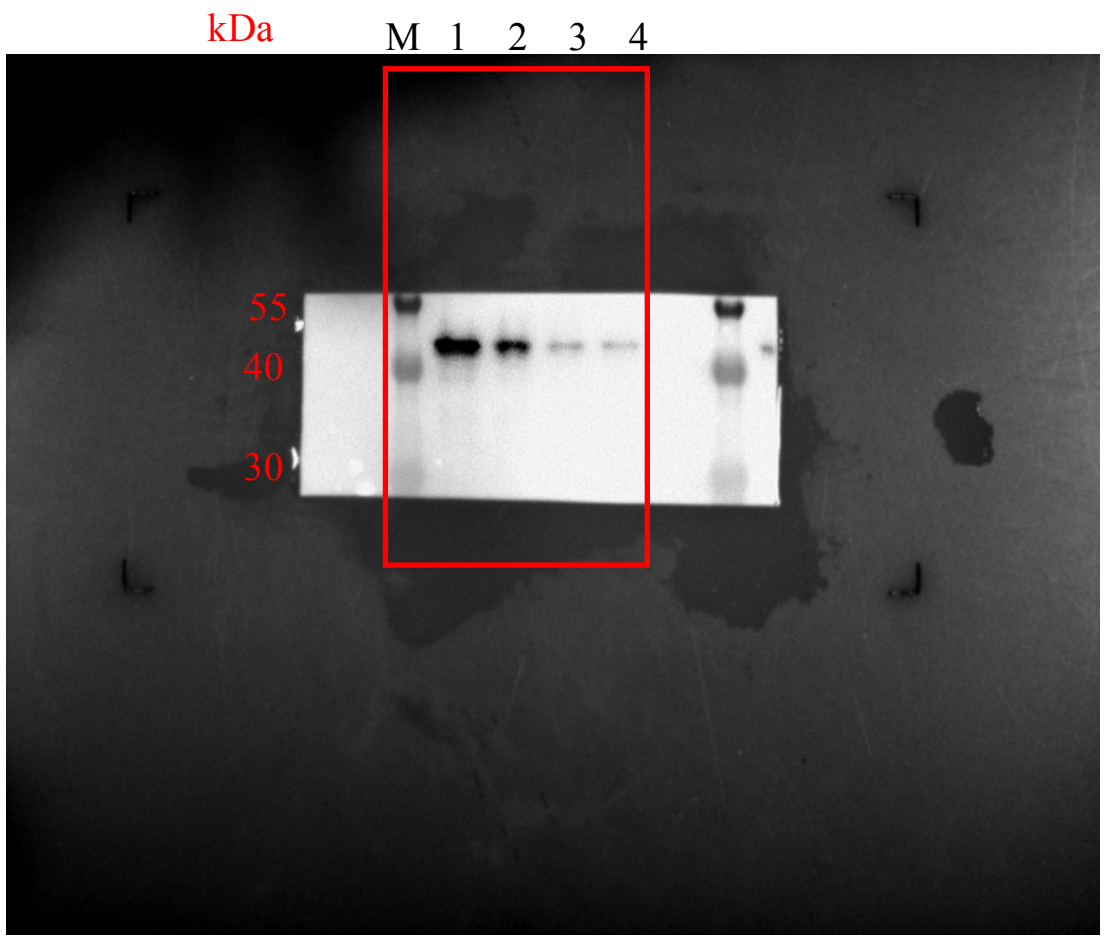

1: CRFK +pCAGGS-Flag (Mock)
2: CRFK +0.25 μg pCAGGS-ZC3HAV1
3: CRFK +0.5 μg pCAGGS-ZC3HAV1
4: CRFK +1 μg pCAGGS-ZC3HAV1

FIG 2C (panel 3)

Anti-β-actin

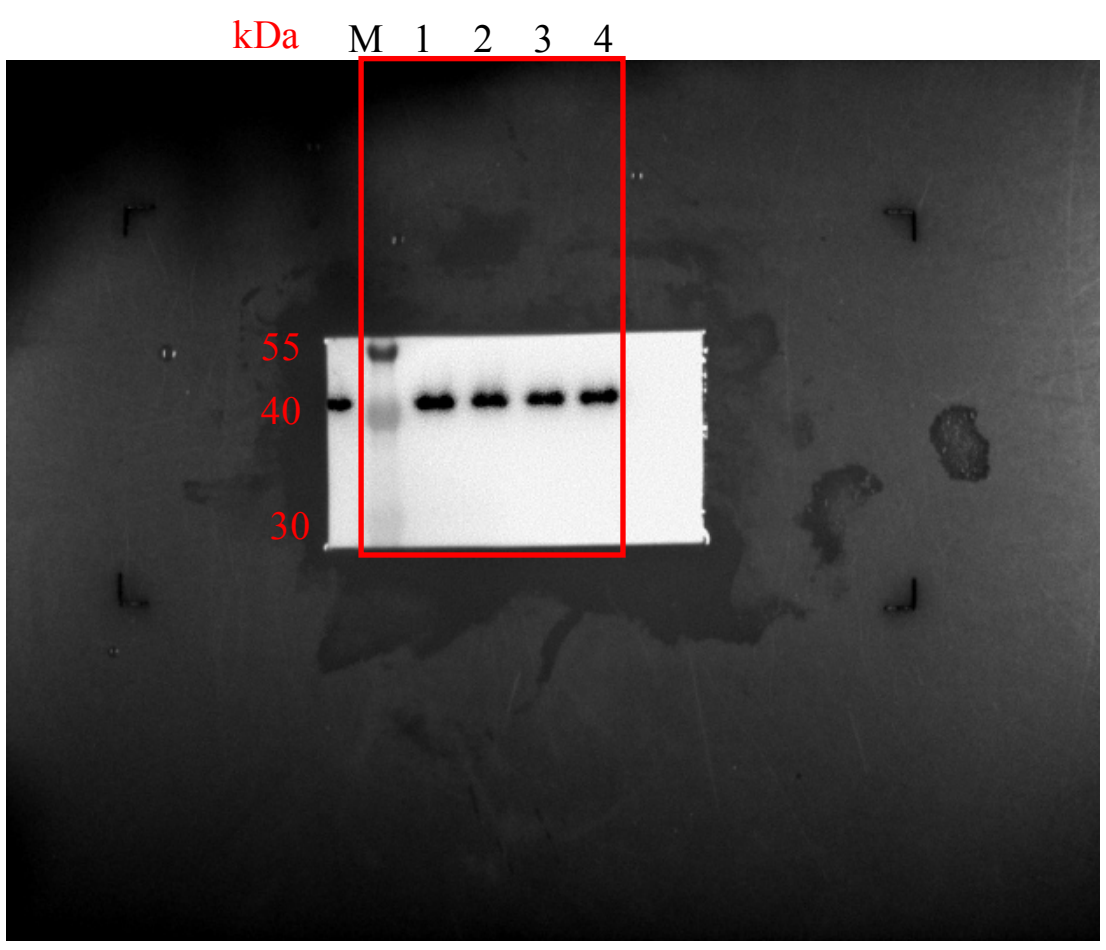

1: CRFK +pCAGGS-Flag (Mock)
2: CRFK +0.25 μg pCAGGS-ZC3HAV1
3: CRFK +0.5 μg pCAGGS-ZC3HAV1
4: CRFK +1 μg pCAGGS-ZC3HAV1

FIG 2I (panel 1)

Anti-ZC3HAV1

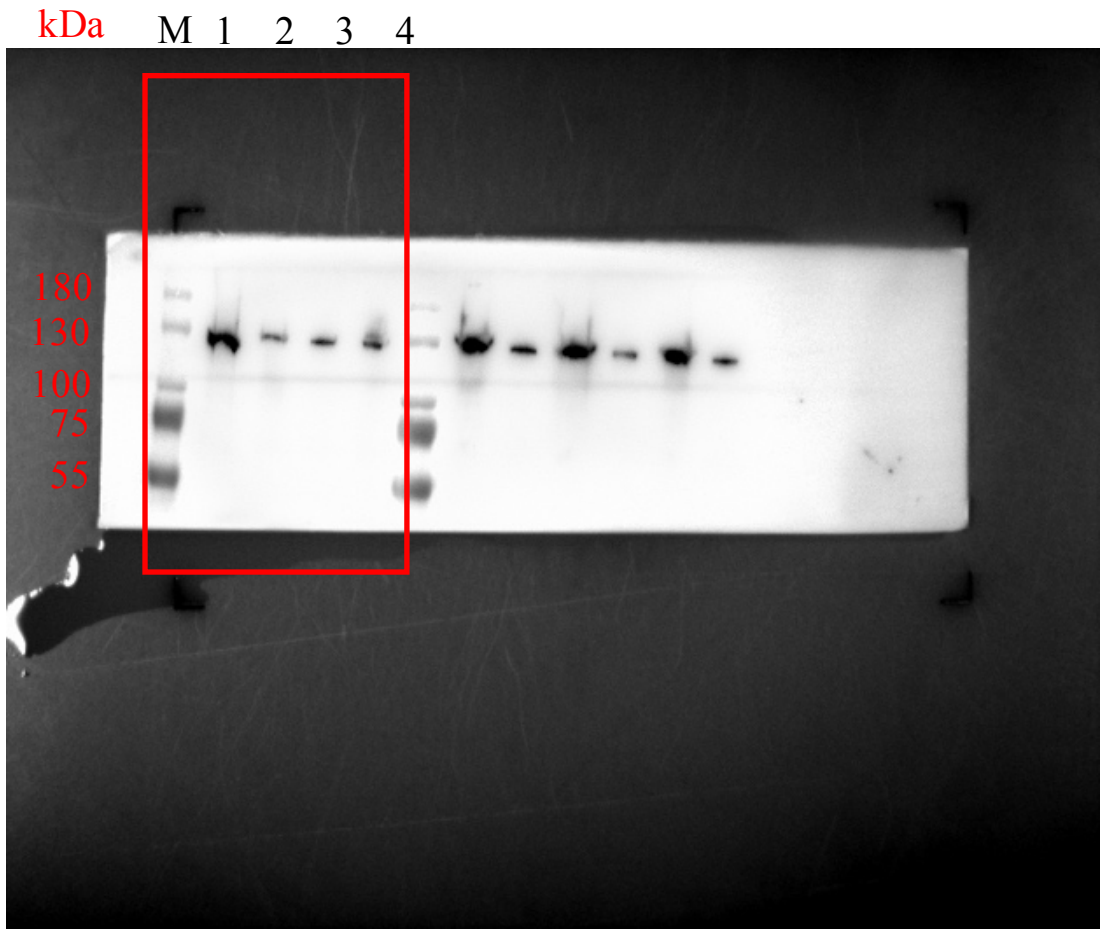

1: CRFK siNC(Mock)
2: CRFK siZC3HAV1-1
3: CRFK siZC3HAV1-2
4: CRFK siZC3HAV1-3

FIG 2I (panel 2)

Anti-β-actin

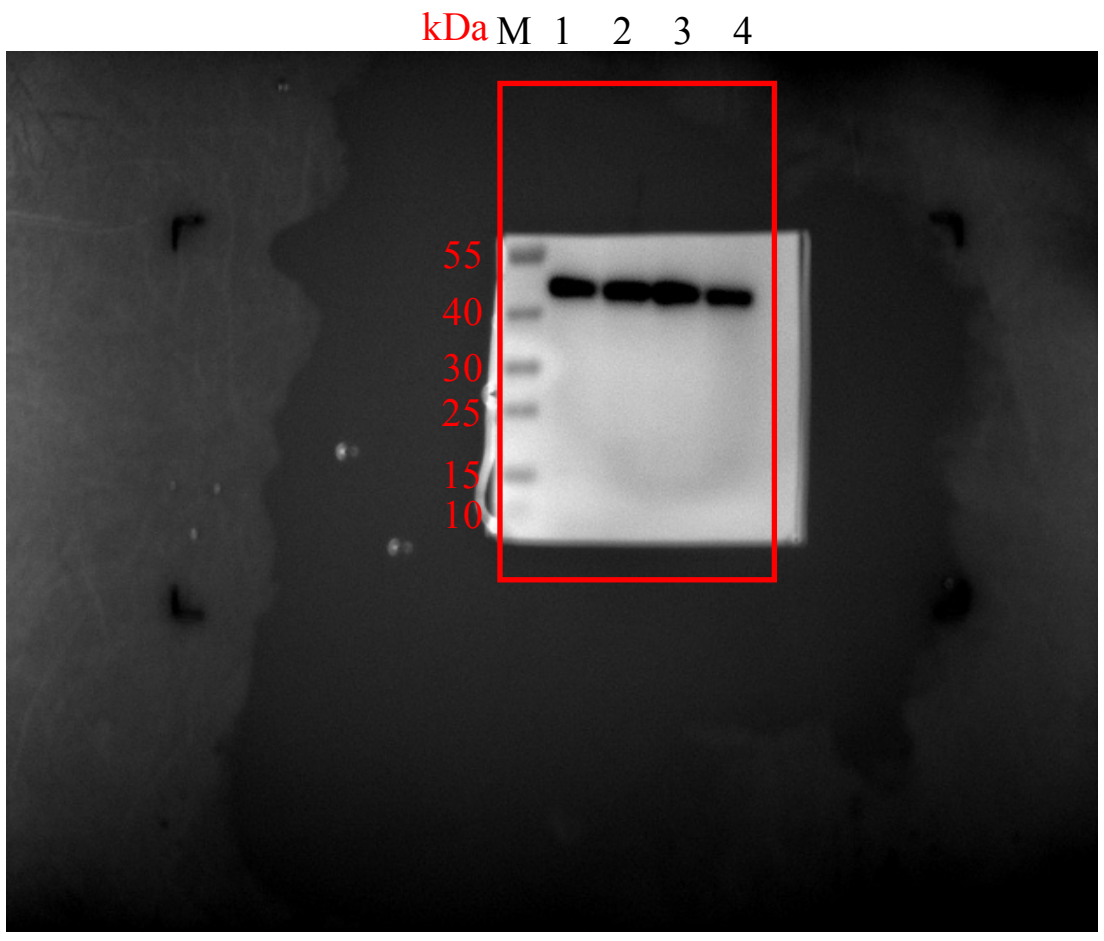

1: CRFK (Mock)
2: CRFK siZC3HAV1-1
3: CRFK siZC3HAV1-2
4: CRFK siZC3HAV1-3

FIG 2K (panel 1)

Anti-ZC3HAV1

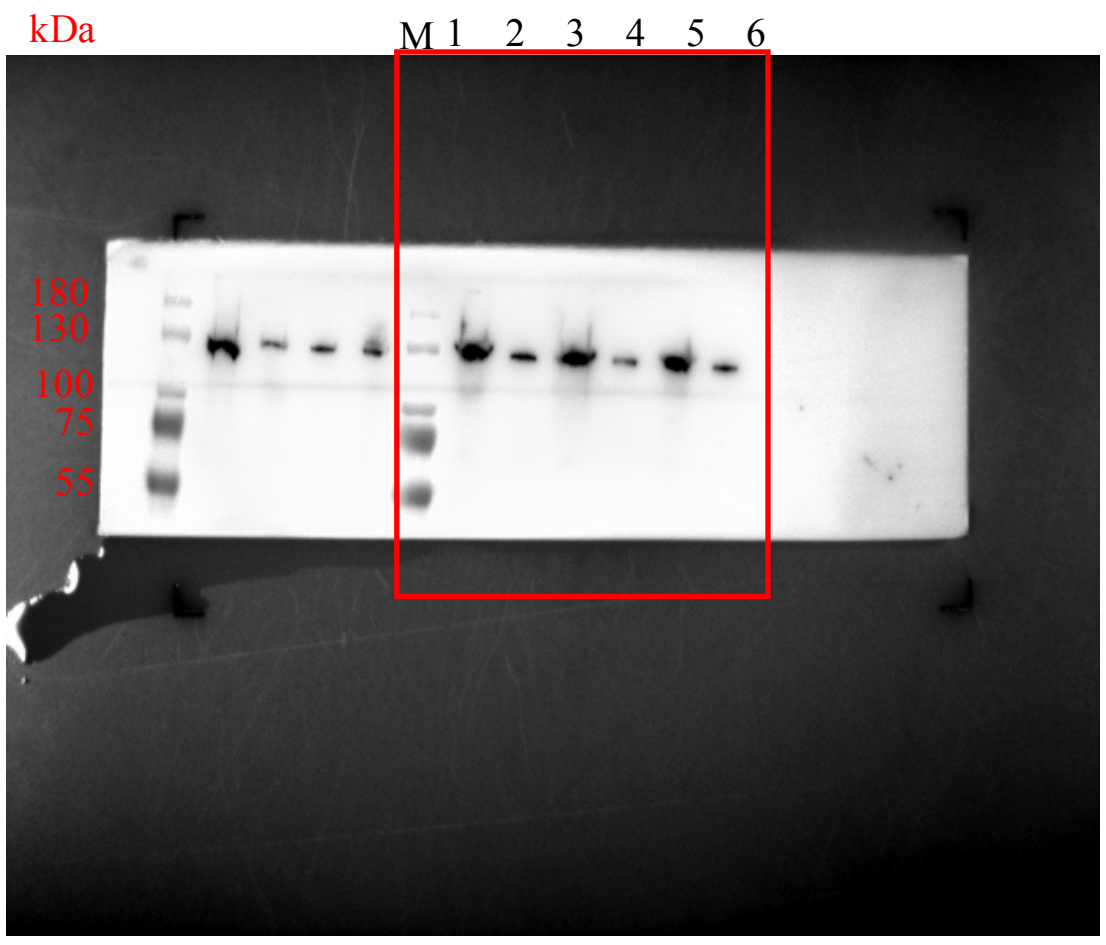

1: CRFK siNC+FIPV 12 hpi (Mock)
2: CRFK siZC3HAV1-1+FIPV 12 hpi
3: CRFK siNC+FIPV 16 hpi (Mock)
4: CRFK siZC3HAV1-1+FIPV 16 hpi
5: CRFK siNC+FIPV 24 hpi (Mock)
6: CRFK siZC3HAV1-1+FIPV 24 hpi

FIG 2K (panel 2)

Anti-FIPV N

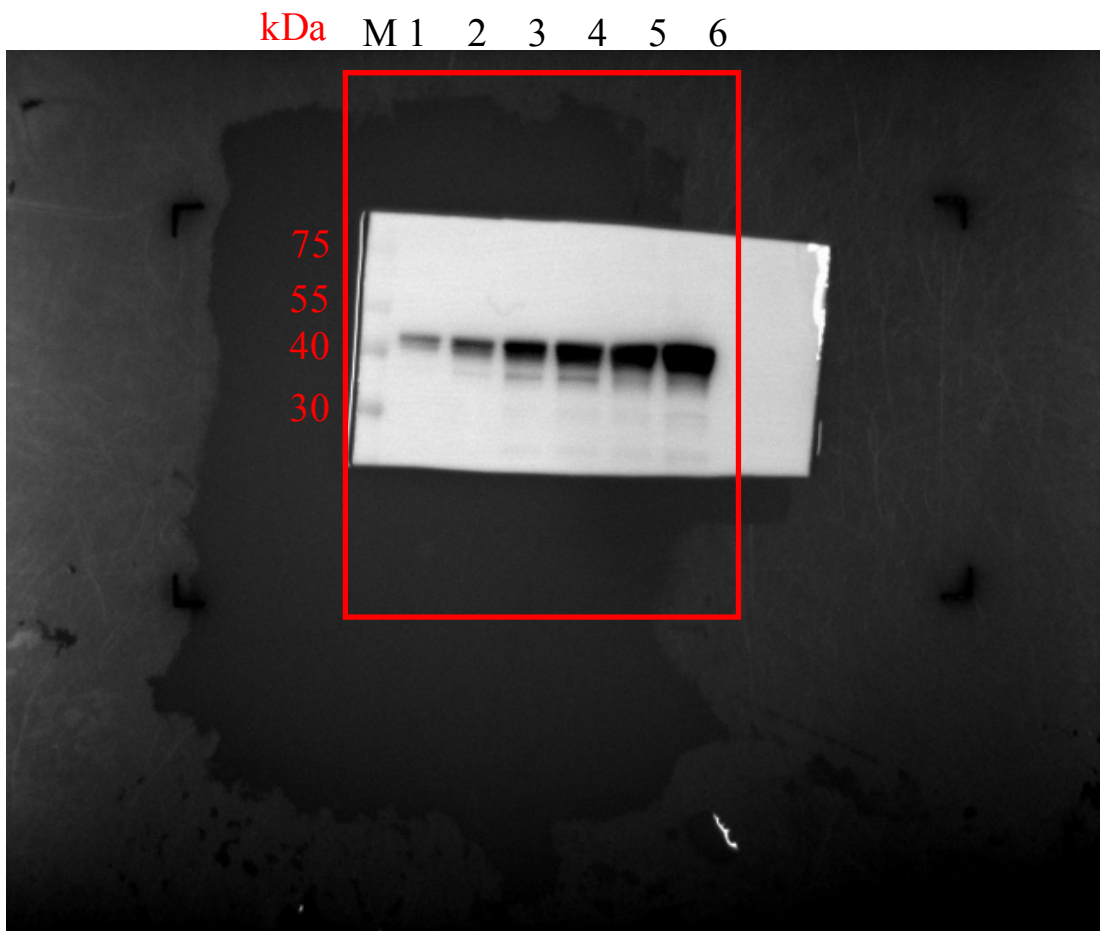

1: CRFK siNC+FIPV 12 hpi (Mock)
2: CRFK siZC3HAV1-1+FIPV 12 hpi
3: CRFK siNC+FIPV 16 hpi (Mock)
4: CRFK siZC3HAV1-1+FIPV 16 hpi
5: CRFK siNC+FIPV 24 hpi (Mock)
6: CRFK siZC3HAV1-1+FIPV 24 hpi

FIG 2K (panel 3)

Anti-β-actin

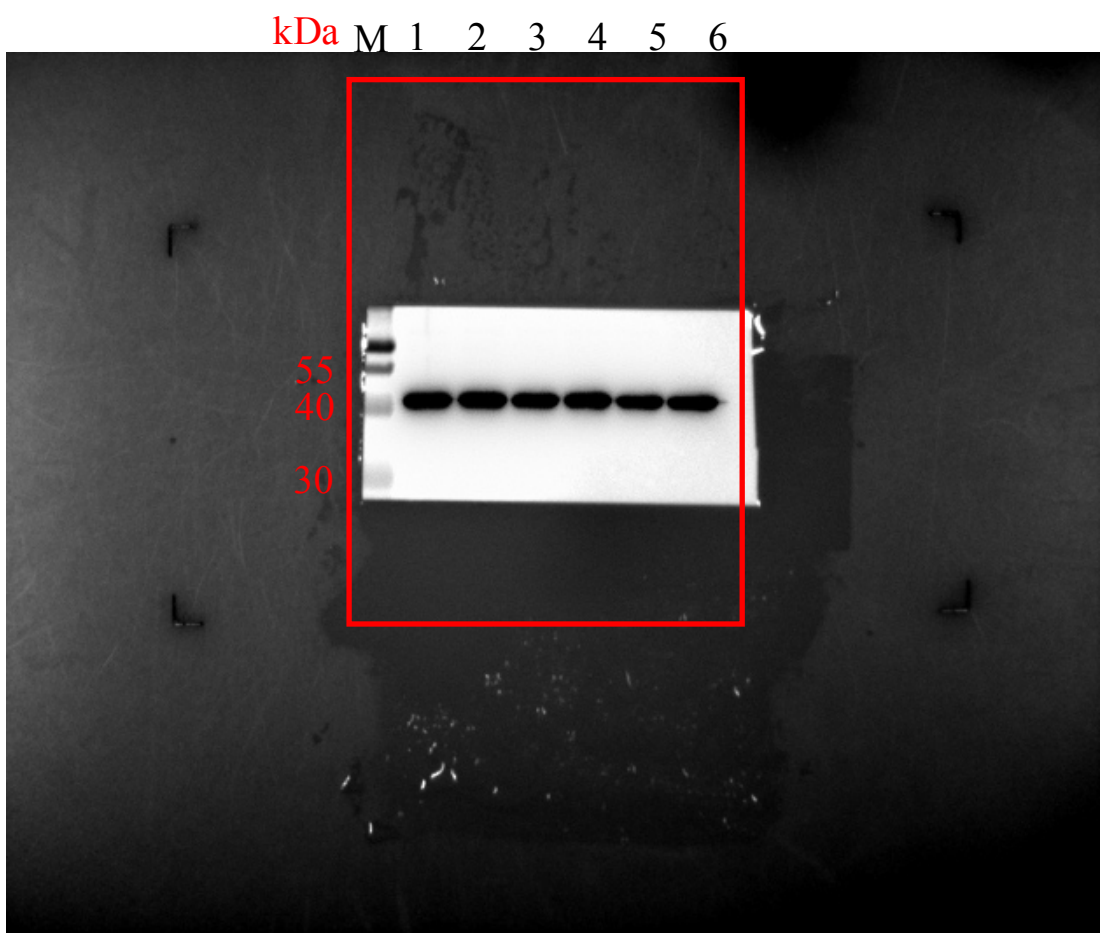

1: CRFK siNC+FIPV 12 hpi (Mock)
2: CRFK siZC3HAV1-1+FIPV 12 hpi
3: CRFK siNC+FIPV 16 hpi (Mock)
4: CRFK siZC3HAV1-1+FIPV 16 hpi
5: CRFK siNC+FIPV 24 hpi (Mock)
6: CRFK siZC3HAV1-1+FIPV 24 hpi

FIG 3B

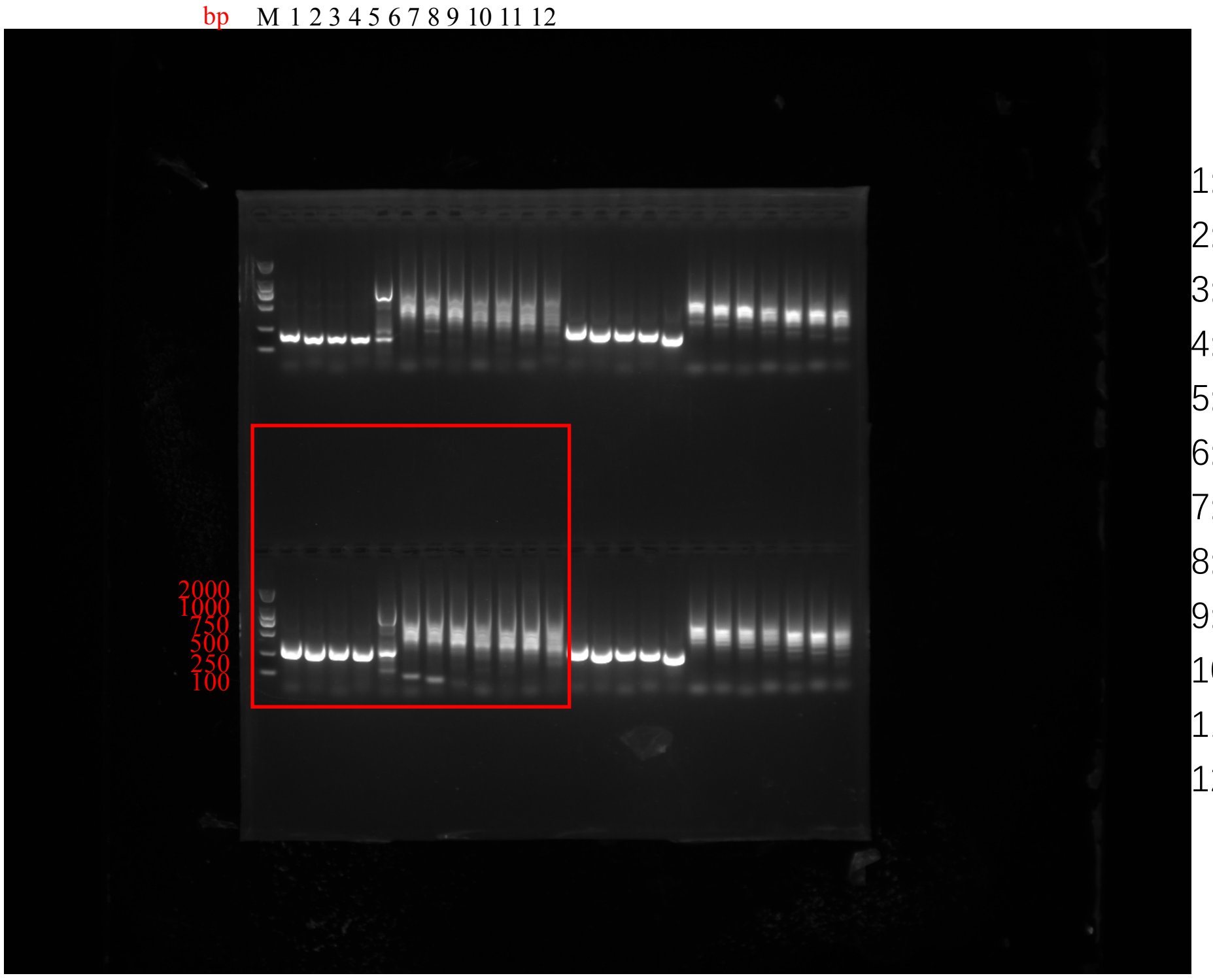

1: Lp1+N-sgRNA-R
2: Lp2+N-sgRNA-R
3: Lp3+N-sgRNA-R
4: Lp4+N-sgRNA-R
5: Lp5+N-sgRNA-R
6: Lp6+N-sgRNA-R
7: Lp7+N-sgRNA-R
8: Lp8+N-sgRNA-R
9: Lp9+N-sgRNA-R
10: Lp10+N-sgRNA-R
11: Lp11+N-sgRNA-R
12: Lp12+N-sgRNA-R

FIG 4A (panel 1)

Anti-Flag

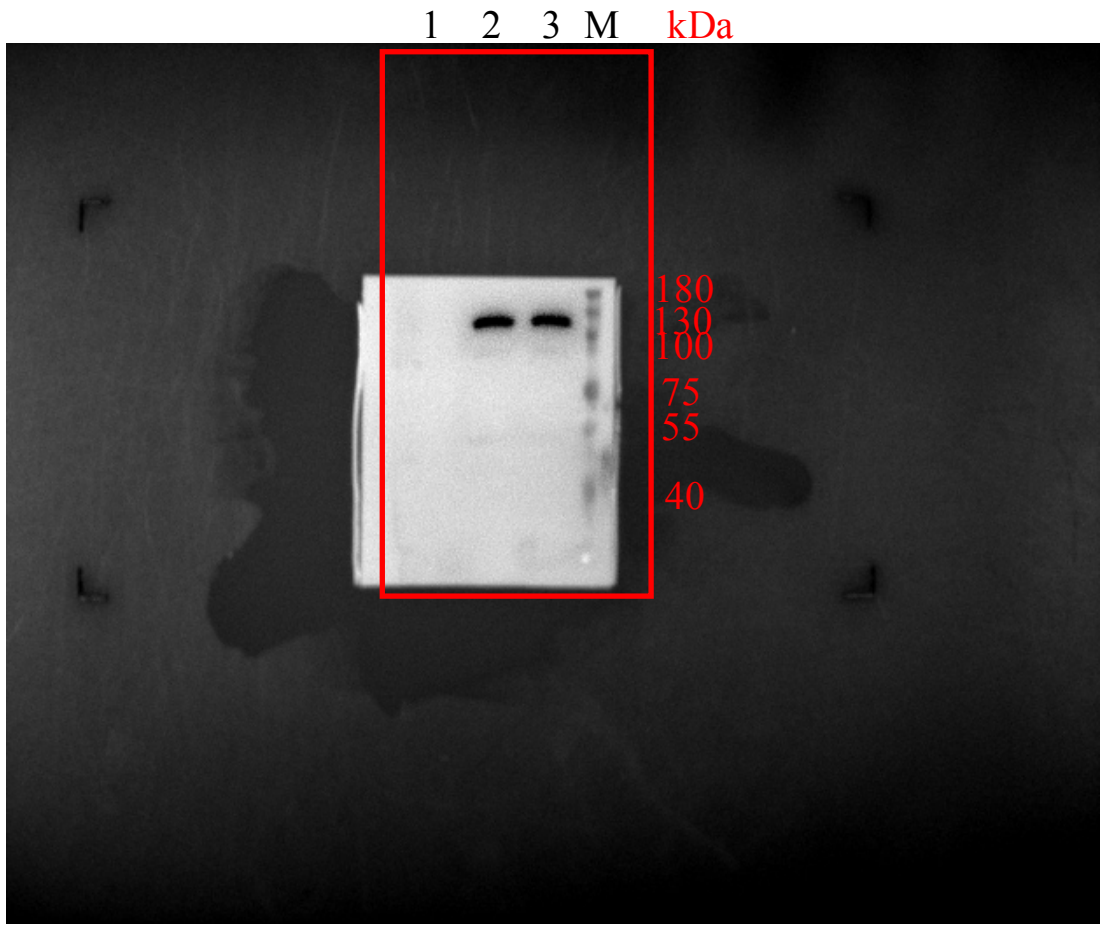

1: Input CRFK+FIPV (pCAGGS-Flag)
2: Input CRFK+FIPV (pCAGGS-ZC3HAV1)
IgG Control
3: Input CRFK+FIPV (pCAGGS-ZC3HAV1)

FIG 4A (panel 2)

Anti-FIPV N

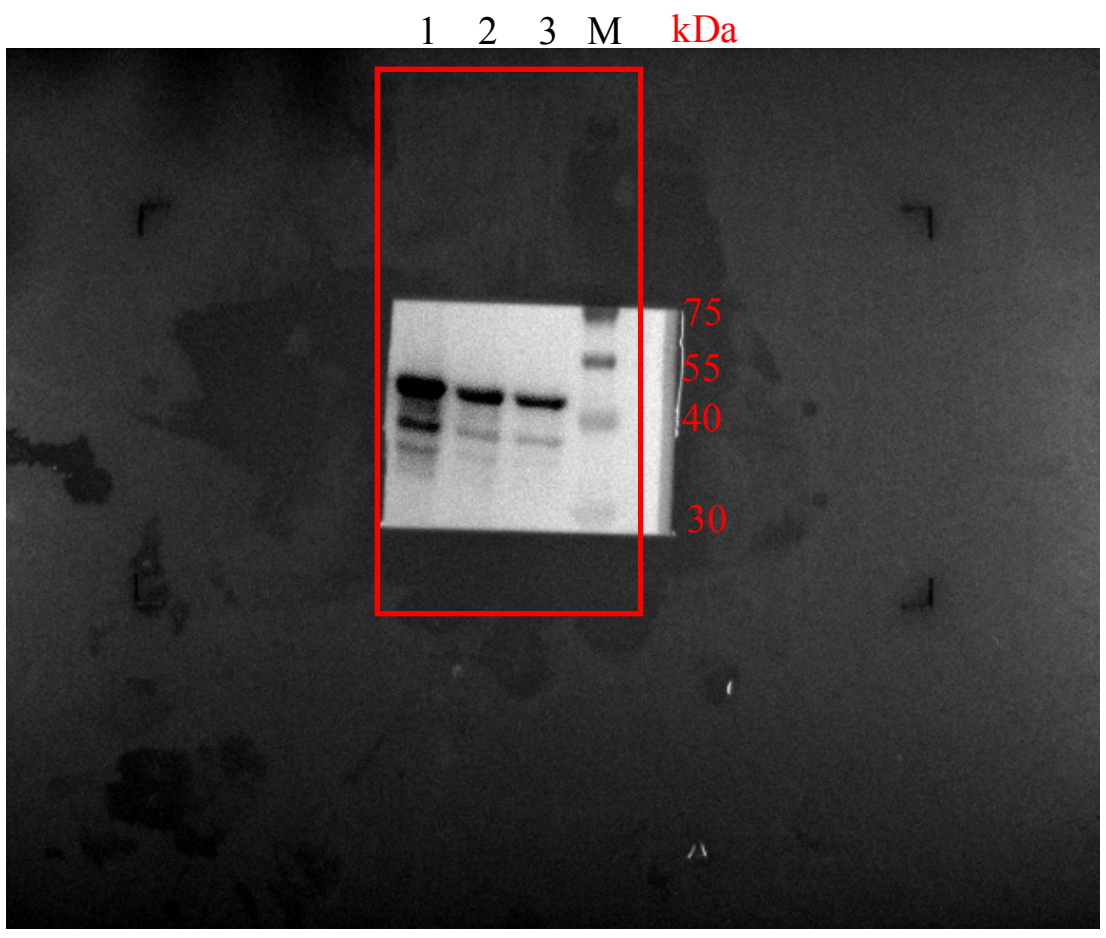

1: Input CRFK+FIPV (pCAGGS-Flag)
2: Input CRFK+FIPV (pCAGGS-ZC3HAV1)
IgG Control
3: Input CRFK+FIPV (pCAGGS-ZC3HAV1)

FIG 4A (panel 3)

Anti-Flag

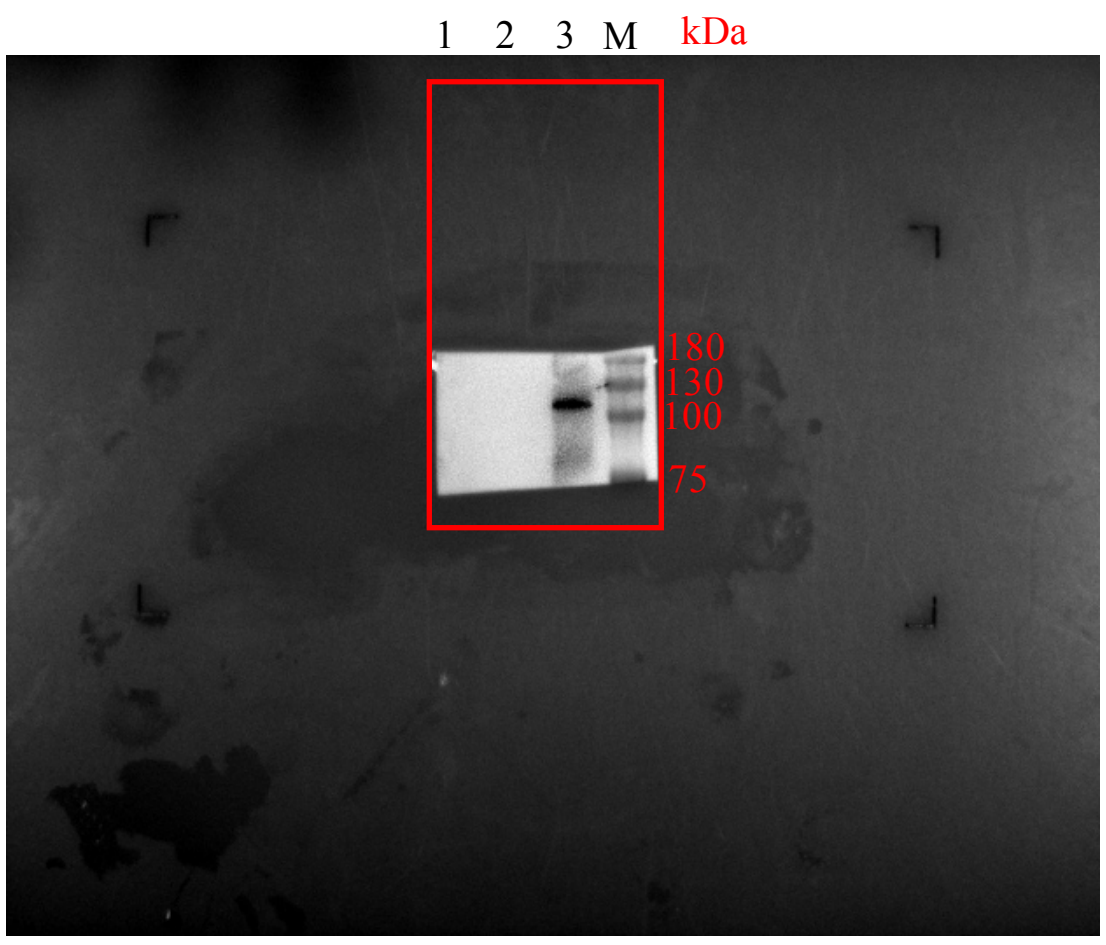

1: Ip (Flag) CRFK+FIPV (pCAGGS-Flag)
2: Ip (IgG) CRFK+FIPV (pCAGGS-ZC3HAV1)
IgG Control
3: Ip (Flag) CRFK+FIPV (pCAGGS-ZC3HAV1)

FIG 4A (panel 4)

Anti-FIPV N

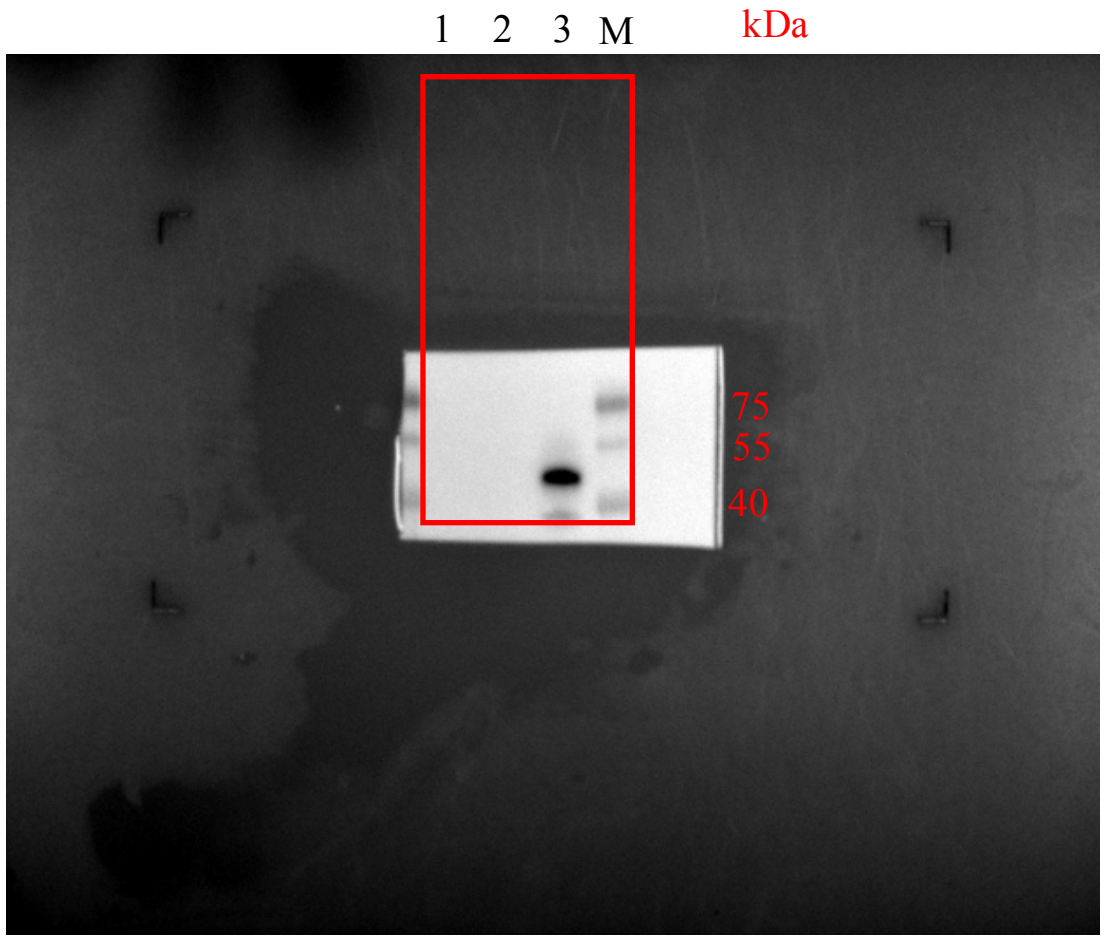

1: Ip (Flag) CRFK+FIPV (pCAGGS-Flag)

2: Ip (IgG) CRFK+FIPV (pCAGGS-ZC3HAV1) IgG Control

3: Ip (Flag) CRFK+FIPV (pCAGGS-ZC3HAV1)

FIG 4B (panel 1+ panel 3)

Anti-Flag

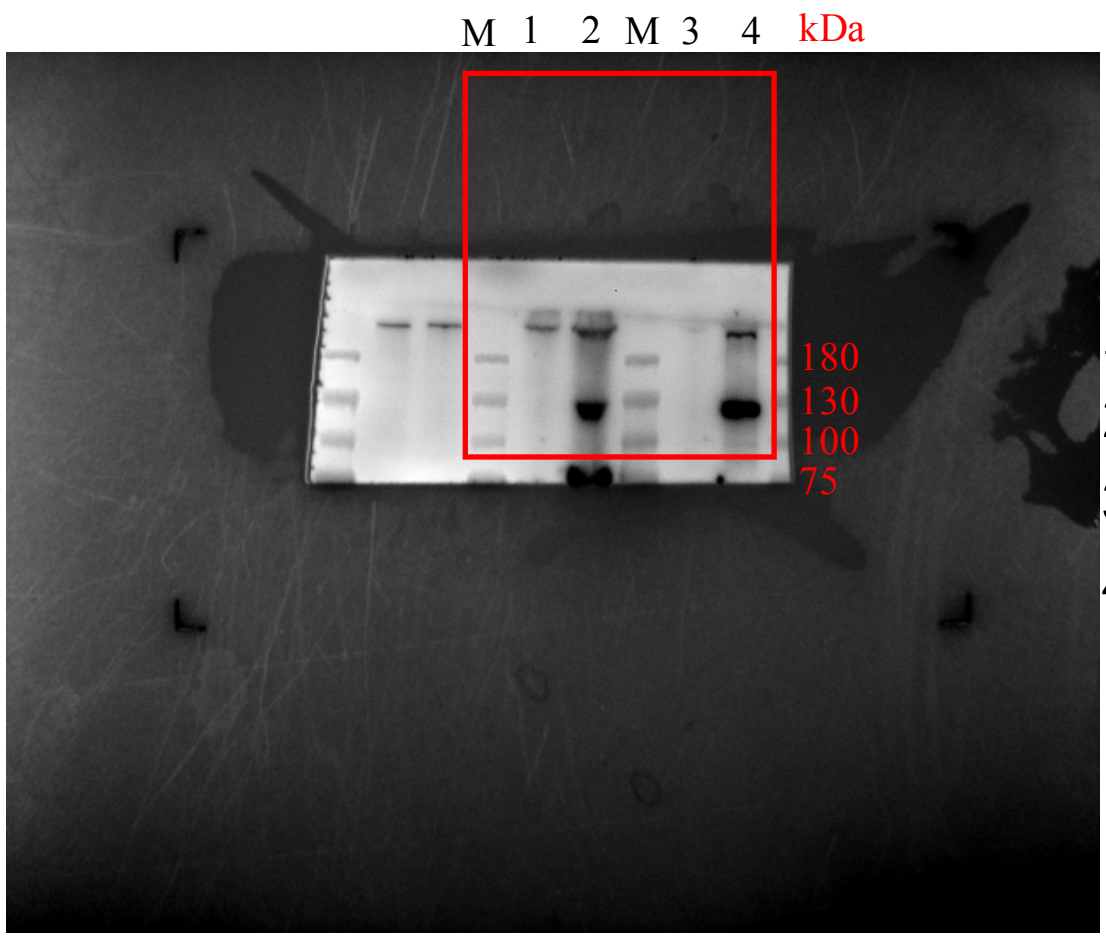

1: Input 293T (pCAGGS-Flag+Myc-N)

2: Input 293T (pCAGGS-ZC3HAV1+ Myc-N)

3: Ip (Flag) 293T (pCAGGS-Flag+Myc-N)

4: Ip (Flag) 293T (pCAGGS-ZC3HAV1+Myc-N)

FIG 4B (panel 2)

Anti-FIPV N

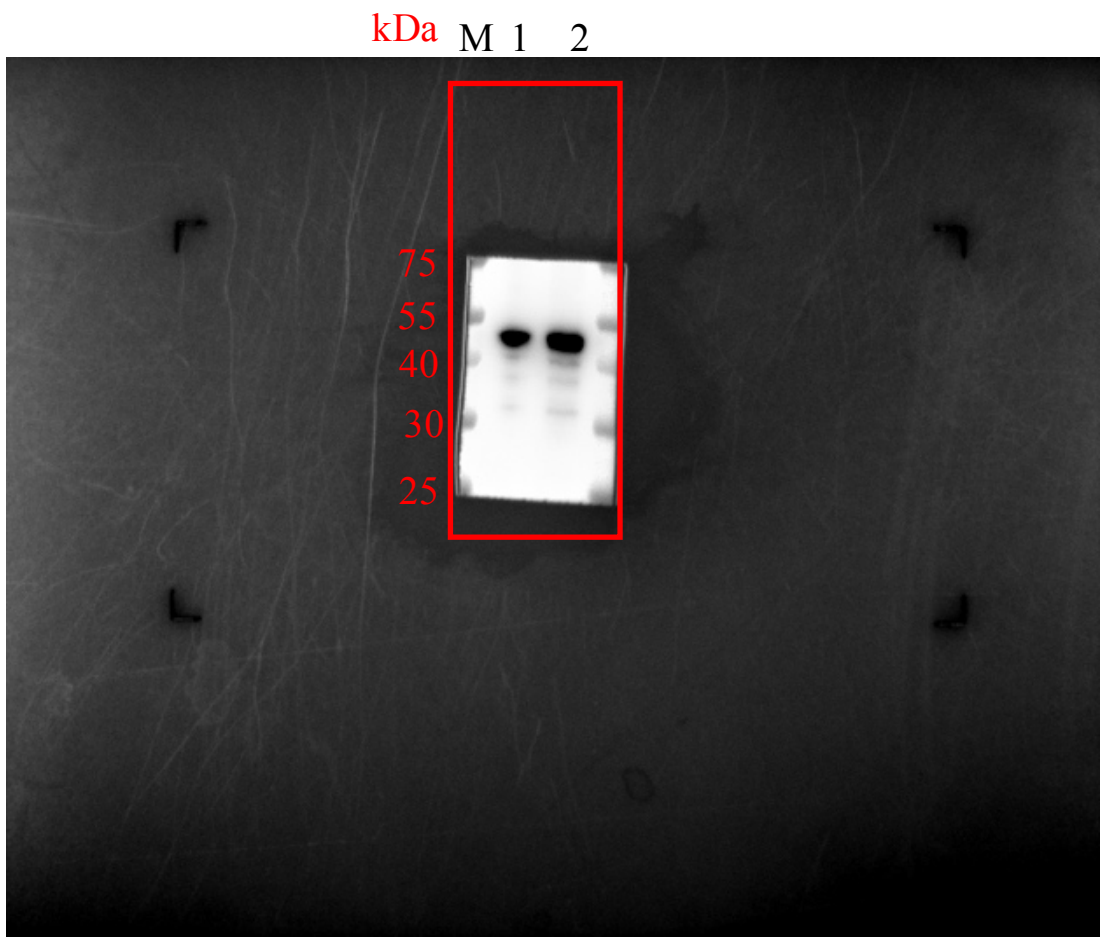

1: Input 293T (pCAGGS-Flag+Myc-N)

2: Input 293T (pCAGGS-ZC3HAV1+ Myc-N)

FIG 4B (panel 4)

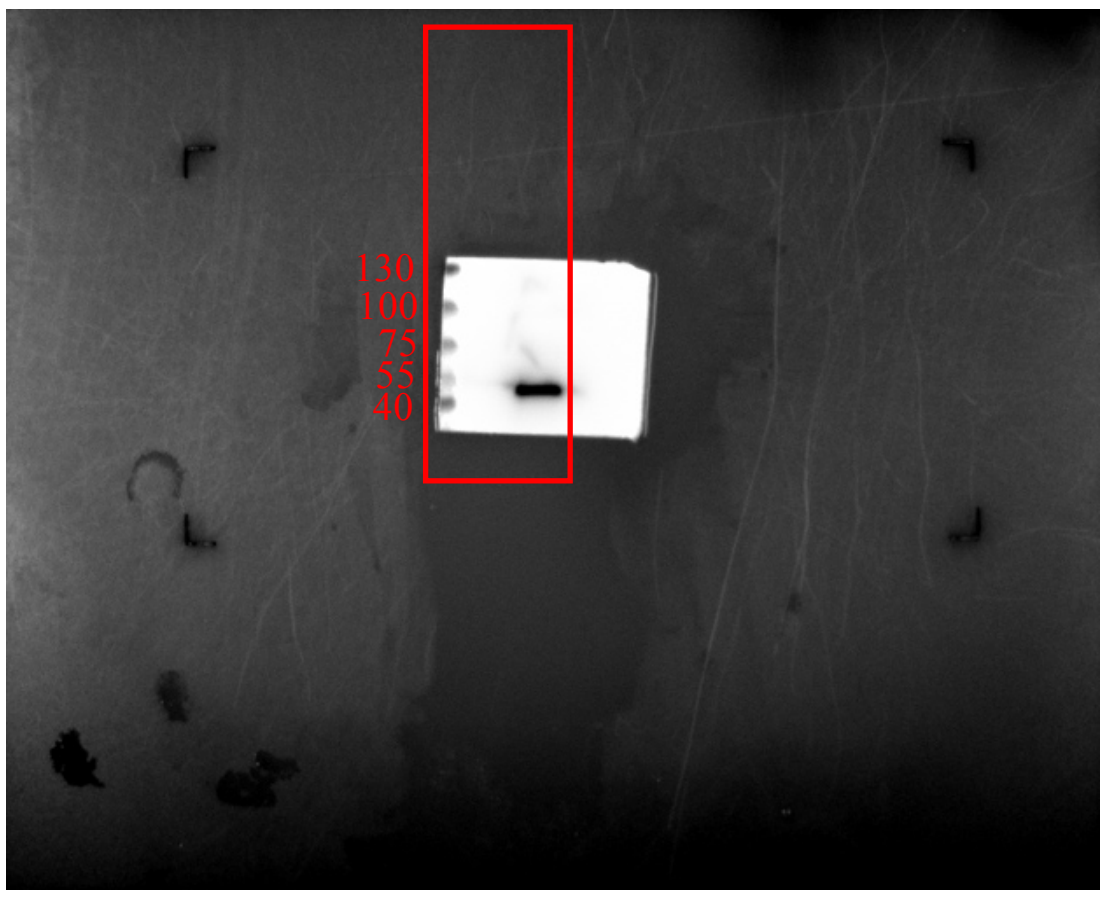

1: Ip (Flag) 293T (pCAGGS-Flag+Myc-N)

2: Ip (Flag) 293T (pCAGGS- ZC3HAV1+Myc-N)

FIG 4C (panel 1)

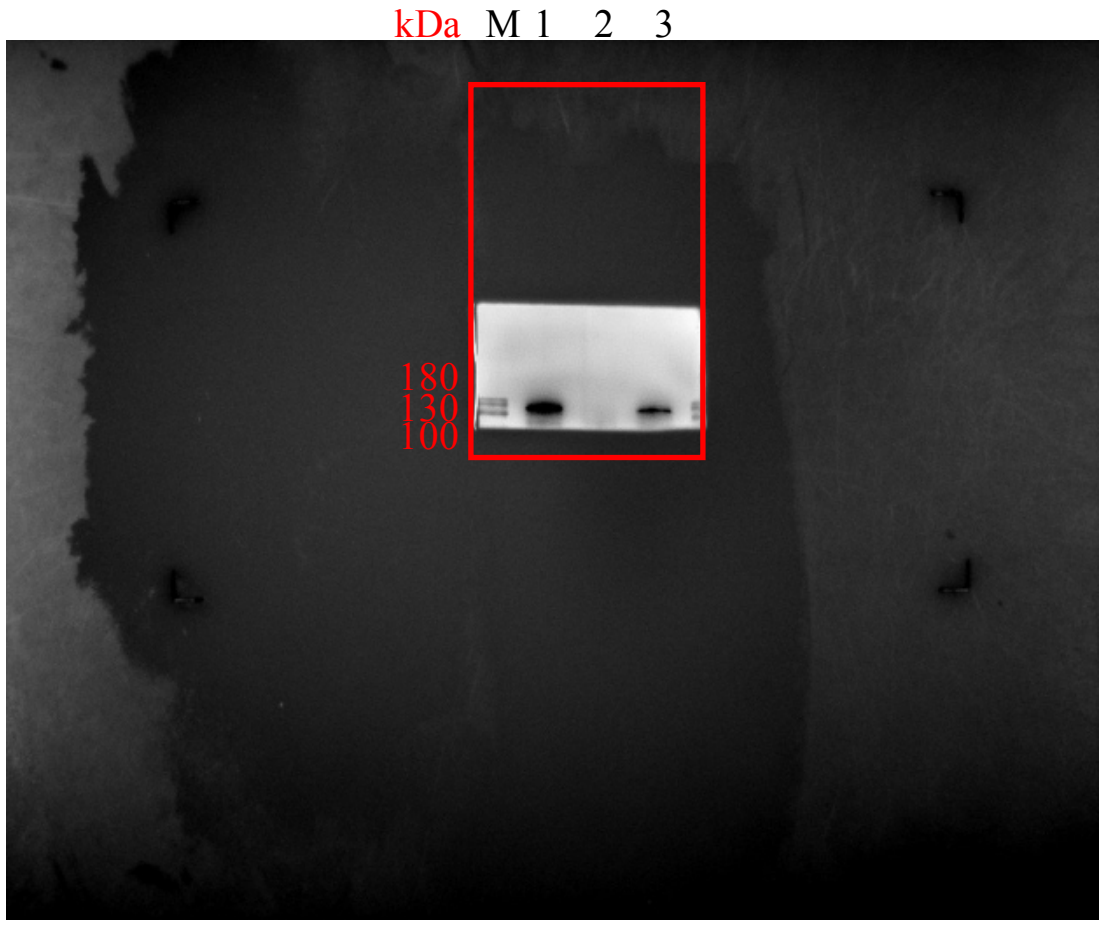

1: Input pET-28a-ZC3HAV1

2: Pull-Down (GST) pEGX-4T-1

3: Pull-Down (GST) pEGX-4T-1-FIPV N

FIG 4C (panel 2)

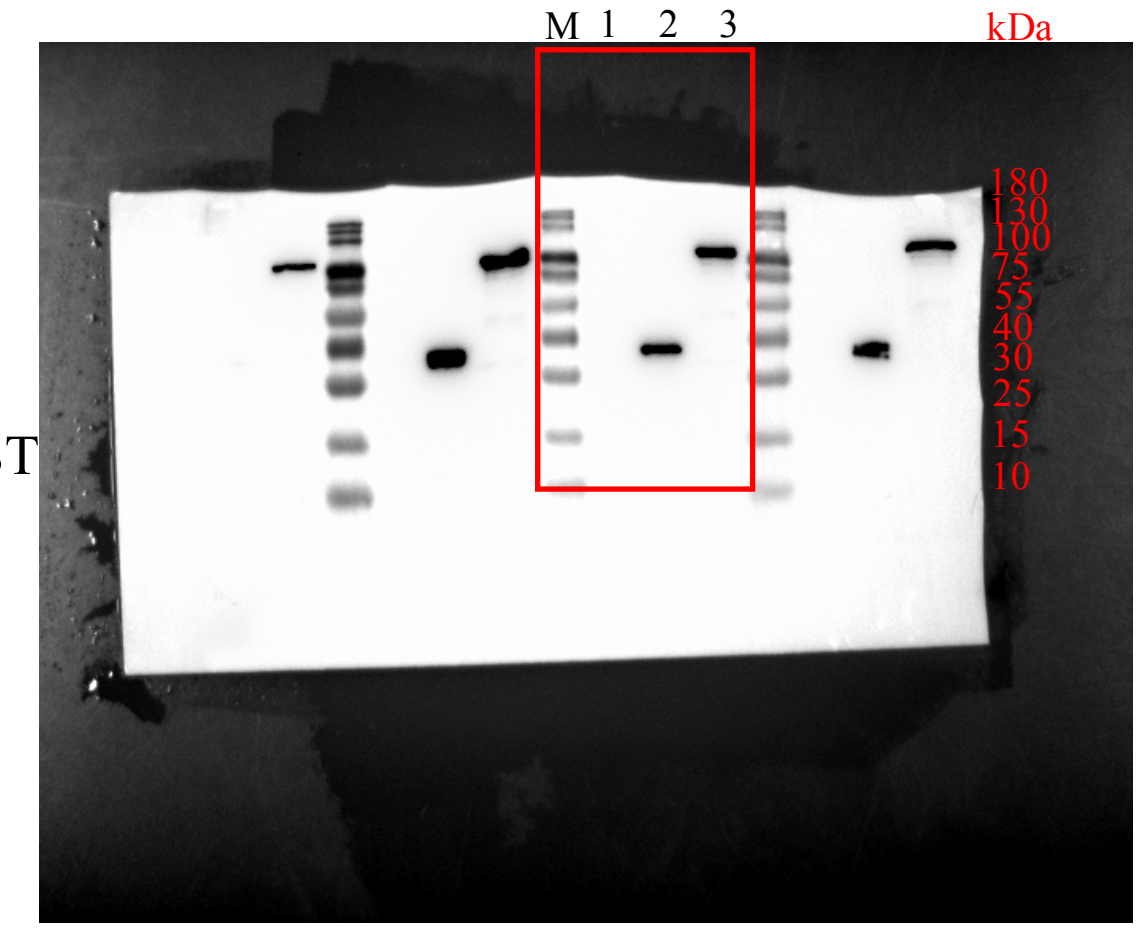

1: Input pET-28a-ZC3HAV1

2: Input pEGX-4T-1

3: Input pEGX-4T-1-FIPV N

FIG 4C (panel 3)

Anti-GST

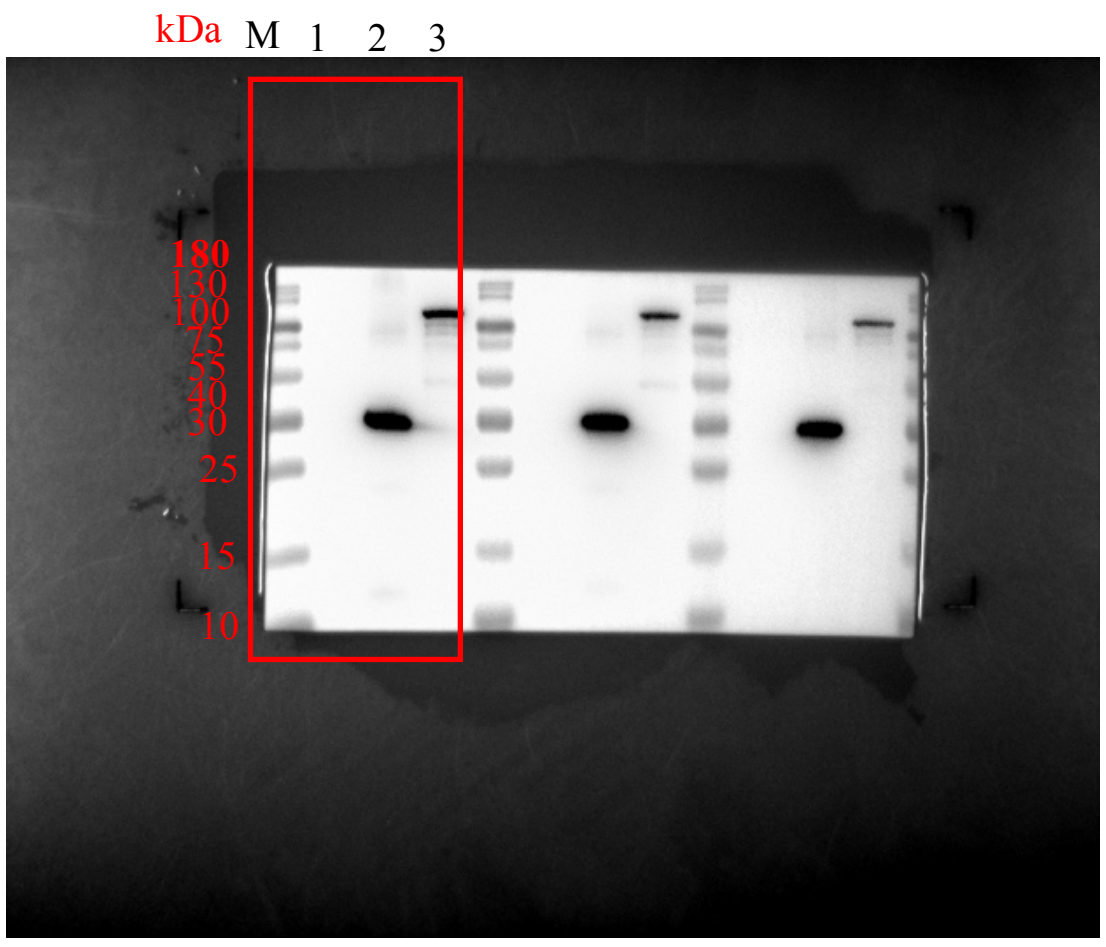

1: Input pET-28a-ZC3HAV1
2: Pull-Down (GST) pEGX-4T-1
3: Pull-Down (GST) pEGX-4T-1-FIPV N

FIG 4G (panel 1)

Anti-HA

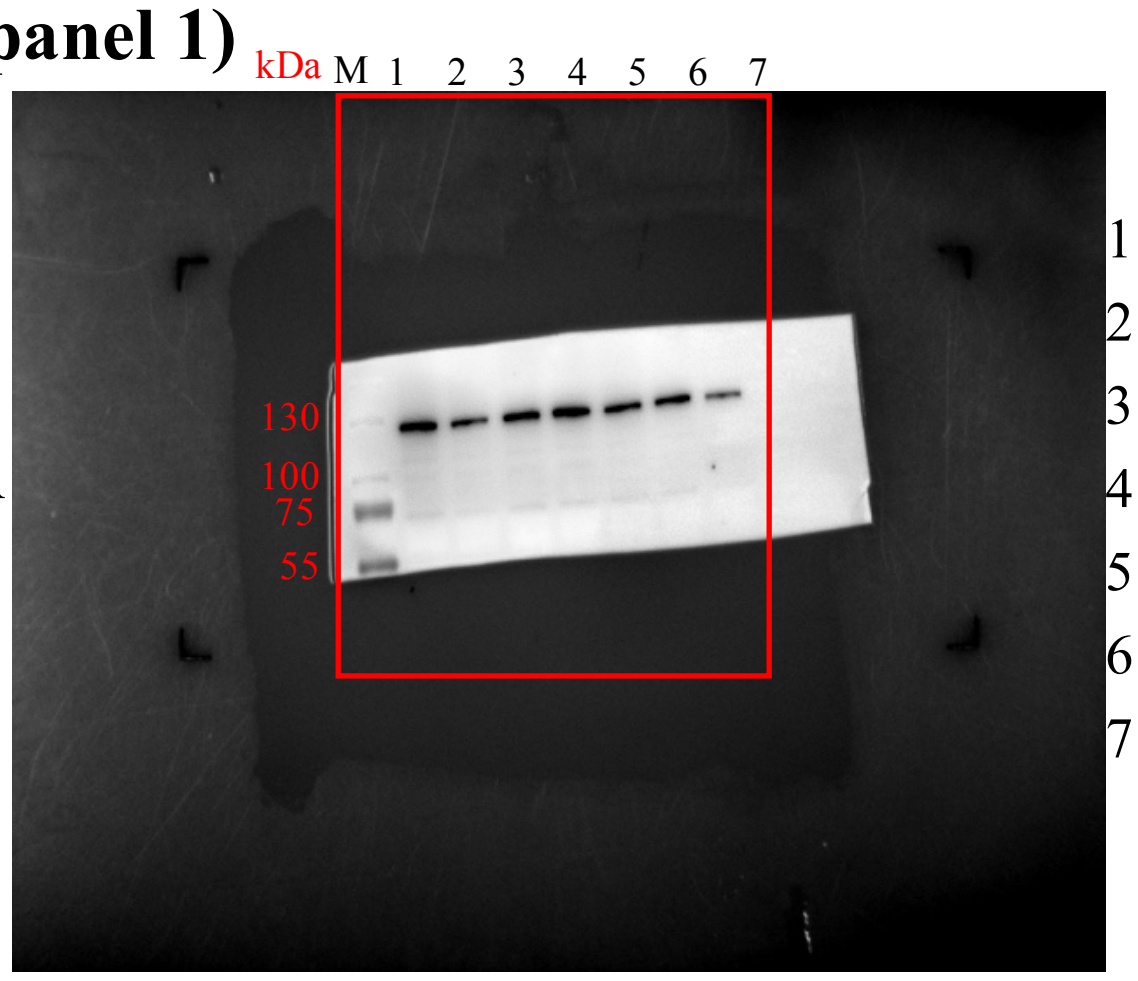

1: Input 293T pCAGGS-GFP+ pCMV14-HA-ZC3HAV1
2: Input 293T pCAGGS-N+ pCMV14-HA-ZC3HAV1
3: Input 293T pCAGGS-N1+ pCMV14-HA-ZC3HAV1
4: Input 293T pCAGGS-N2+ pCMV14-HA-ZC3HAV1
5: Input 293T pCAGGS-N3+ pCMV14-HA-ZC3HAV1
6: Input 293T pCAGGS-N4+ pCMV14-HA-ZC3HAV1
7: Input 293T pCAGGS-N5+ pCMV14-HA-ZC3HAV1

FIG 4G (panel 2)

Anti-Flag

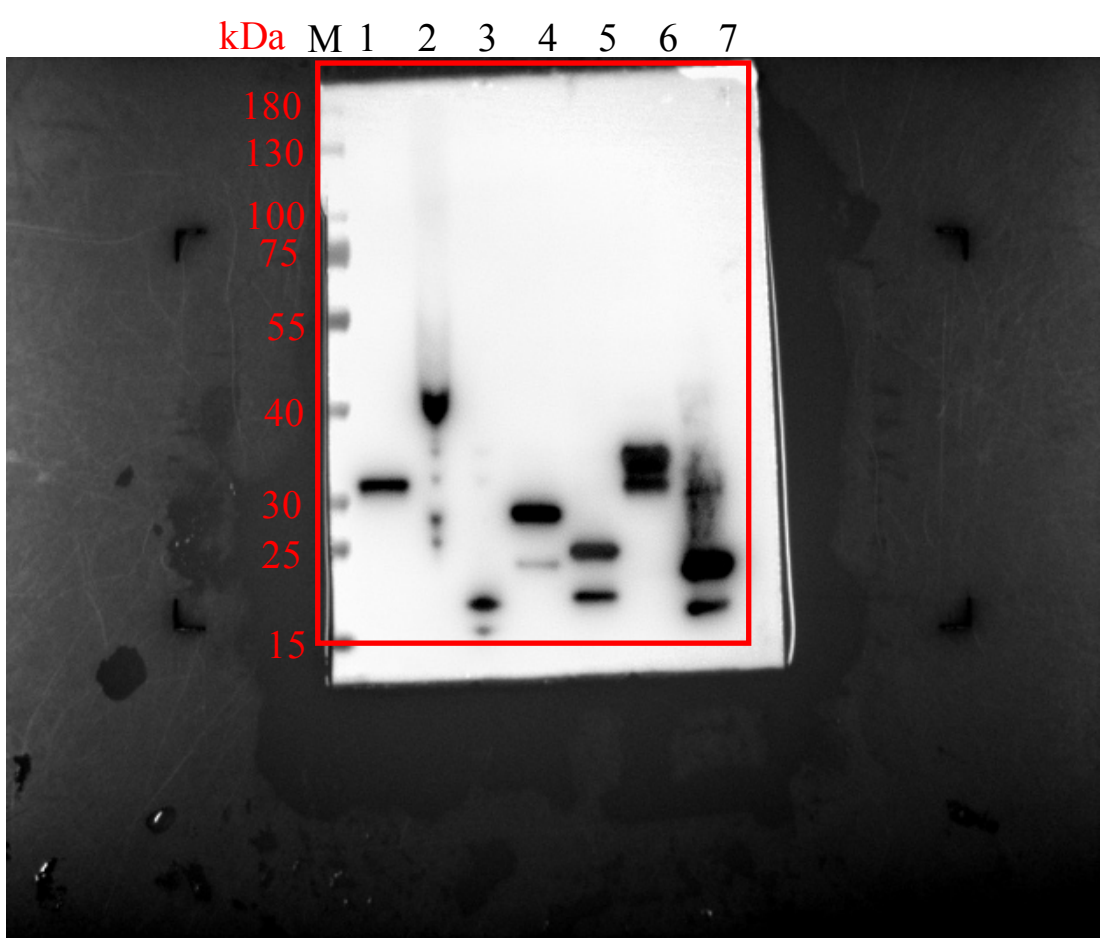

1: Input 293T pCAGGS-GFP+ pCMV14-HA-ZC3HAV1
2: Input 293T pCAGGS-N+ pCMV14-HA-ZC3HAV1
3: Input 293T pCAGGS-N1+ pCMV14-HA-ZC3HAV1
4: Input 293T pCAGGS-N2+ pCMV14-HA-ZC3HAV1
5: Input 293T pCAGGS-N3+ pCMV14-HA-ZC3HAV1
6: Input 293T pCAGGS-N4+ pCMV14-HA-ZC3HAV1
7: Input 293T pCAGGS-N5+ pCMV14-HA-ZC3HAV1

FIG 4G (panel 3)

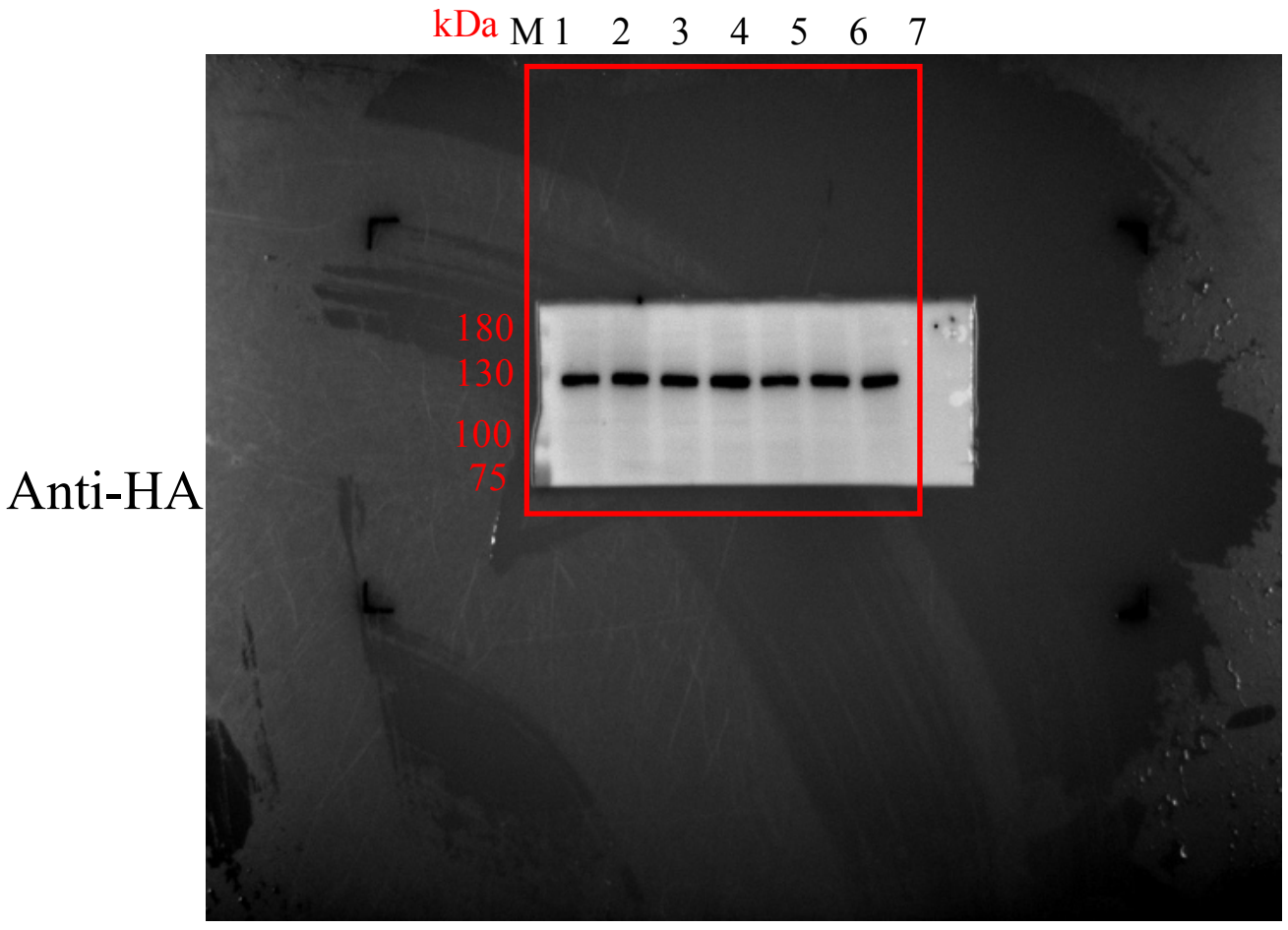

1: Ip (HA) 293T pCAGGS-GFP+ pCMV14-HA-ZC3HAV1
2: Ip (HA) pCAGGS-N+ pCMV14-HA-ZC3HAV1
3: Ip (HA) 293T pCAGGS-N1+ pCMV14-HA-ZC3HAV1
4: Ip (HA) 293T pCAGGS-N2+ pCMV14-HA-ZC3HAV1
5: Ip (HA) 293T pCAGGS-N3+ pCMV14-HA-ZC3HAV1
6: Ip (HA) 293T pCAGGS-N4+ pCMV14-HA-ZC3HAV1
7: Ip (HA) 293T pCAGGS-N5+ pCMV14-HA-ZC3HAV1

FIG 4G (panel 4)

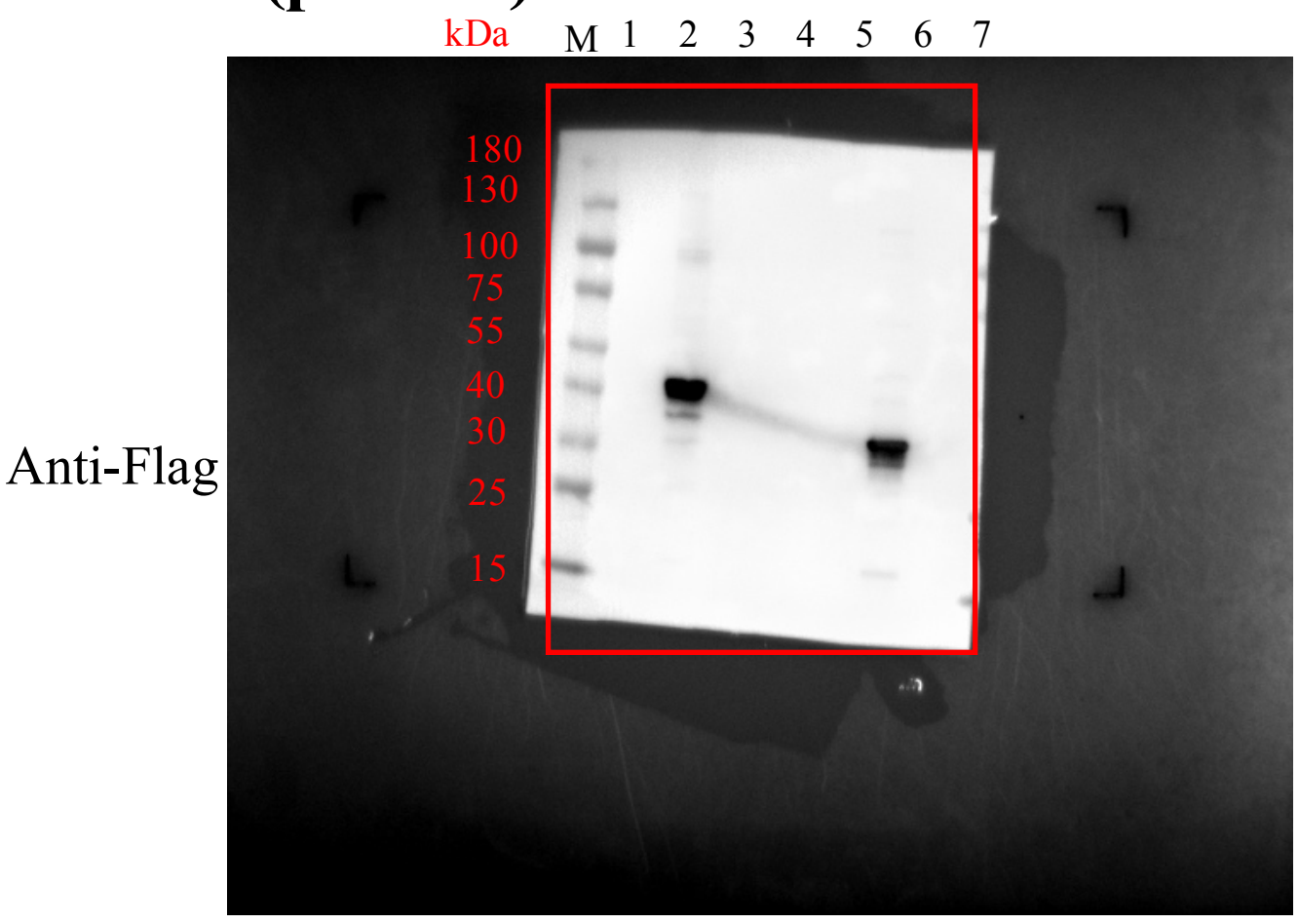

1: Ip (HA) 293T pCAGGS-GFP+ pCMV14-HA-ZC3HAV1
2: Ip (HA) 293T pCAGGS-N+ pCMV14-HA-ZC3HAV1
3: Ip (HA) 293T pCAGGS-N1+ pCMV14-HA-ZC3HAV1
4: Ip (HA) 293T pCAGGS-N2+ pCMV14-HA-ZC3HAV1
5: Ip (HA) 293T pCAGGS-N3+ pCMV14-HA-ZC3HAV1
6: Ip (HA) 293T pCAGGS-N4+ pCMV14-HA-ZC3HAV1
7: Ip (HA) 293T pCAGGS-N5+ pCMV14-HA-ZC3HAV1

FIG 4H (panel 1)

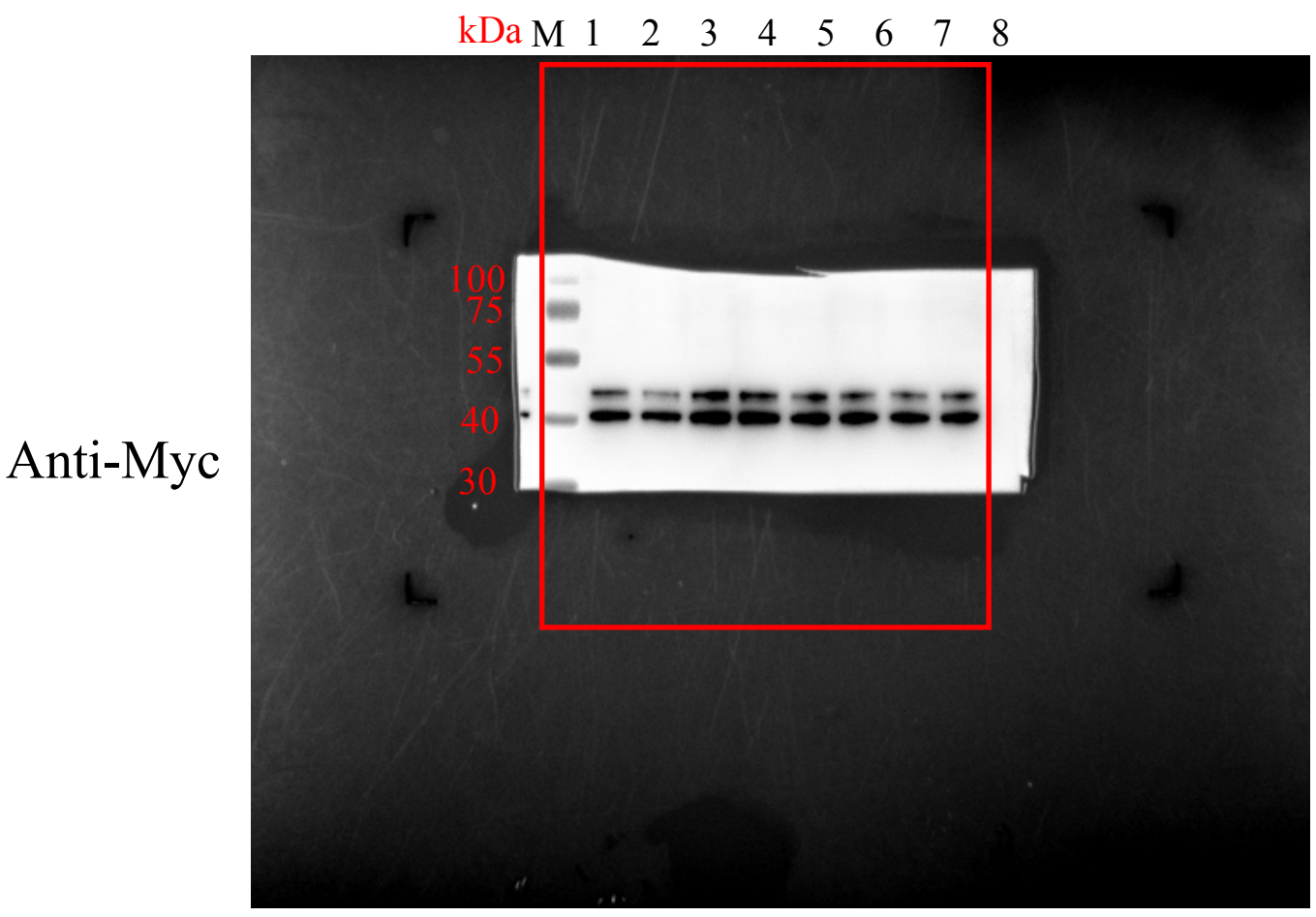

1: Input 293T pCAGGS-GFP+ pCMV14-Myc-N
2: Input 293T pCAGGS-ZC3HAV1+ pCMV14-Myc-N
3: Input 293T pCAGGS-Z1+ pCMV14-Myc-N
4: Input 293T pCAGGS-Z2+ pCMV14-Myc-N
5: Input 293T pCAGGS-Z3+ pCMV14-Myc-N
6: Input 293T pCAGGS-Z4+ pCMV14-Myc-N
7: Input 293T pCAGGS-Z5+ pCMV14-Myc-N
8: Input 293T pCAGGS-Z6+ pCMV14-Myc-N

FIG 4H (panel 2)

Anti-Flag

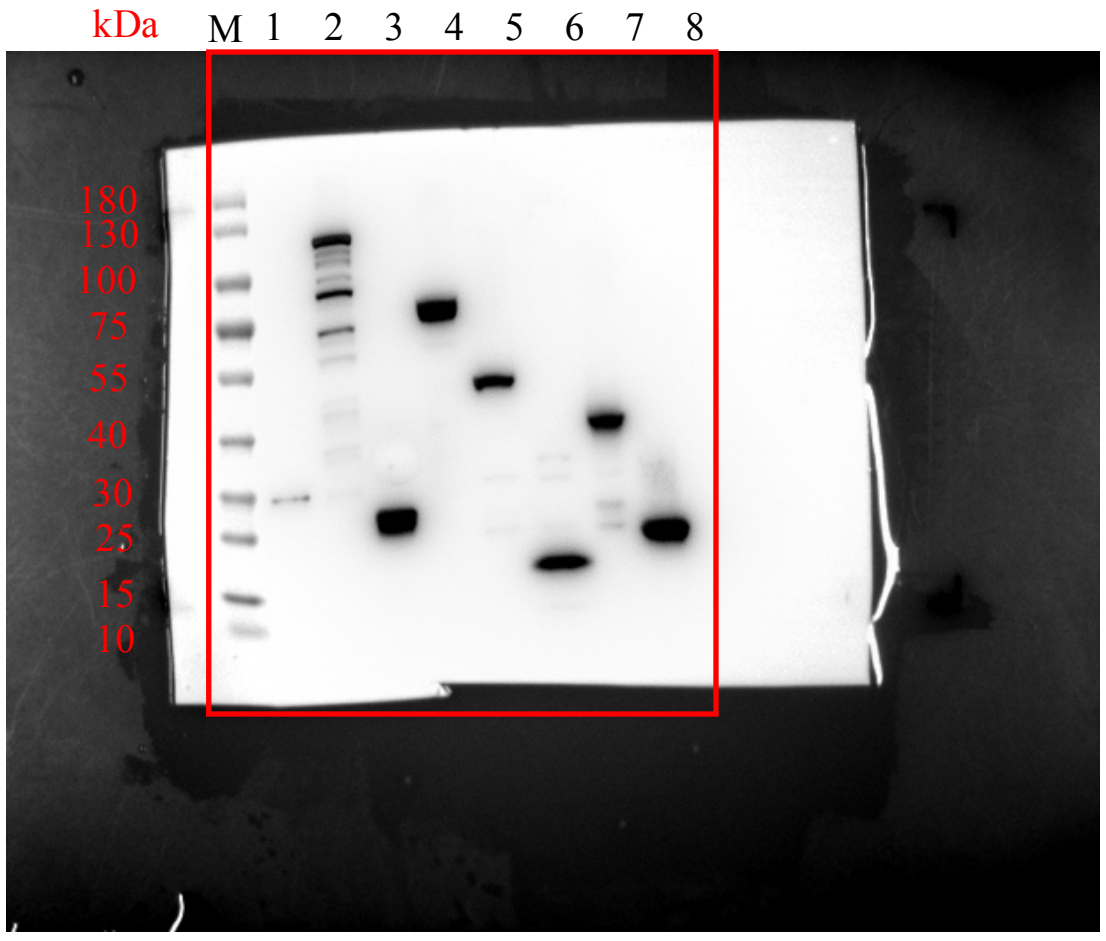

1: Input 293T pCAGGS-GFP+ pCMV14-Myc-N
2: Input 293T pCAGGS-ZC3HAV1+ pCMV14-Myc-N
3: Input 293T pCAGGS-Z1+ pCMV14-Myc-N
4: Input 293T pCAGGS-Z2+ pCMV14-Myc-N
5: Input 293T pCAGGS-Z3+ pCMV14-Myc-N
6: Input 293T pCAGGS-Z4+ pCMV14-Myc-N
7: Input 293T pCAGGS-Z5+ pCMV14-Myc-N
8: Input 293T pCAGGS-Z6+ pCMV14-Myc-N

FIG 4H (panel 3)

Anti-Myc

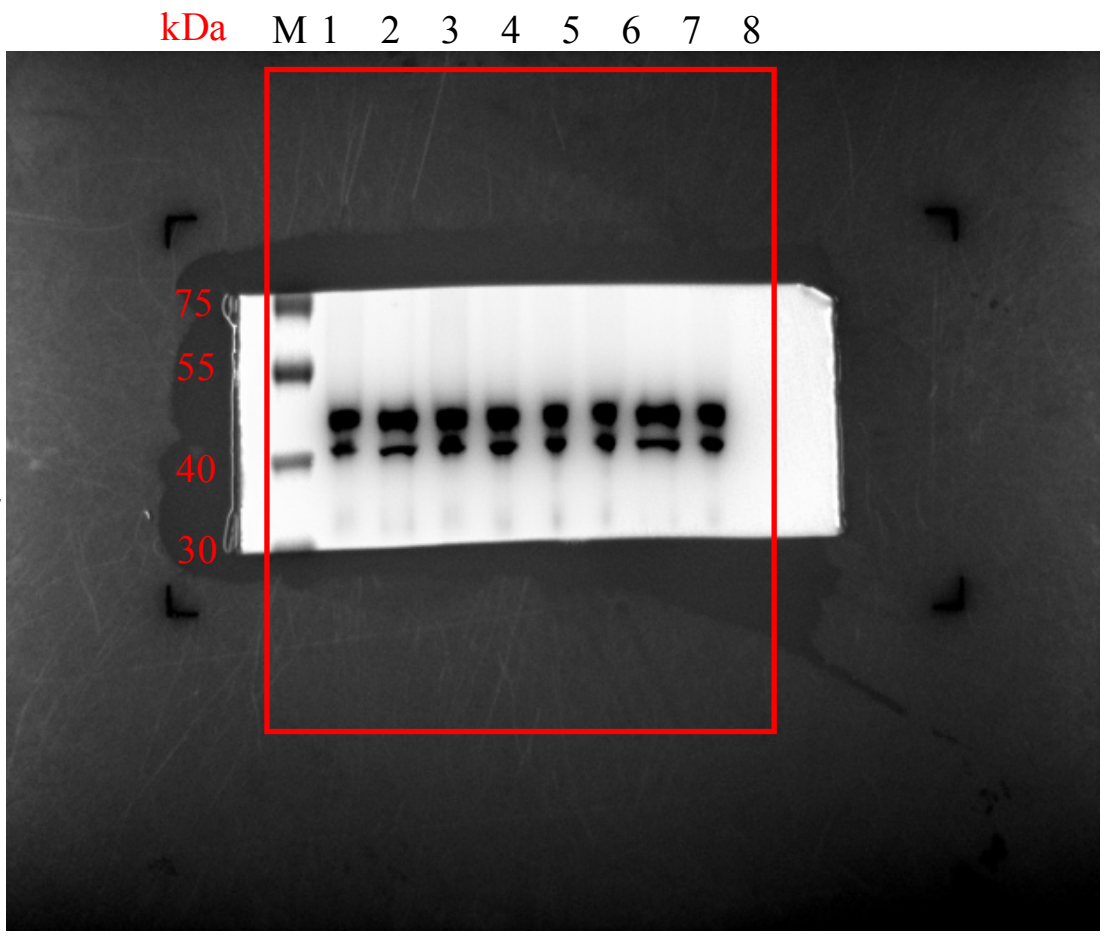

1: Ip (Myc) 293T pCAGGS-GFP+ pCMV14-Myc-N
2: Ip (Myc) 293T pCAGGS-ZC3HAV1+ pCMV14-Myc-N
3: Ip (Myc) 293T pCAGGS-Z1+ pCMV14-Myc-N
4: Ip (Myc) 293T pCAGGS-Z2+ pCMV14-Myc-N
5: Ip (Myc) 293T pCAGGS-Z3+ pCMV14-Myc-N
6: Ip (Myc) 293T pCAGGS-Z4+ pCMV14-Myc-N
7: Ip (Myc) 293T pCAGGS-Z5+ pCMV14-Myc-N
8: Ip (Myc) 293T pCAGGS-Z6+ pCMV14-Myc-N

FIG 4H (panel 4)

Anti-Flag

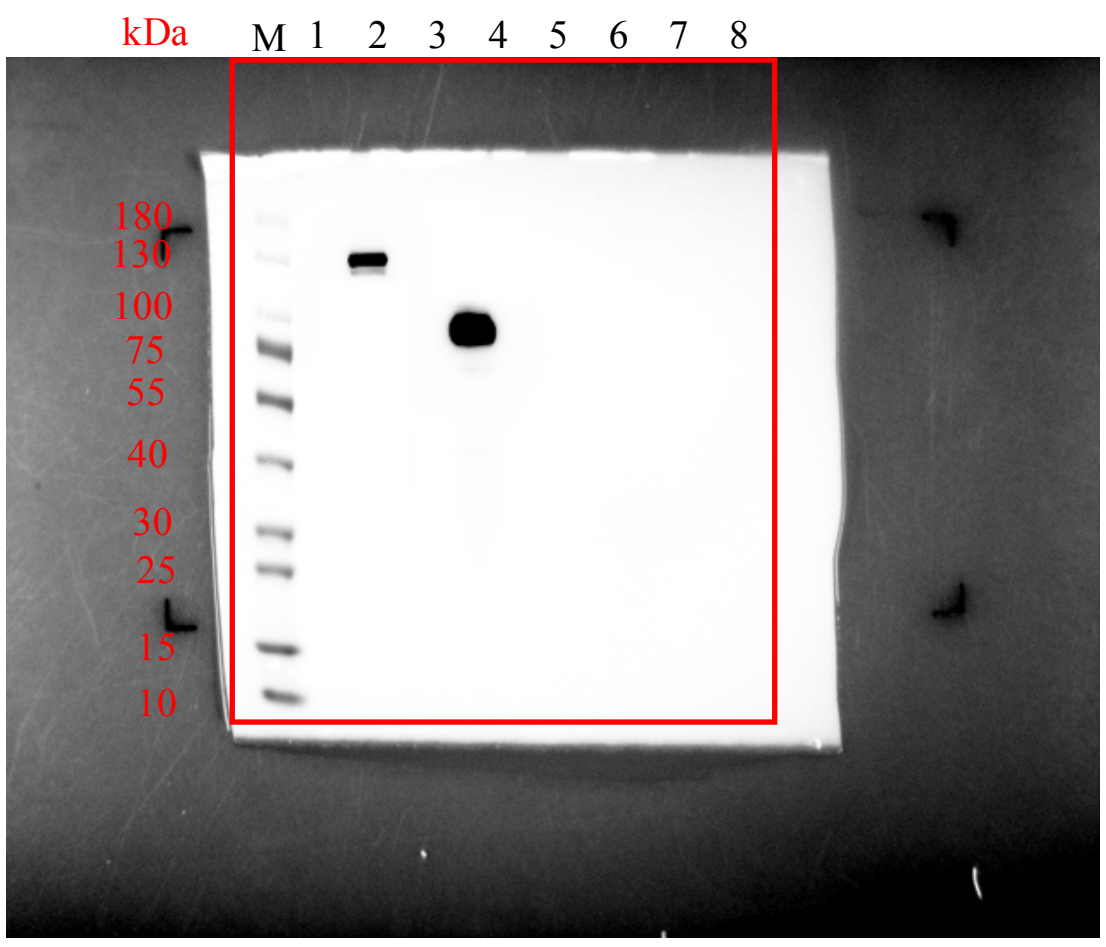

1: Ip (Myc) 293T pCAGGS-GFP+ pCMV14-Myc-N
2: Ip (Myc) 293T pCAGGS-ZC3HAV1+ pCMV14-Myc-N
3: Ip (Myc) 293T pCAGGS-Z1+ pCMV14-Myc-N
4: Ip (Myc) 293T pCAGGS-Z2+ pCMV14-Myc-N
5: Ip (Myc) 293T pCAGGS-Z3+ pCMV14-Myc-N
6: Ip (Myc) 293T pCAGGS-Z4+ pCMV14-Myc-N
7: Ip (Myc) 293T pCAGGS-Z5+ pCMV14-Myc-N
8: Ip (Myc) 293T pCAGGS-Z6+ pCMV14-Myc-N

FIG 5E (panel 1)

Anti-Flag

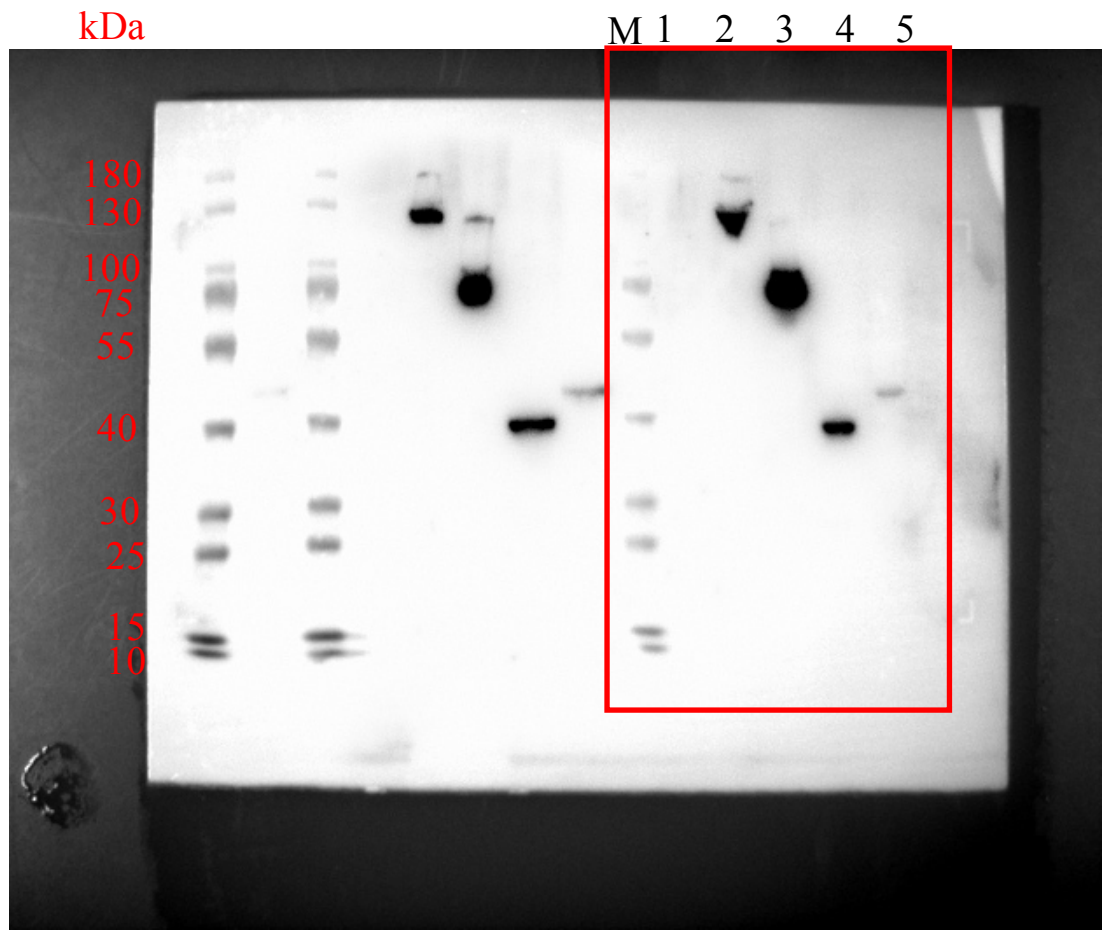

1: Input CRFK pCAGGS-Flag
2: Input CRFK pCAGGS-ZC3HAV1
3: Input CRFK pCAGGS-Z2
4: Input CRFK pCAGGS-Z5
5: Input CRFK pCAGGS-N

FIG 5E (panel 2)

Anti-Flag

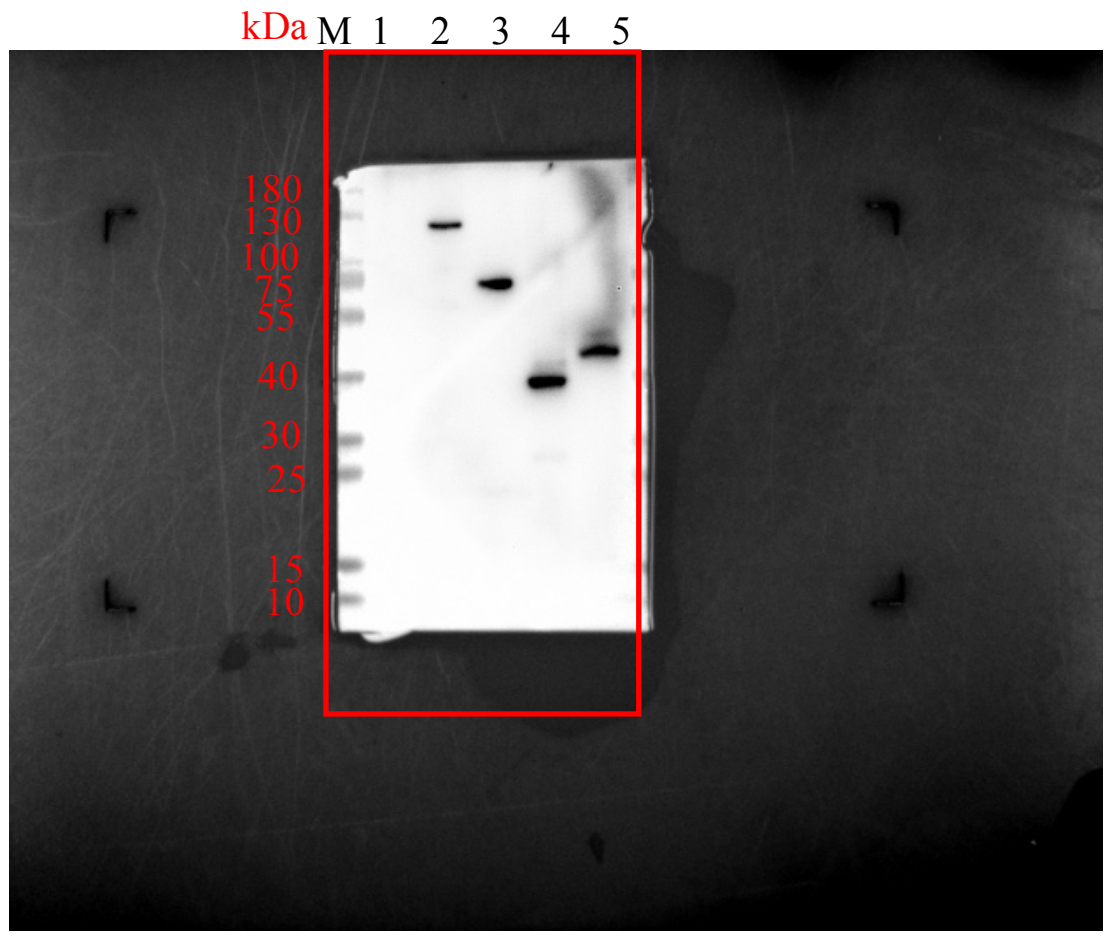

1: Ip (Flag) CRFK pCAGGS-Flag
2: Ip (Flag) CRFK pCAGGS-ZC3HAV1
3: Ip (Flag) CRFK pCAGGS-Z2
4: Ip (Flag) CRFK pCAGGS-Z5
5: Ip (Flag) CRFK pCAGGS-N

FIG 6B (panel 1)

Anti-HA

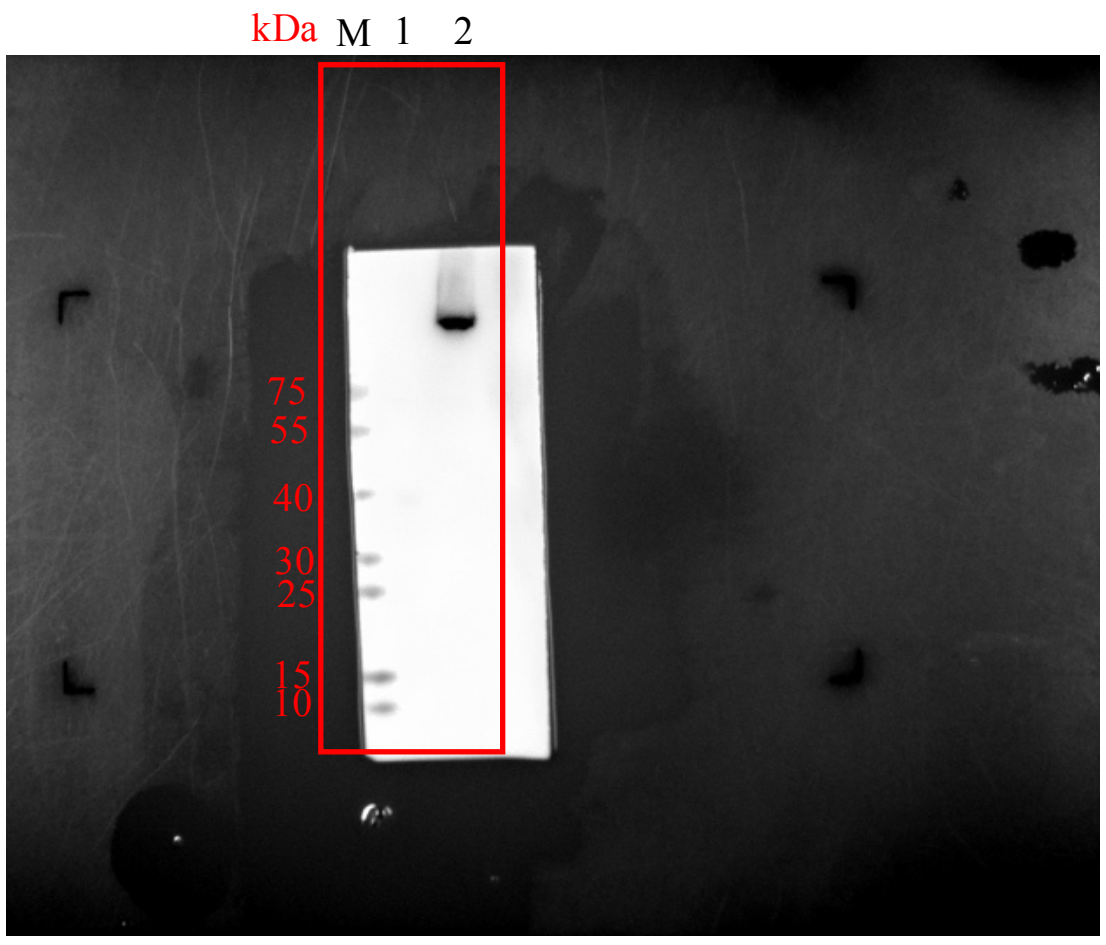

1: Input CRFK pCMV14-Myc-N

2: Input CRFK pCMV14-HA- ZC3HAV1

FIG 6B (panel 2)

Anti-FIPV-N

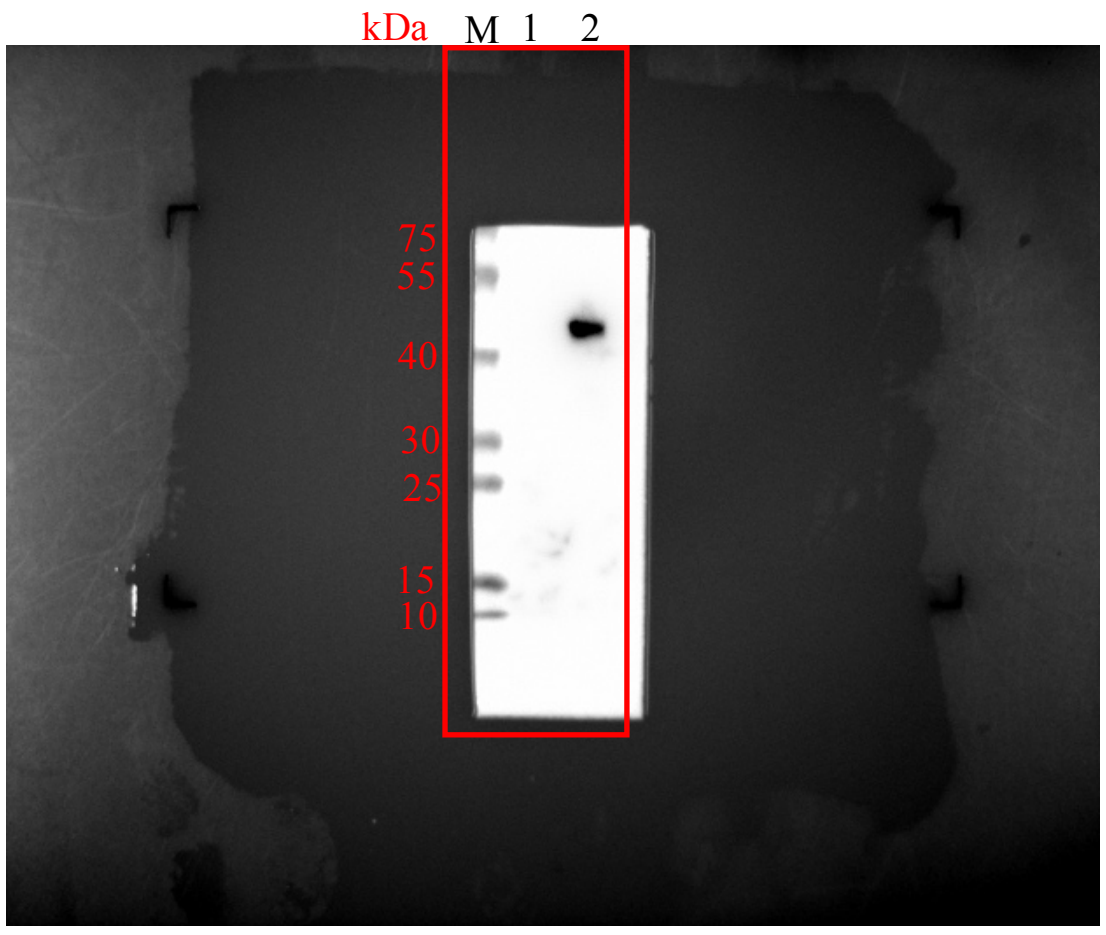

1: Input CRFK pCMV14-HA- ZC3HAV1

2: Input CRFK pCMV14-Myc-N

FIG 6B (panel 3)

Anti-HA

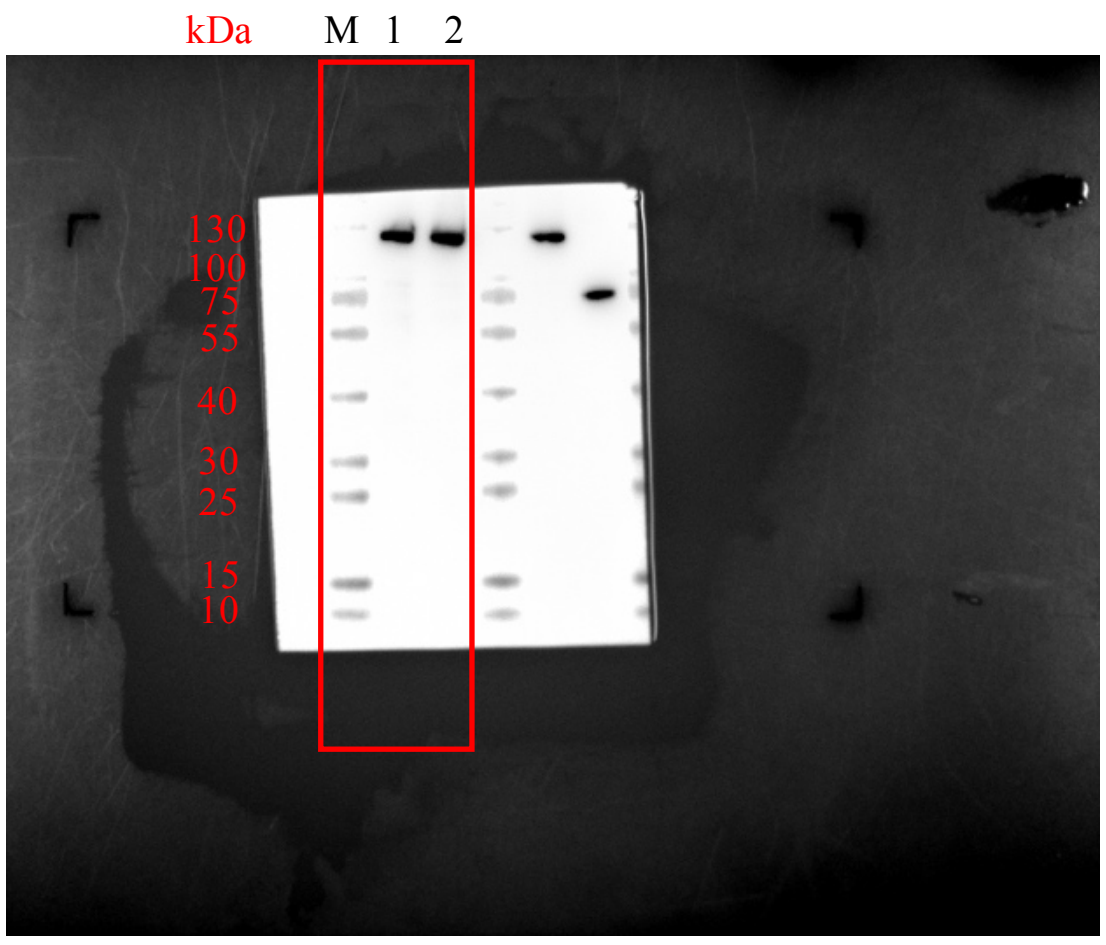

1: Ip (HA) CRFK pCMV14-HA-ZC3HAV1+ pCMV14-Myc

2: Ip (HA) CRFK pCMV14-HA-ZC3HAV1+ pCMV14-Myc-N

FIG 6B (panel 4)

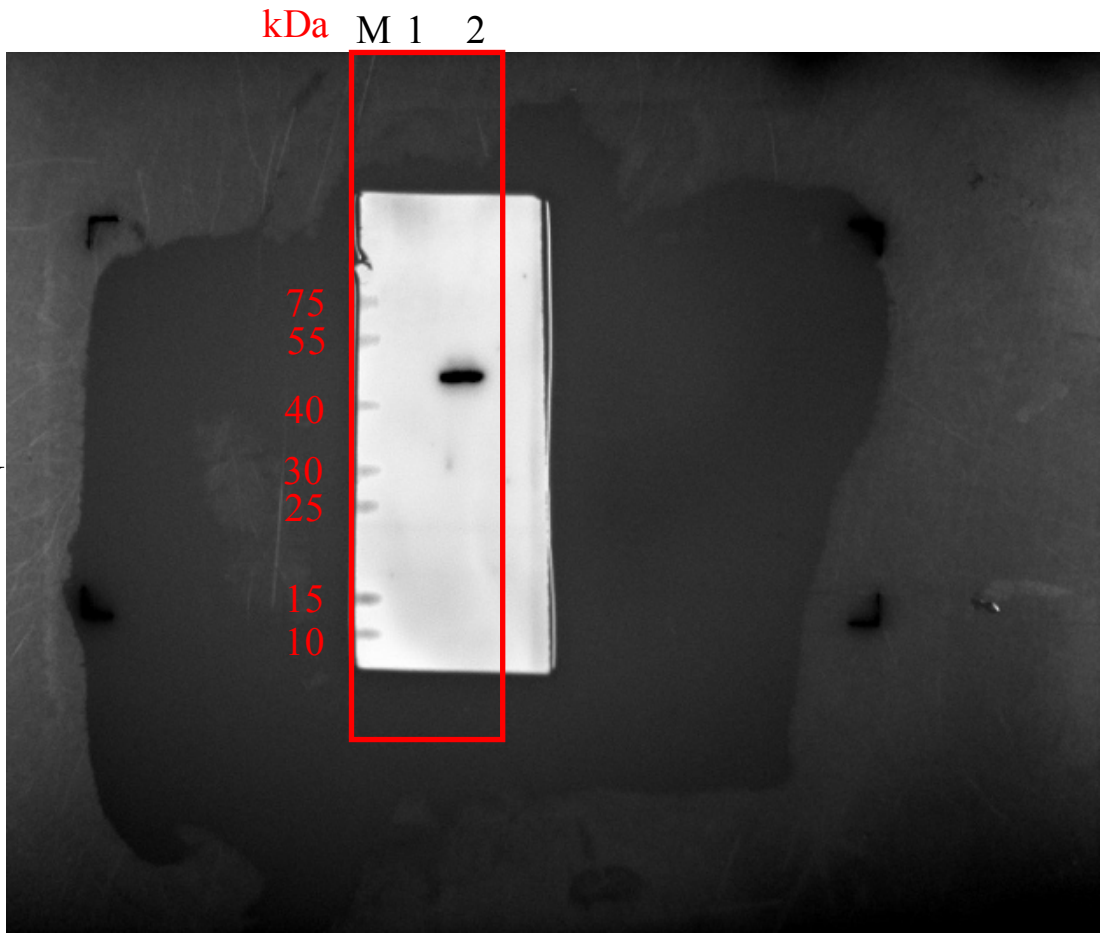

Anti-FIPV-N

1: Ip (HA) CRFK pCMV14-HA-ZC3HAV1+ pCMV14-Myc

2: Ip (HA) CRFK pCMV14-HA-ZC3HAV1+ pCMV14-Myc-N

FIG 6D (panel 1)

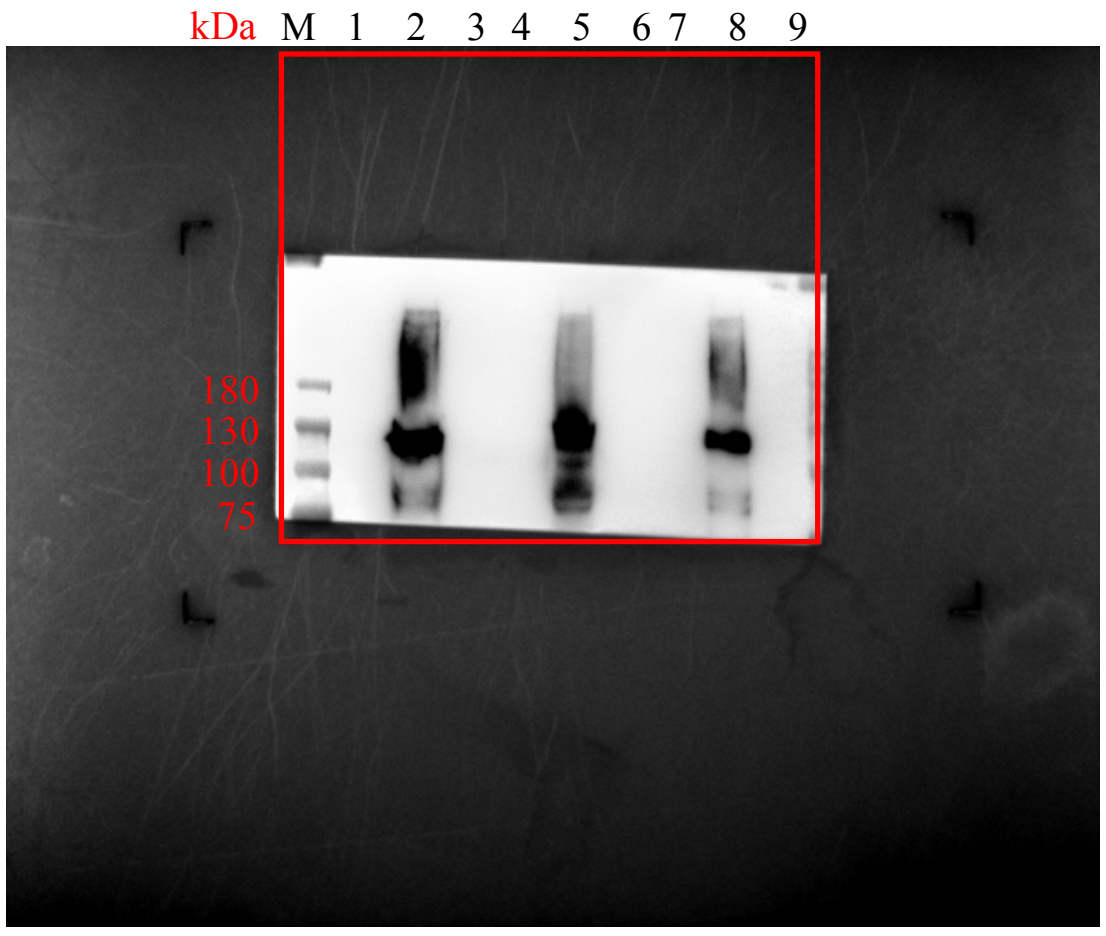

Anti-HA

1: BHK-21 Myc-Vector+JEV 12 hpi

2: BHK-21 HA-ZC3HAV1+JEV 12 hpi

3: BHK-21 Myc-N+JEV 12 hpi

4: BHK-21 Myc-Vector+JEV 24 hpi

5: BHK-21 HA-ZC3HAV1+JEV 24 hpi

6: BHK-21 Myc-N+JEV 24 hpi

7: BHK-21 Myc-Vector+JEV 36 hpi

8: BHK-21 HA-ZC3HAV1+JEV 36 hpi

9: BHK-21 Myc-N+JEV 36 hpi

FIG 6D (panel 2)

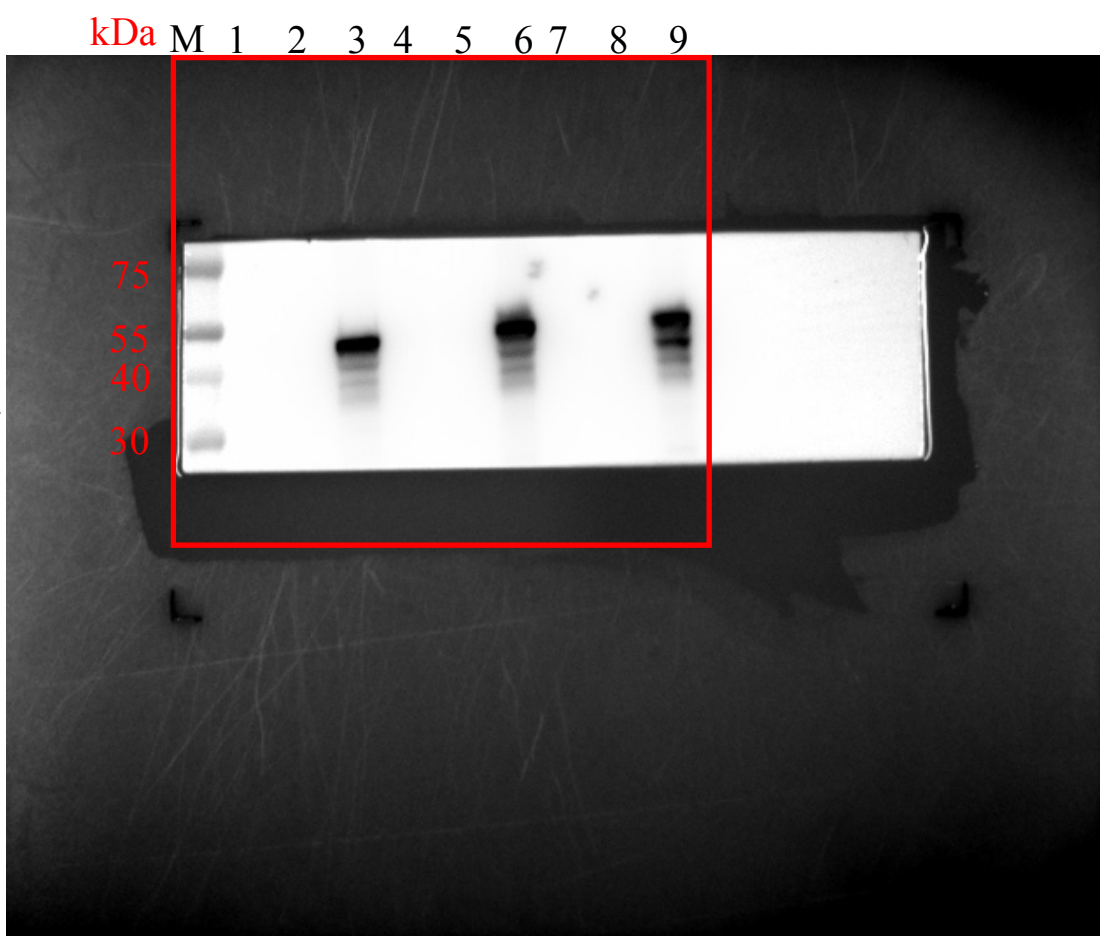

Anti-FIPV N

1: BHK-21 Myc-Vector+JEV 12 hpi

2: BHK-21 HA-ZC3HAV1+JEV 12 hpi

3: BHK-21 Myc-N+JEV 12 hpi

4: BHK-21 Myc-Vector+JEV 24 hpi

5: BHK-21 HA-ZC3HAV1+JEV 24 hpi

6: BHK-21 Myc-N+JEV 24 hpi

7: BHK-21 Myc-Vector+JEV 36 hpi

8: BHK-21 HA-ZC3HAV1+JEV 36 hpi

9: BHK-21 Myc-N+JEV 36 hpi

FIG 6D (panel 3)

Anti-JEV NS1

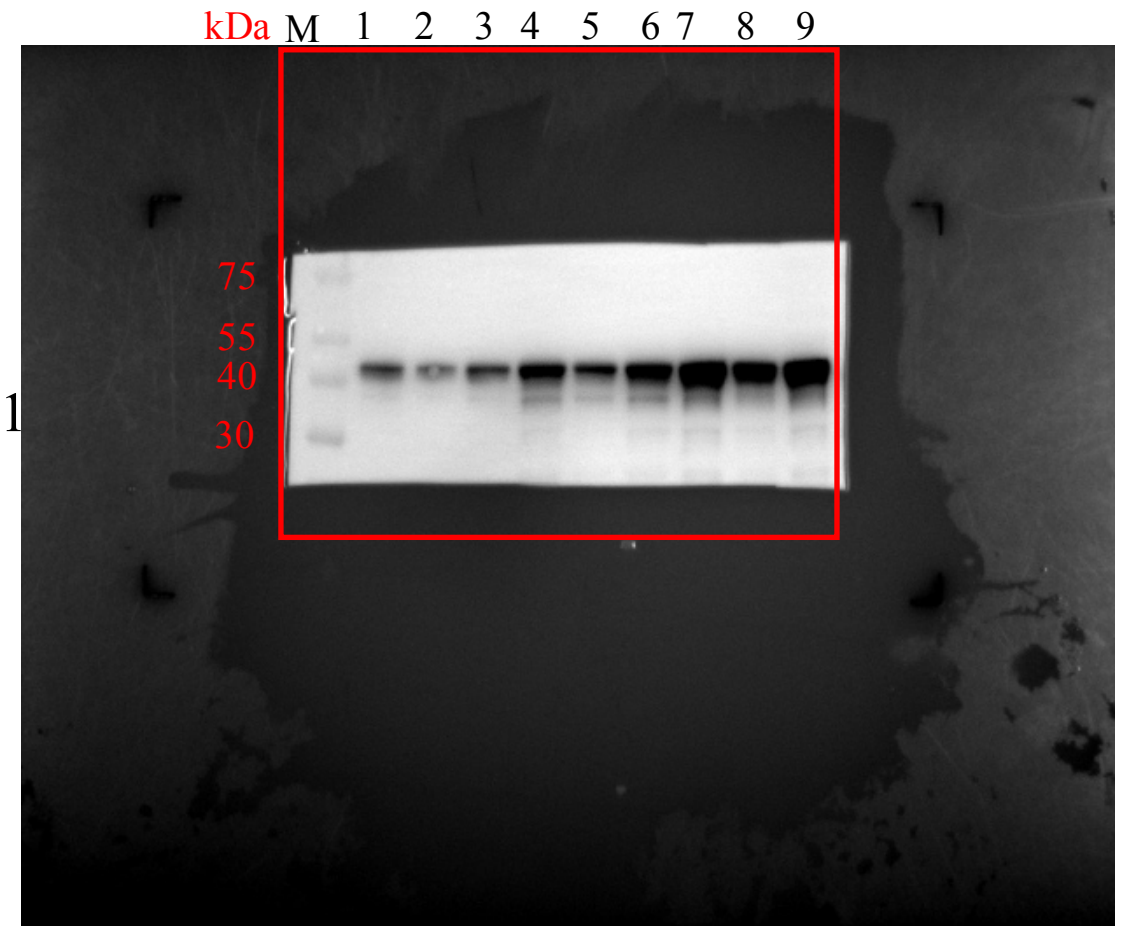

1: BHK-21 Myc-Vector+JEV 12 hpi
2: BHK-21 HA-ZC3HAV1+JEV 12 hpi
3: BHK-21 Myc-N+JEV 12 hpi
4: BHK-21 Myc-Vector+JEV 24 hpi
5: BHK-21 HA-ZC3HAV1+JEV 24 hpi
6: BHK-21 Myc-N+JEV 24 hpi
7: BHK-21 Myc-Vector+JEV 36 hpi
8: BHK-21 HA-ZC3HAV1+JEV 36 hpi
9: BHK-21 Myc-N+JEV 36 hpi

FIG 6D (panel 4)

Anti-β-actin

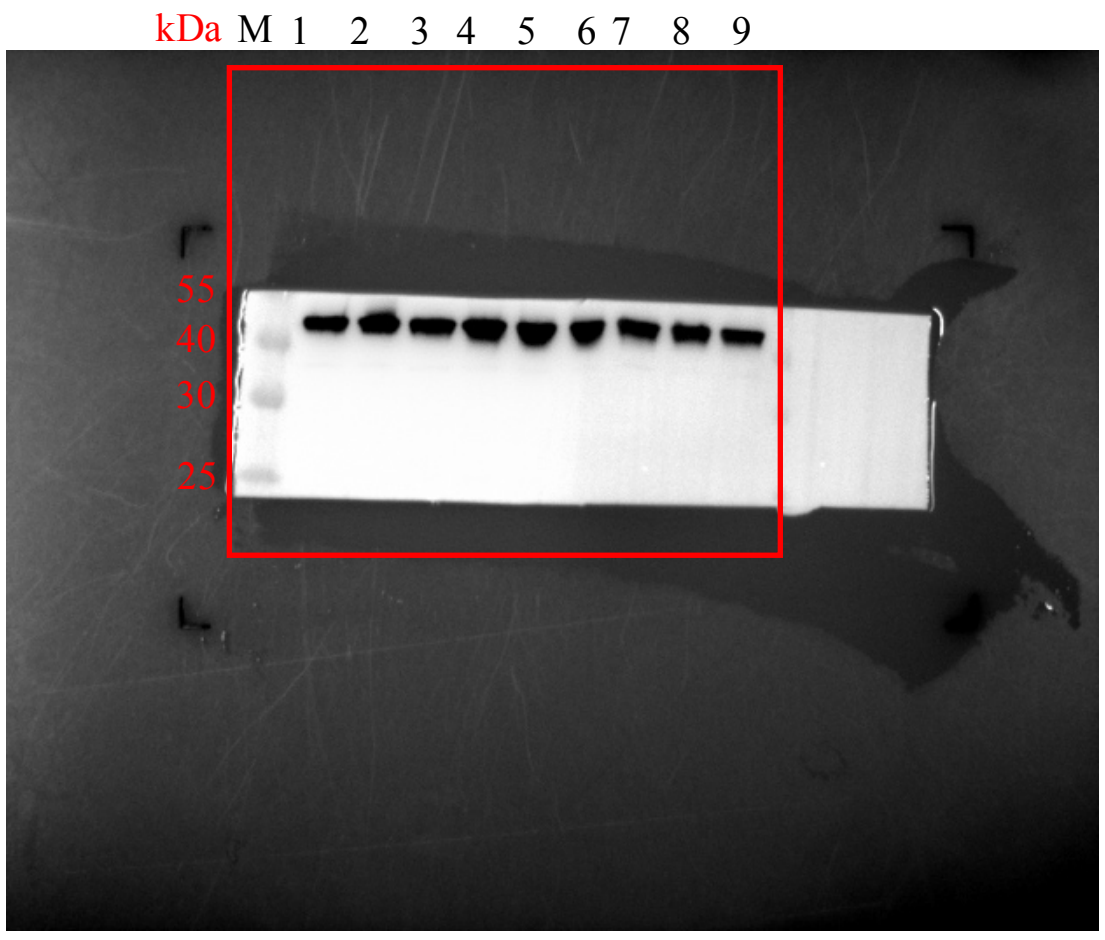

1: BHK-21 Myc-Vector+JEV 12 hpi
2: BHK-21 HA-ZC3HAV1+JEV 12 hpi
3: BHK-21 Myc-N+JEV 12 hpi
4: BHK-21 Myc-Vector+JEV 24 hpi
5: BHK-21 HA-ZC3HAV1+JEV 24 hpi
6: BHK-21 Myc-N+JEV 24 hpi
7: BHK-21 Myc-Vector+JEV 36 hpi
8: BHK-21 HA-ZC3HAV1+JEV 36 hpi
9: BHK-21 Myc-N+JEV 36 hpi

FIG 6F (panel 1)

Anti-HA

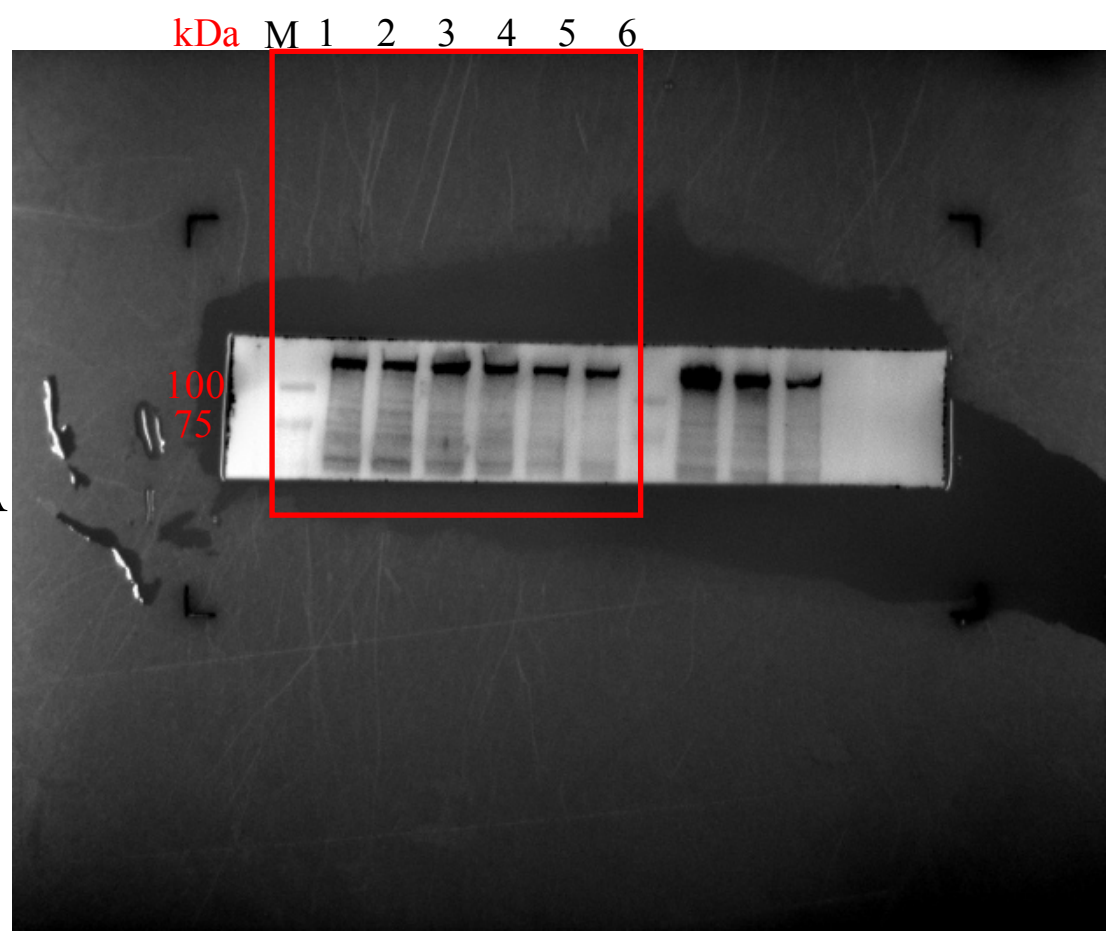

1: BHK-21 Myc-Vector+ HA-ZC3HAV1+JEV 12 hpi
2: BHK-21 Myc-N + HA-ZC3HAV1+JEV 12 hpi
3: BHK-21 Myc-Vector+ HA-ZC3HAV1+JEV 24 hpi
4: BHK-21 Myc-N + HA-ZC3HAV1+JEV 24 hp
5: BHK-21 Myc-Vector+ HA-ZC3HAV1+JEV 36 hpi
6: BHK-21 Myc-N + HA-ZC3HAV1+JEV 36 hpi

FIG 6F (panel 2)

Anti-FIPV N

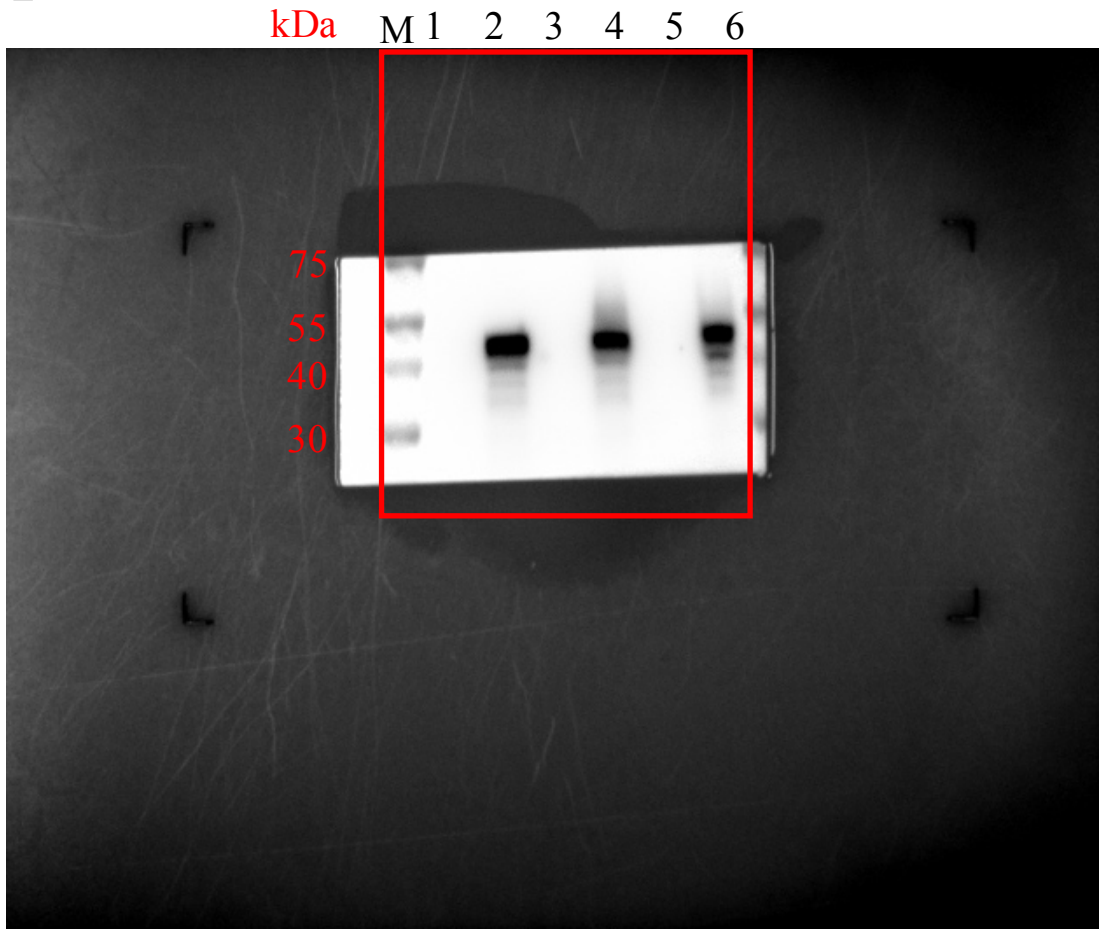

1: BHK-21 Myc-Vector+ HA-ZC3HAV1+JEV 12 hpi
2: BHK-21 Myc-N + HA-ZC3HAV1+JEV 12 hpi
3: BHK-21 Myc-Vector+ HA-ZC3HAV1+JEV 24 hpi
4: BHK-21 Myc-N + HA-ZC3HAV1+JEV 24 hp
5: BHK-21 Myc-Vector+ HA-ZC3HAV1+JEV 36 hpi
6: BHK-21 Myc-N + HA-ZC3HAV1+JEV 36 hpi

FIG 6F (panel 3)

Anti-JEV NS1

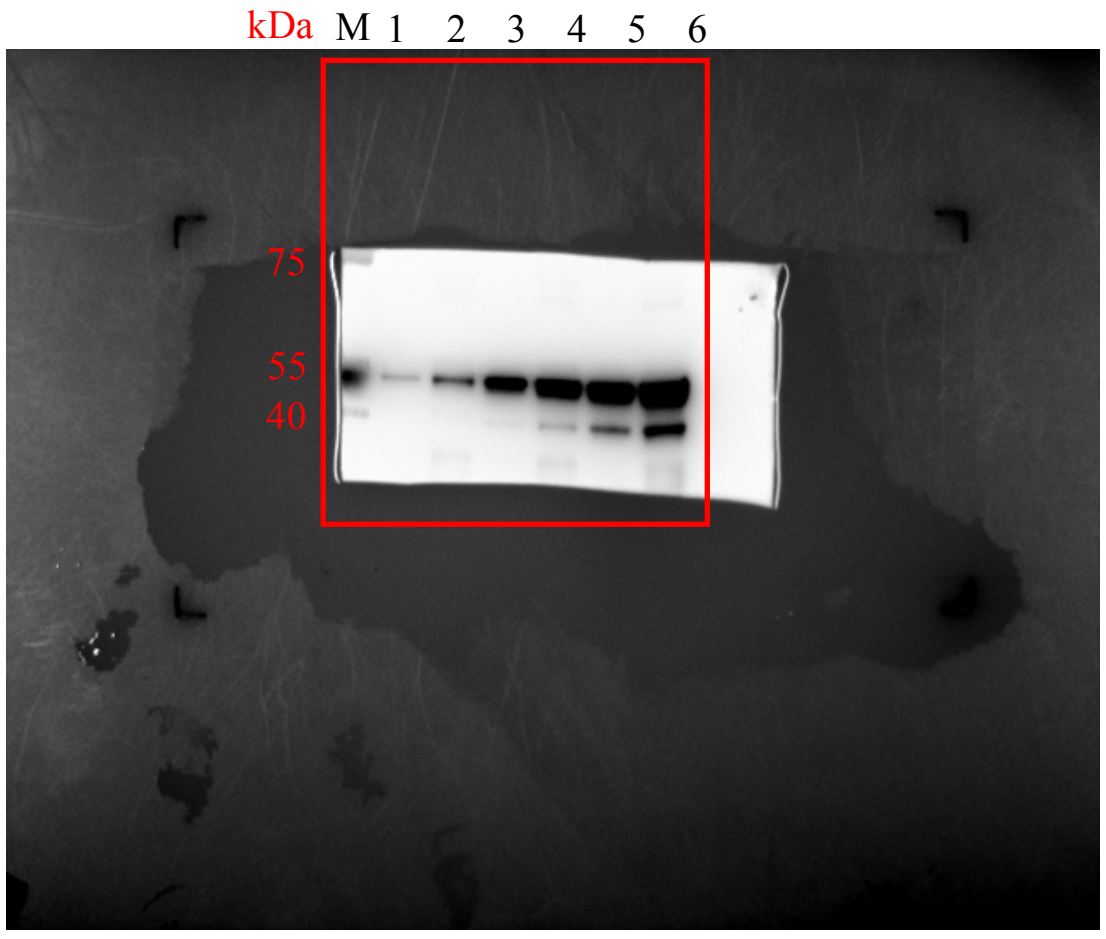

1: BHK-21 Myc-Vector+ HA-ZC3HAV1+JEV 12 hpi
2: BHK-21 Myc-N + HA-ZC3HAV1+JEV 12 hpi
3: BHK-21 Myc-Vector+ HA-ZC3HAV1+JEV 24 hpi
4: BHK-21 Myc-N + HA-ZC3HAV1+JEV 24 hp
5: BHK-21 Myc-Vector+ HA-ZC3HAV1+JEV 36 hpi
6: BHK-21 Myc-N + HA-ZC3HAV1+JEV 36 hpi

FIG 6F (panel 4)

Anti-β-actin

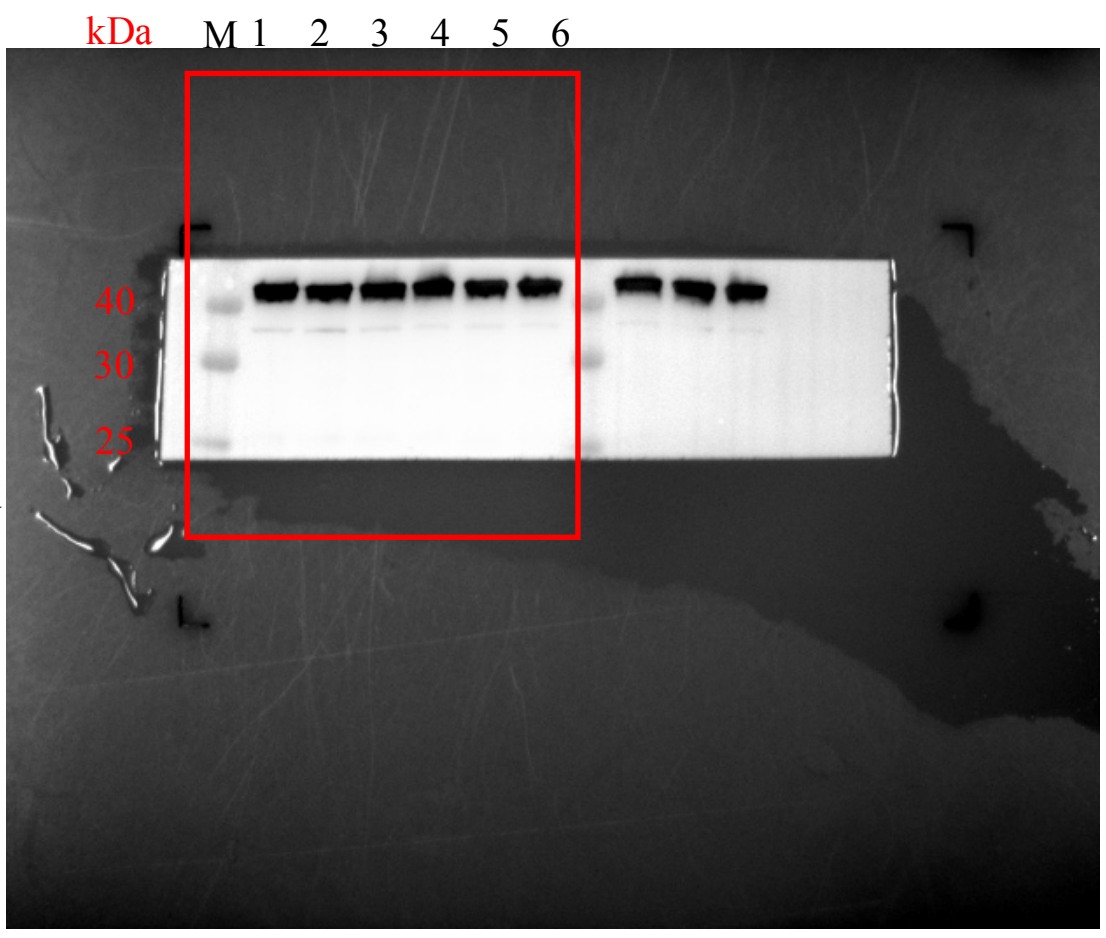

1: BHK-21 Myc-Vector+ HA-ZC3HAV1+JEV 12 hpi
2: BHK-21 Myc-N + HA-ZC3HAV1+JEV 12 hpi
3: BHK-21 Myc-Vector+ HA-ZC3HAV1+JEV 24 hpi
4: BHK-21 Myc-N + HA-ZC3HAV1+JEV 24 hp
5: BHK-21 Myc-Vector+ HA-ZC3HAV1+JEV 36 hpi
6: BHK-21 Myc-N + HA-ZC3HAV1+JEV 36 hpi

S1 FIGA (panel 1)

Anti-HA

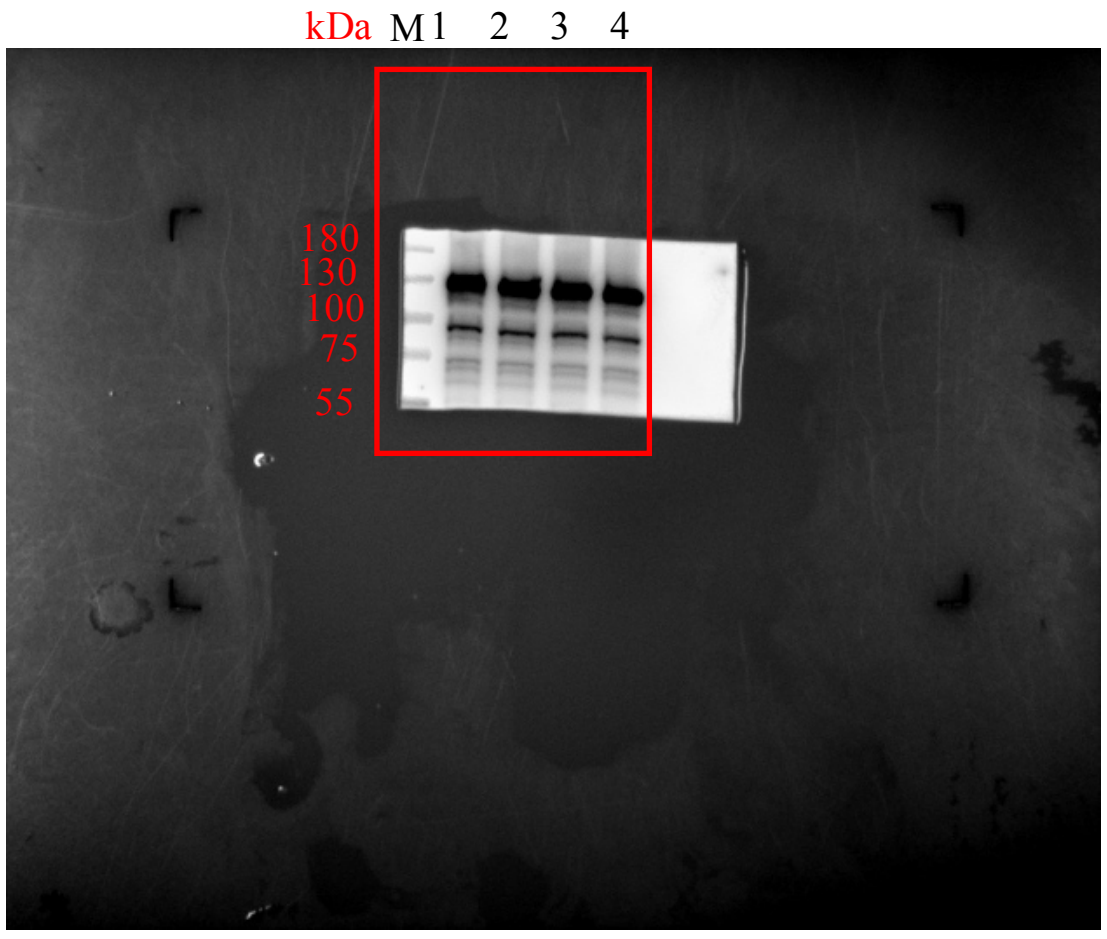

1: CRFK HA-ZC3HAV1 +Flag-Vector
2: CRFK HA-ZC3HAV1+ 1 µg Flag-FIPV N
3: CRFK HA-ZC3HAV1+ 0.5 µg Flag-FIPV N
4: CRFK HA-ZC3HAV1+ 0.25 µg Flag-FIPV N

S1 FIGA (panel 2)

Anti-Flag

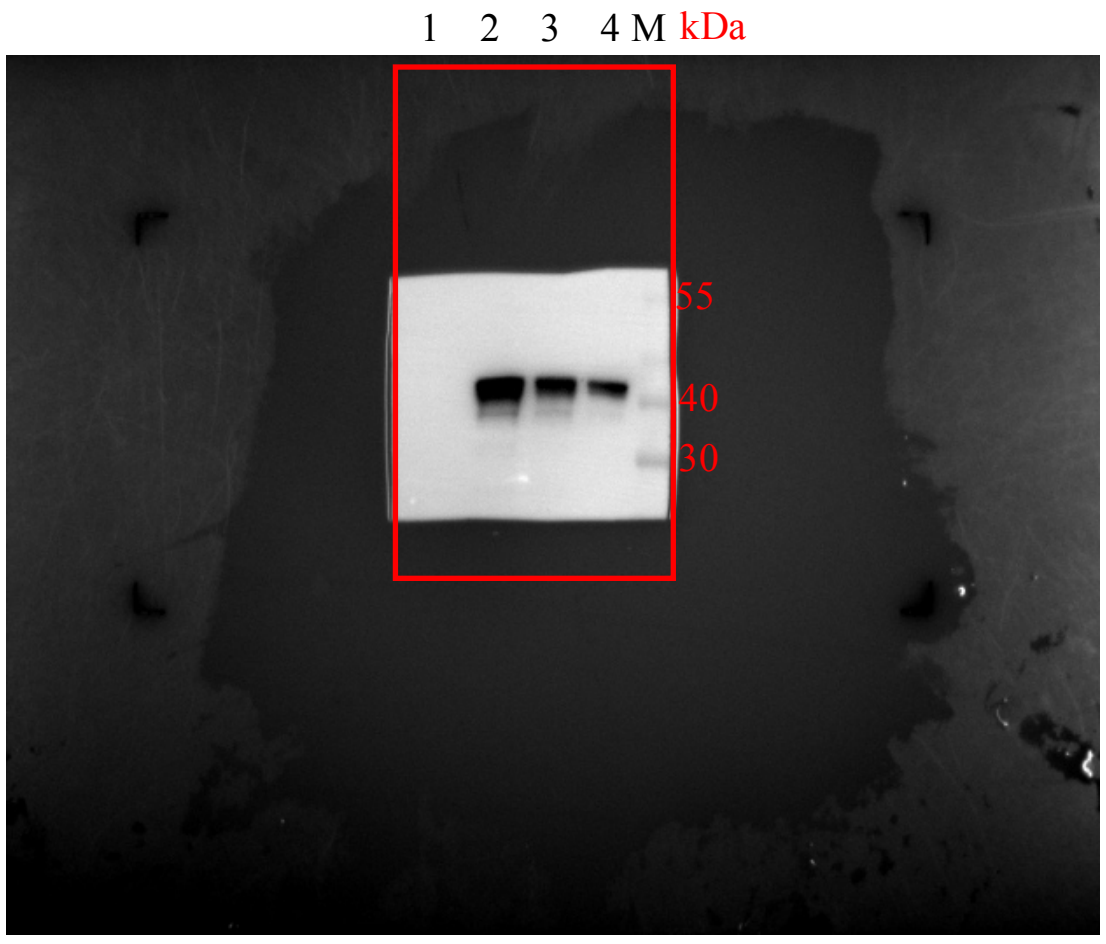

1: CRFK HA-ZC3HAV1 +Flag-Vector
2: CRFK HA-ZC3HAV1+ 1 µg Flag-FIPV N
3: CRFK HA-ZC3HAV1+ 0.5 µg Flag-FIPV N
4: CRFK HA-ZC3HAV1+ 0.25 µg Flag-FIPV N

S1 FIGA (panel 3)

Anti-β-actin

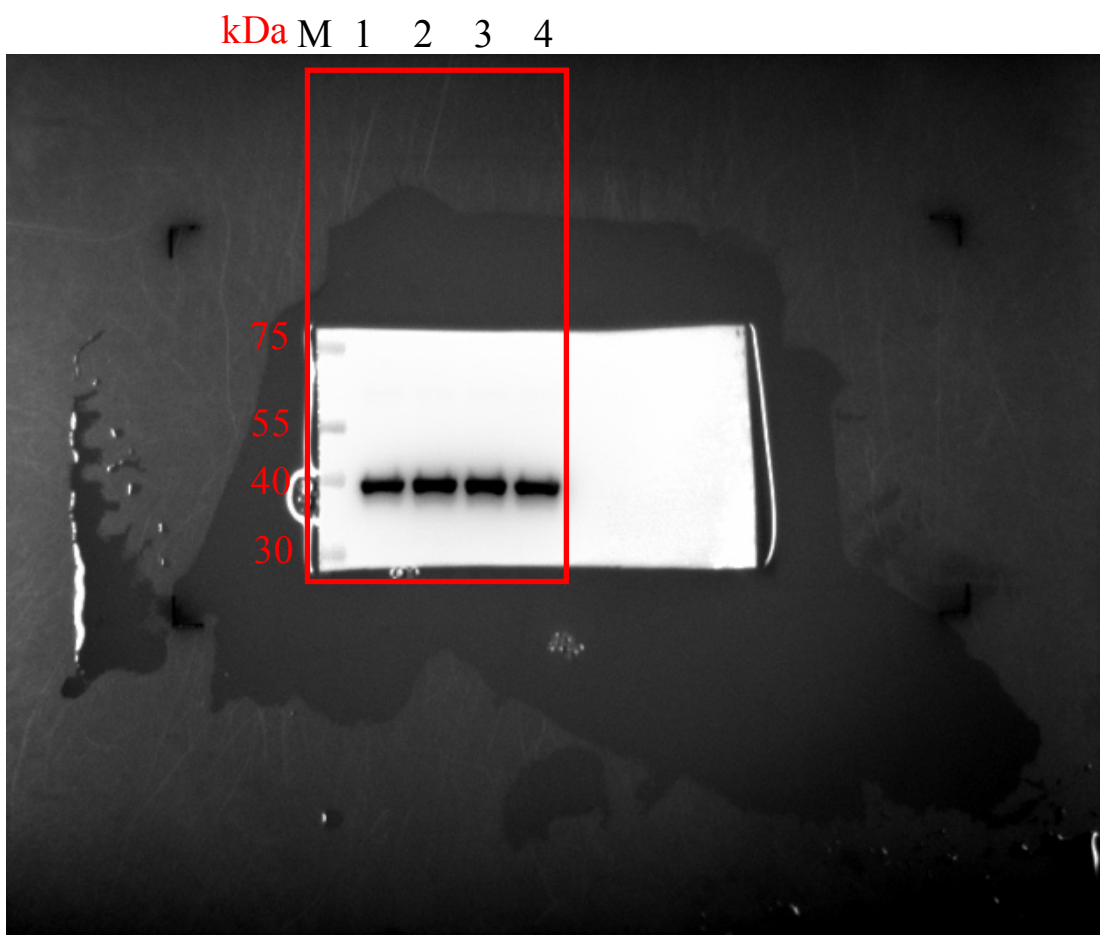

1: CRFK HA-ZC3HAV1 +Flag-Vector
2: CRFK HA-ZC3HAV1+ 1 µg Flag-FIPV N
3: CRFK HA-ZC3HAV1+ 0.5 µg Flag-FIPV N
4: CRFK HA-ZC3HAV1+ 0.25 µg Flag-FIPV N

S1 FIGC (panel 1)

Anti-ZC3HAV1

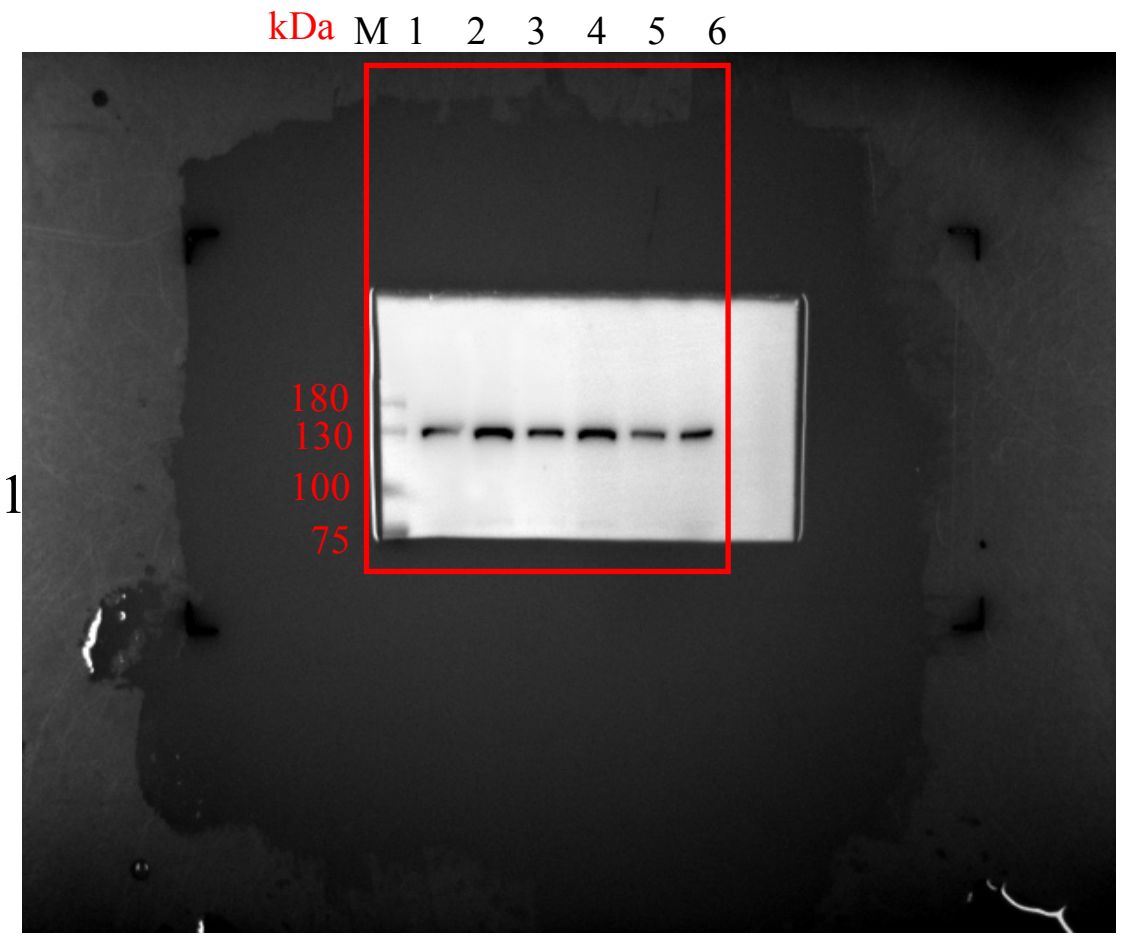

1: CRFK Mock 12 hpi
2: CRFK+FIPV 12 hpi
3: CRFK Mock 16 hpi
4: CRFK+FIPV 16 hpi
5: CRFK Mock 24 hpi
6: CRFK+FIPV 24 hpi

S1 FIGC (panel 2)

Anti-FIPV N

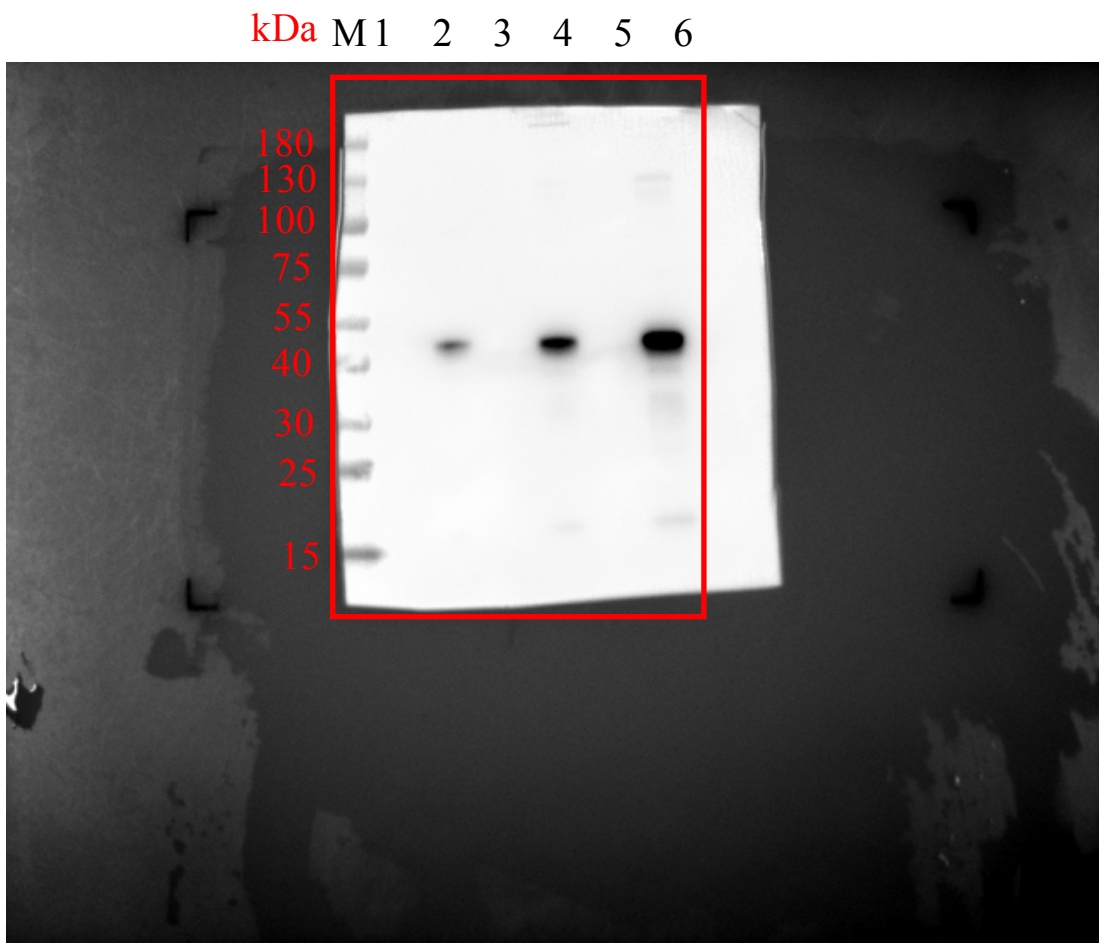

1: CRFK Mock 12 hpi
2: CRFK+FIPV 12 hpi
3: CRFK Mock 16 hpi
4: CRFK+FIPV 16 hpi
5: CRFK Mock 24 hpi
6: CRFK+FIPV 24 hpi

S1 FIGC (panel 3)

Anti-β-actin

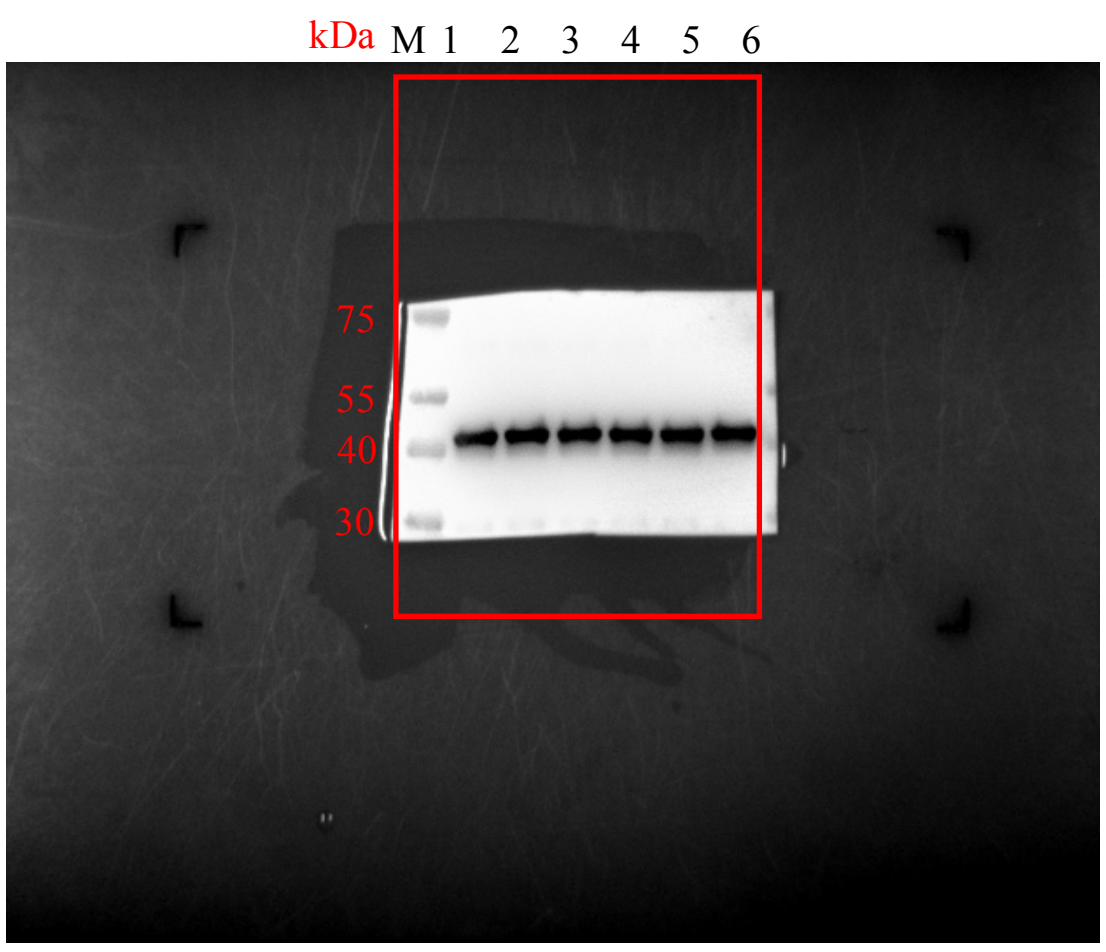

1: CRFK Mock 12 hpi
2: CRFK+FIPV 12 hpi
3: CRFK Mock 16 hpi
4: CRFK+FIPV 16 hpi
5: CRFK Mock 24 hpi
6: CRFK+FIPV 24 hpi

S4 FIGA (panel 1)

Anti-HA

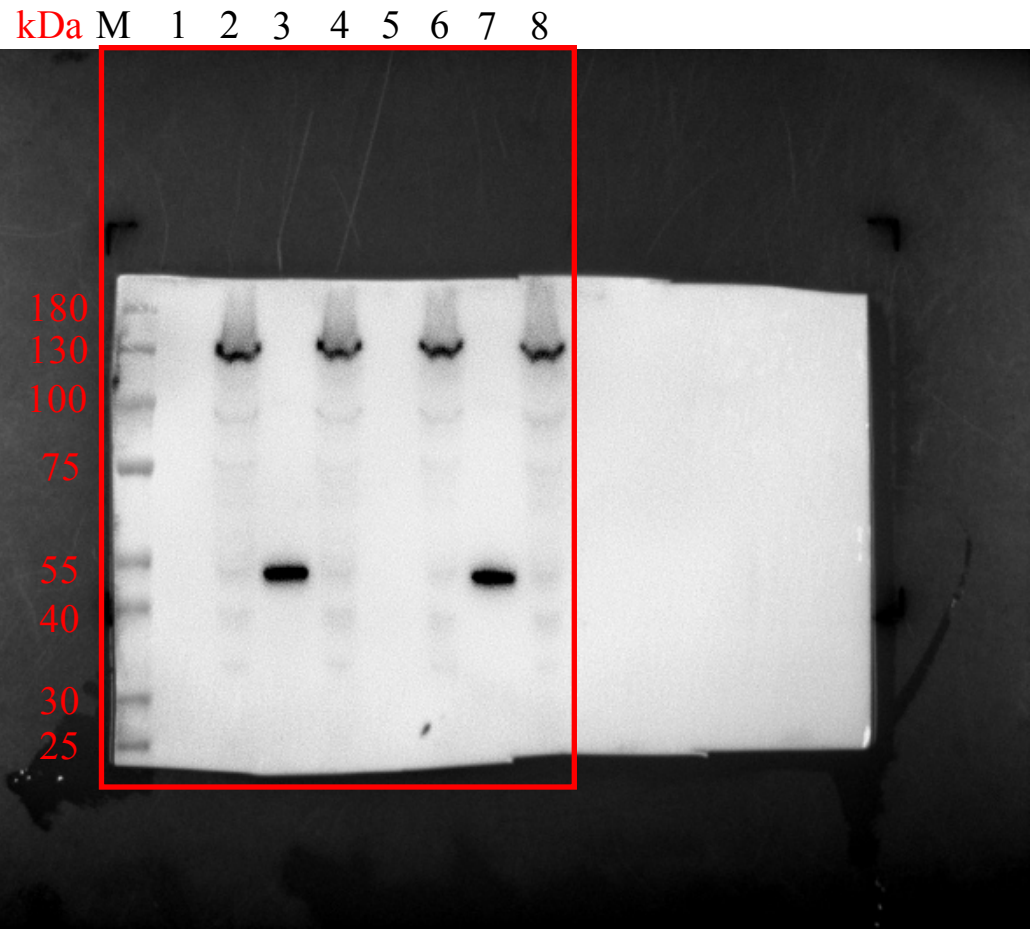

1: CRFK HA-Vector +FIPV 12 hpi
2: CRFK HA-ZC3HAV1+ FIPV 12 hpi
3: CRFK HA-Z5+ FIPV 12 hpi
4: CRFK Myc-N + HA-ZC3HAV1+ FIPV 12 hpi
5: CRFK HA-Vector +FIPV 24 hpi
6: CRFK HA-ZC3HAV1+ FIPV 24 hpi
7: CRFK HA-Z5+ FIPV 24 hpi
8: CRFK Myc-N + HA-ZC3HAV1+ FIPV 24 hpi

S4 FIGA (panel 2)

Anti-Myc

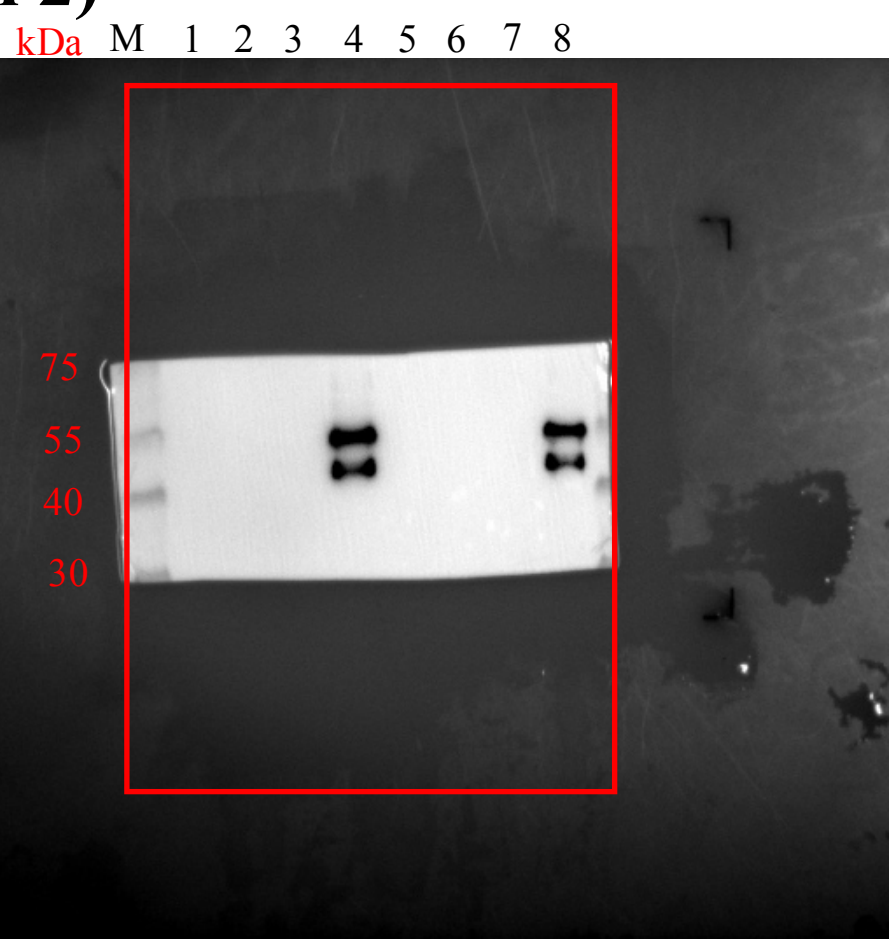

1: CRFK HA-Vector +FIPV 12 hpi
2: CRFK HA-ZC3HAV1+ FIPV 12 hpi
3: CRFK HA-Z5+ FIPV 12 hpi
4: CRFK Myc-N + HA-ZC3HAV1+ FIPV 12 hpi
5: CRFK HA-Vector +FIPV 24 hpi
6: CRFK HA-ZC3HAV1+ FIPV 24 hpi
7: CRFK HA-Z5+ FIPV 24 hpi
8: CRFK Myc-N + HA-ZC3HAV1+ FIPV 24 hpi

S4 FIGA (panel 3)

Anti-FIPV M

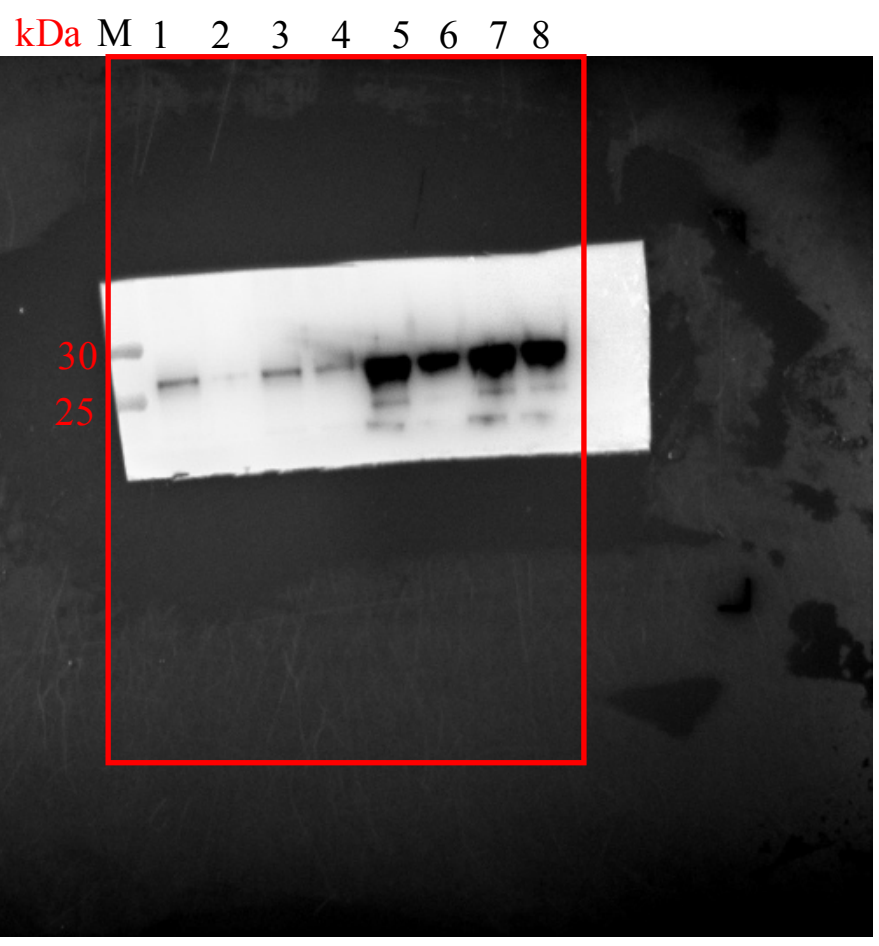

1: CRFK HA-Vector +FIPV 12 hpi
2: CRFK HA-ZC3HAV1+ FIPV 12 hpi
3: CRFK HA-Z5+ FIPV 12 hpi
4: CRFK Myc-N + HA-ZC3HAV1+ FIPV 12 hpi
5: CRFK HA-Vector +FIPV 24 hpi
6: CRFK HA-ZC3HAV1+ FIPV 24 hpi
7: CRFK HA-Z5+ FIPV 24 hpi
8: CRFK Myc-N + HA-ZC3HAV1+ FIPV 24 hpi

S4 FIGA (panel 4)

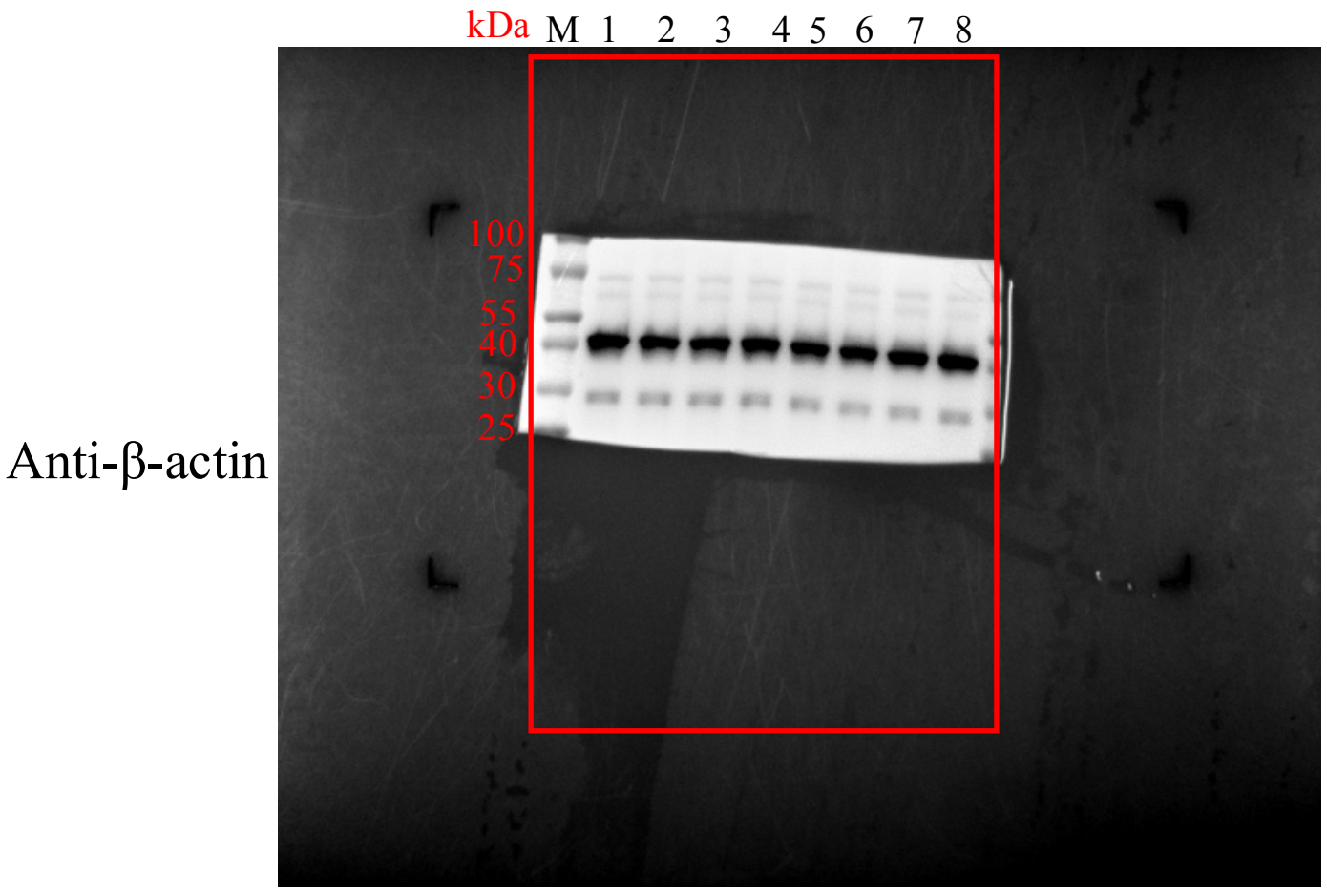

1: CRFK HA-Vector +FIPV 12 hpi
2: CRFK HA-ZC3HAV1+ FIPV 12 hpi
3: CRFK HA-Z5+ FIPV 12 hpi
4: CRFK Myc-N + HA-ZC3HAV1+ FIPV 12 hpi
5: CRFK HA-Vector +FIPV 24 hpi
6: CRFK HA-ZC3HAV1+ FIPV 24 hpi
7: CRFK HA-Z5+ FIPV 24 hpi
8: CRFK Myc-N + HA-ZC3HAV1+ FIPV 24 hpi

S4 FIGD (left panel 1)

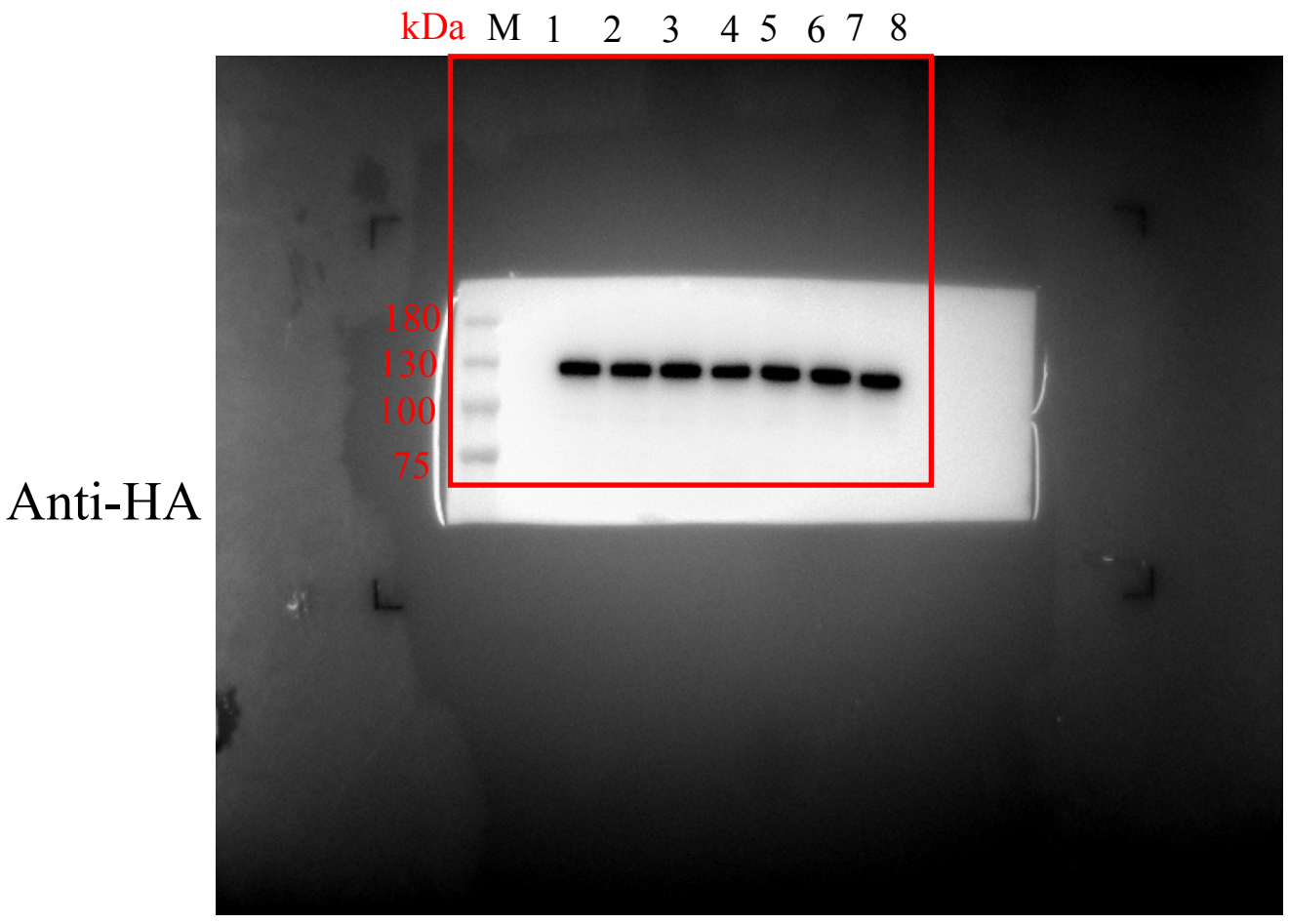

1: CRFK HA-Vector FIPV 12 hpi
2: CRFK HA-ZC3HAV1+Flag-Vector FIPV 12 hpi
3: CRFK HA-ZC3HAV1+pCAGGS-N FIPV 12 hpi
4: CRFK HA-ZC3HAV1+pCAGGS-N1 FIPV 12 hpi
5: CRFK HA-ZC3HAV1+pCAGGS-N2 FIPV 12 hpi
6: CRFK HA-ZC3HAV1+pCAGGS-N3 FIPV 12 hpi
7: CRFK HA-ZC3HAV1+pCAGGS-N4 FIPV 12 hpi
8: CRFK HA-ZC3HAV1+pCAGGS-N5 FIPV 12 hpi

S4 FIGD (right panel 1)

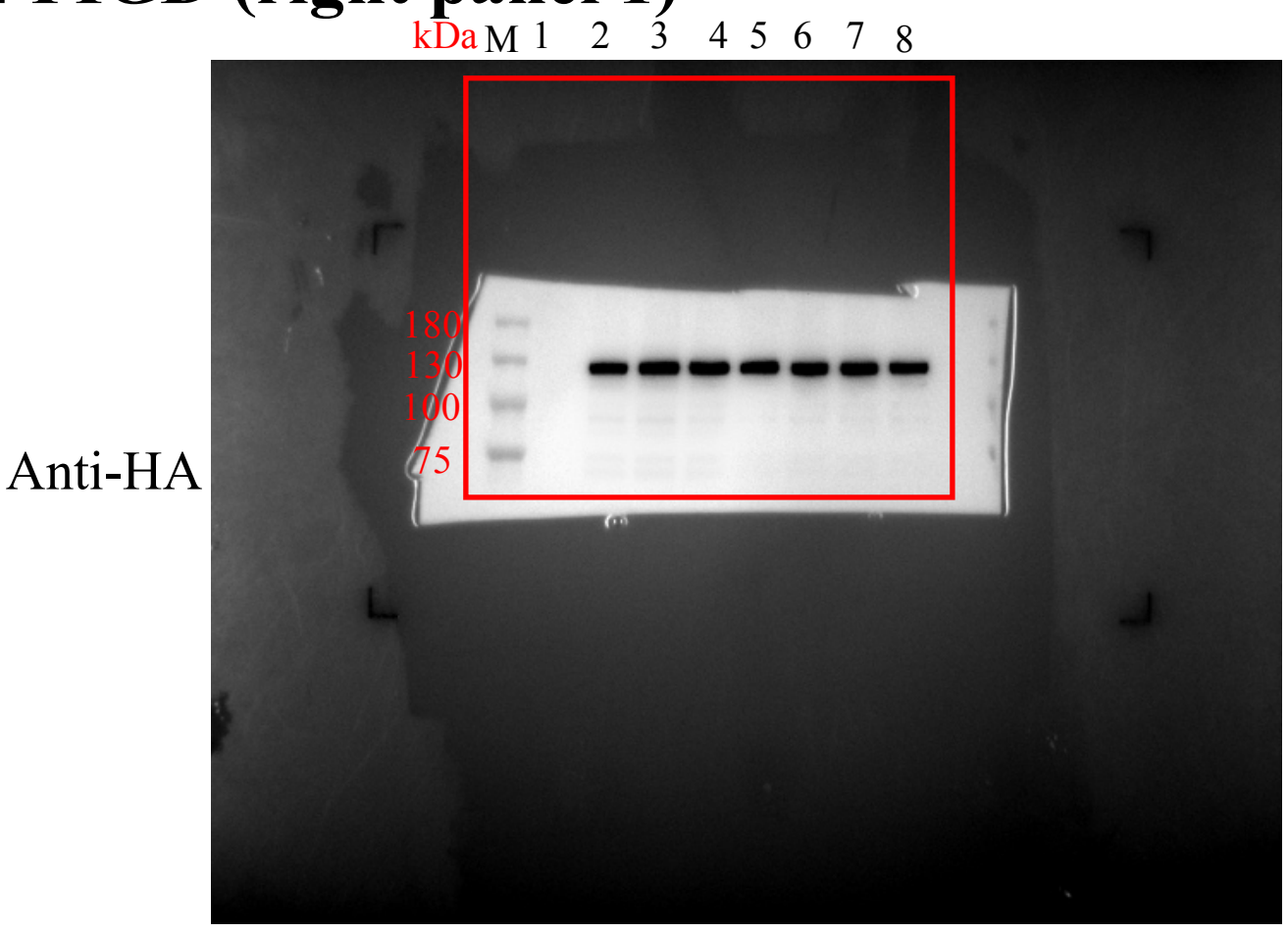

1: CRFK HA-Vector FIPV 24 hpi
2: CRFK HA-ZC3HAV1+Flag-Vector FIPV 24 hpi
3: CRFK HA-ZC3HAV1+pCAGGS-N FIPV 24 hpi
4: CRFK HA-ZC3HAV1+pCAGGS-N1 FIPV 24 hpi
5: CRFK HA-ZC3HAV1+pCAGGS-N2 FIPV 24 hpi
6: CRFK HA-ZC3HAV1+pCAGGS-N3 FIPV 24 hpi
7: CRFK HA-ZC3HAV1+pCAGGS-N4 FIPV 24 hpi
8: CRFK HA-ZC3HAV1+pCAGGS-N5 FIPV 24 hpi

S4 FIGD (left panel 2)

Anti-Flag

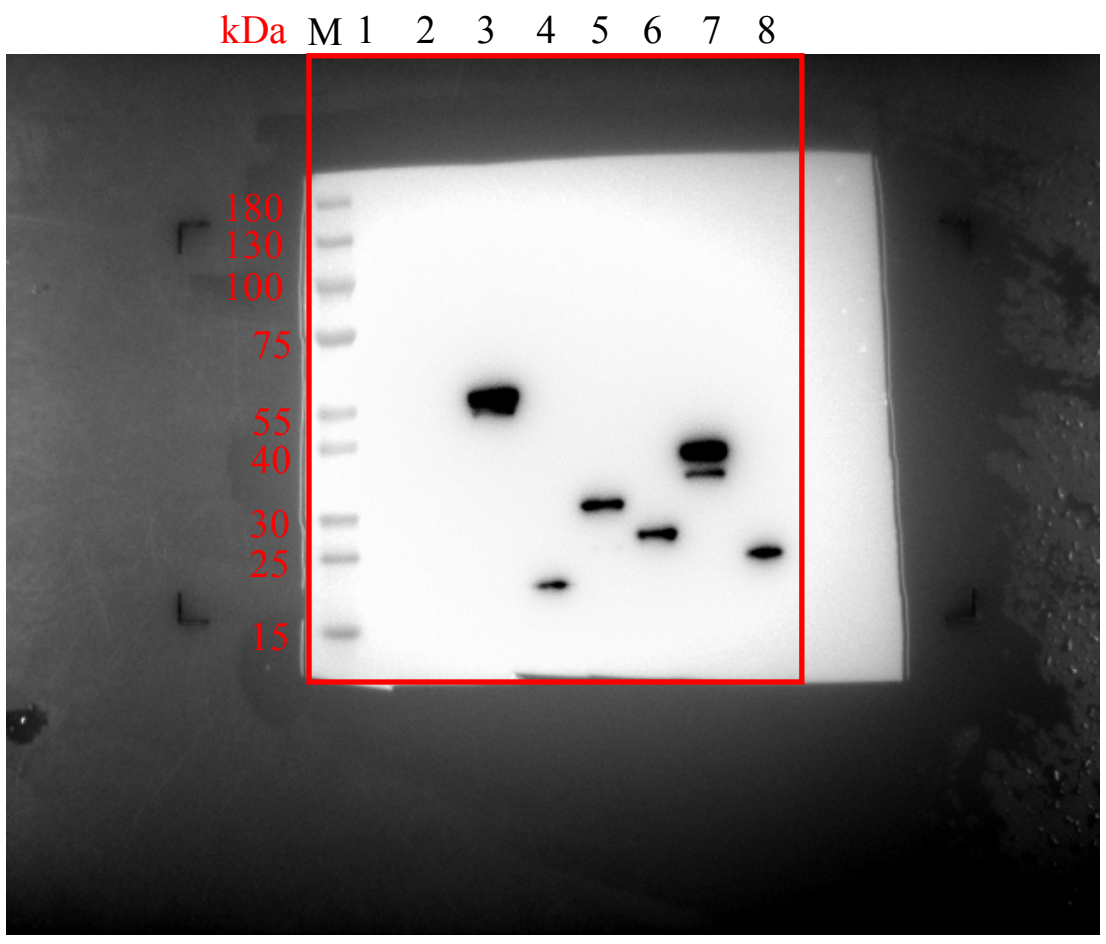

1: CRFK HA-Vector FIPV 12 hpi
2: CRFK HA-ZC3HAV1+Flag-Vector FIPV 12 hpi
3: CRFK HA-ZC3HAV1+pCAGGS-N FIPV 12 hpi
4: CRFK HA-ZC3HAV1+pCAGGS-N1 FIPV 12 hpi
5: CRFK HA-ZC3HAV1+pCAGGS-N2 FIPV 12 hpi
6: CRFK HA-ZC3HAV1+pCAGGS-N3 FIPV 12 hpi
7: CRFK HA-ZC3HAV1+pCAGGS-N4 FIPV 12 hpi
8: CRFK HA-ZC3HAV1+pCAGGS-N5 FIPV 12 hpi

S4 FIGD (right panel 2)

Anti-Flag

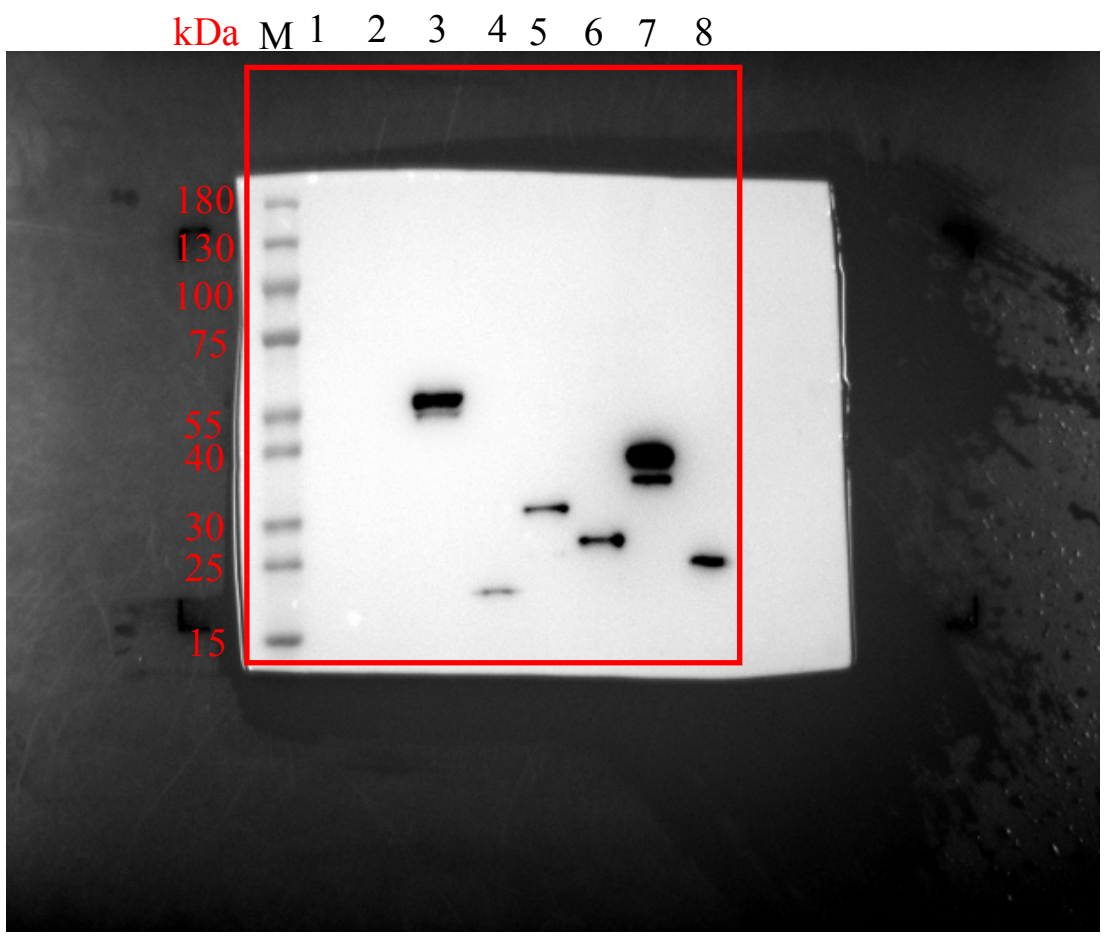

1: CRFK HA-Vector FIPV 24 hpi
2: CRFK HA-ZC3HAV1+Flag-Vector FIPV 24 hpi
3: CRFK HA-ZC3HAV1+pCAGGS-N FIPV 24 hpi
4: CRFK HA-ZC3HAV1+pCAGGS-N1 FIPV 24 hpi
5: CRFK HA-ZC3HAV1+pCAGGS-N2 FIPV 24 hpi
6: CRFK HA-ZC3HAV1+pCAGGS-N3 FIPV 24 hpi
7: CRFK HA-ZC3HAV1+pCAGGS-N4 FIPV 24 hpi
8: CRFK HA-ZC3HAV1+pCAGGS-N5 FIPV 24 hpi

S4 FIGD (left panel 3)

Anti-FIPV M

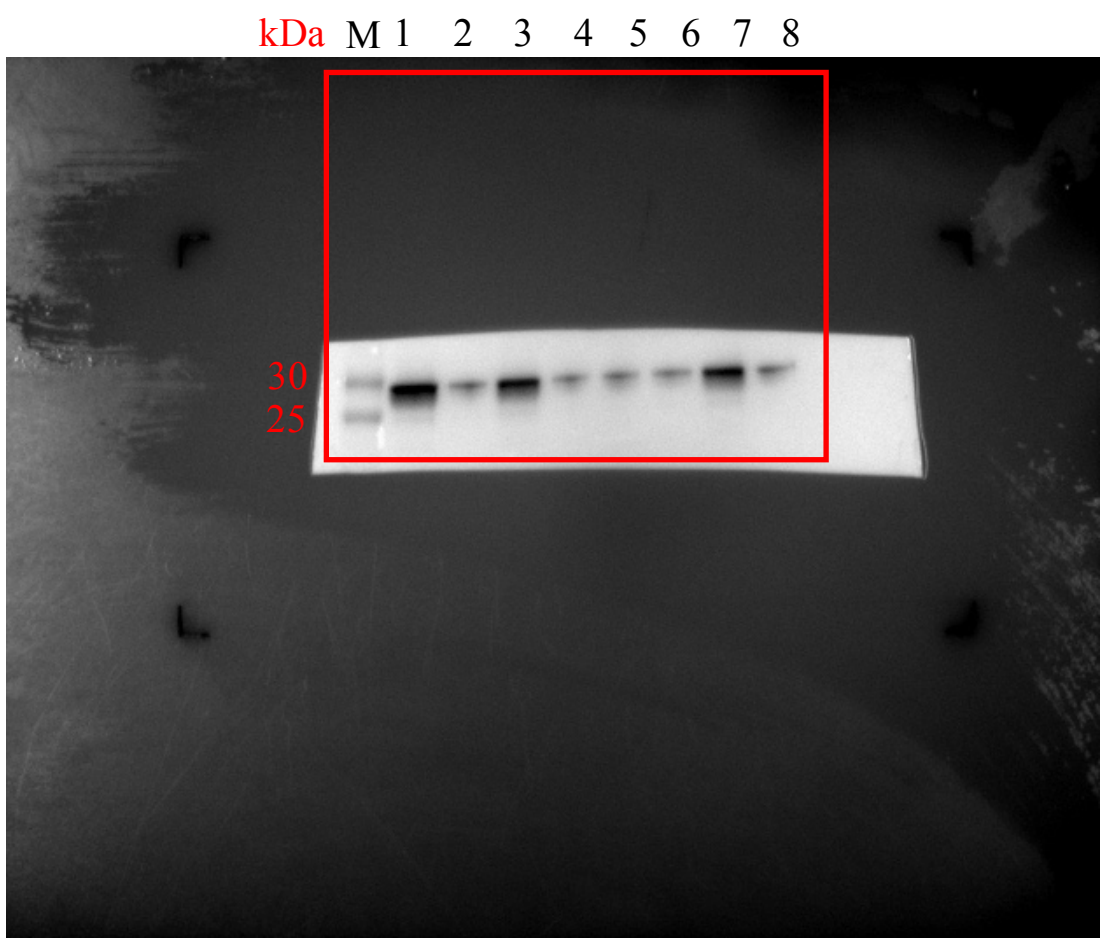

1: CRFK HA-Vector FIPV 12 hpi
2: CRFK HA-ZC3HAV1+Flag-Vector FIPV 12 hpi
3: CRFK HA-ZC3HAV1+pCAGGS-N FIPV 12 hpi
4: CRFK HA-ZC3HAV1+pCAGGS-N1 FIPV 12 hpi
5: CRFK HA-ZC3HAV1+pCAGGS-N2 FIPV 12 hpi
6: CRFK HA-ZC3HAV1+pCAGGS-N3 FIPV 12 hpi
7: CRFK HA-ZC3HAV1+pCAGGS-N4 FIPV 12 hpi
8: CRFK HA-ZC3HAV1+pCAGGS-N5 FIPV 12 hpi

S4 FIGD (right panel 3)

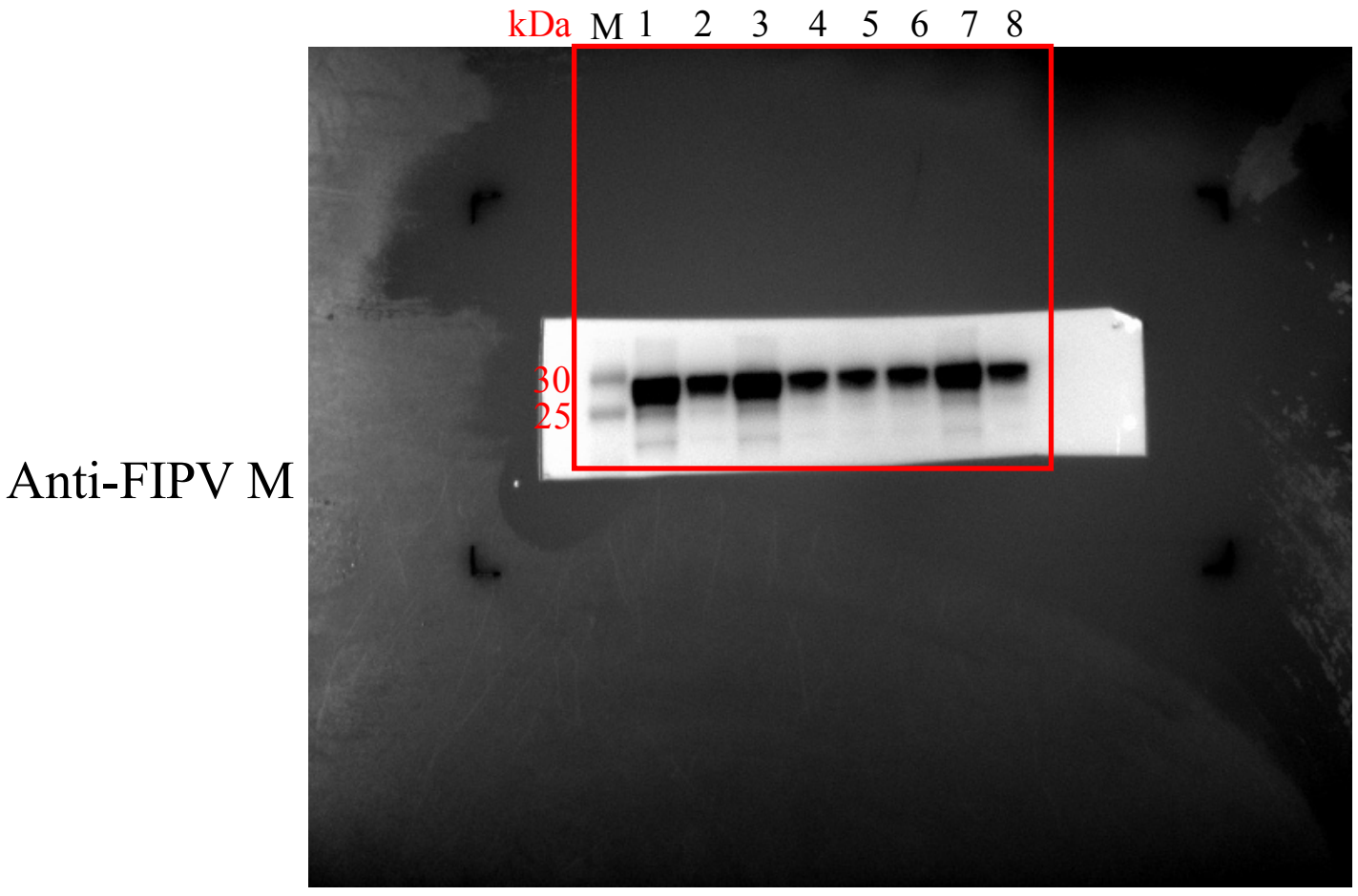

1: CRFK HA-Vector FIPV 24 hpi
2: CRFK HA-ZC3HAV1+Flag-Vector FIPV 24 hpi
3: CRFK HA-ZC3HAV1+pCAGGS-N FIPV 24 hpi
4: CRFK HA-ZC3HAV1+pCAGGS-N1 FIPV 24 hpi
5: CRFK HA-ZC3HAV1+pCAGGS-N2 FIPV 24 hpi
6: CRFK HA-ZC3HAV1+pCAGGS-N3 FIPV 24 hpi
7: CRFK HA-ZC3HAV1+pCAGGS-N4 FIPV 24 hpi
8: CRFK HA-ZC3HAV1+pCAGGS-N5 FIPV 24 hpi

S4 FIGD (left panel 4)

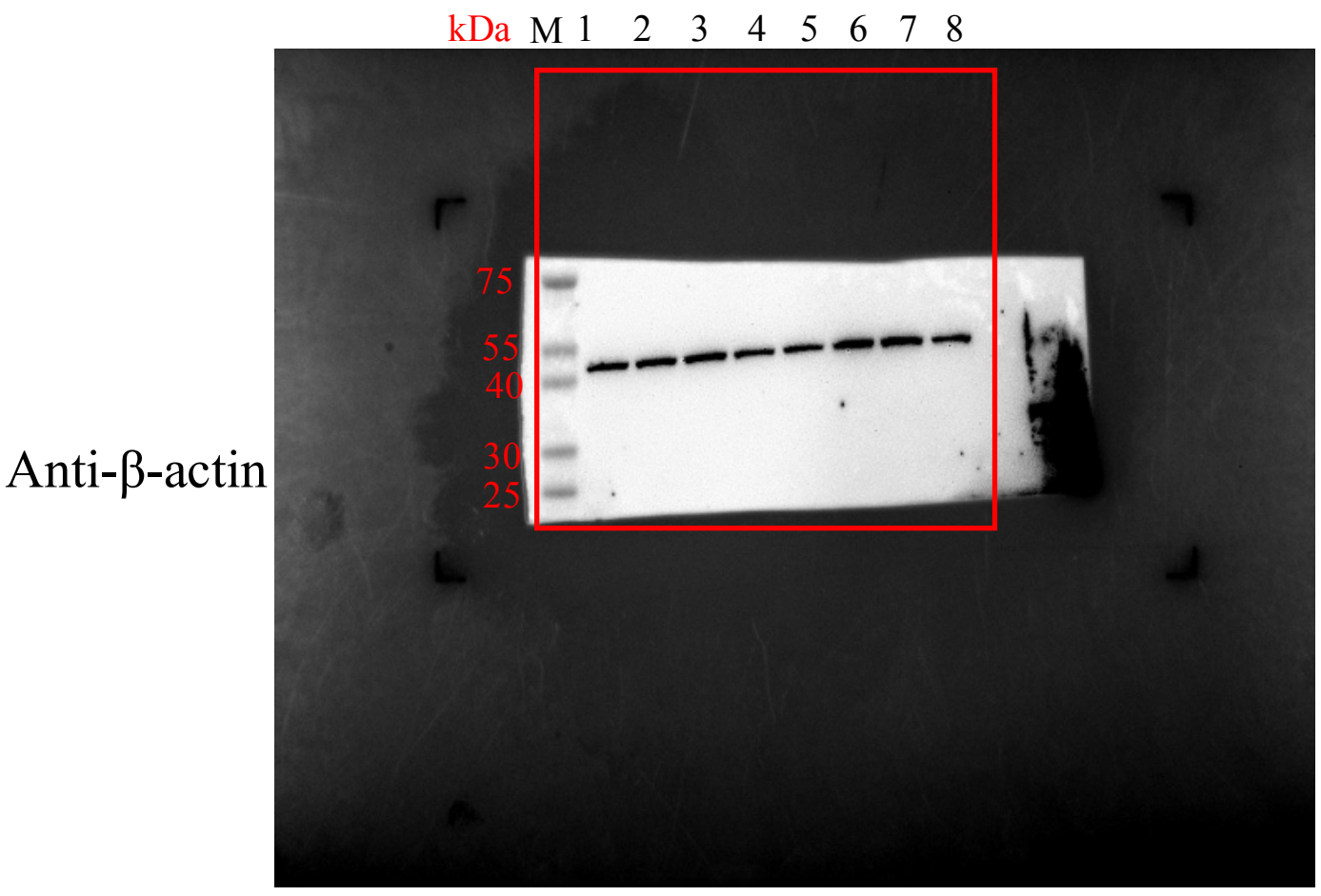

1: CRFK HA-Vector FIPV 12 hpi
2: CRFK HA-ZC3HAV1+Flag-Vector FIPV 12 hpi
3: CRFK HA-ZC3HAV1+pCAGGS-N FIPV 12 hpi
4: CRFK HA-ZC3HAV1+pCAGGS-N1 FIPV 12 hpi
5: CRFK HA-ZC3HAV1+pCAGGS-N2 FIPV 12 hpi
6: CRFK HA-ZC3HAV1+pCAGGS-N3 FIPV 12 hpi
7: CRFK HA-ZC3HAV1+pCAGGS-N4 FIPV 12 hpi
8: CRFK HA-ZC3HAV1+pCAGGS-N5 FIPV 12 hpi

S4 FIGD (right panel 4)

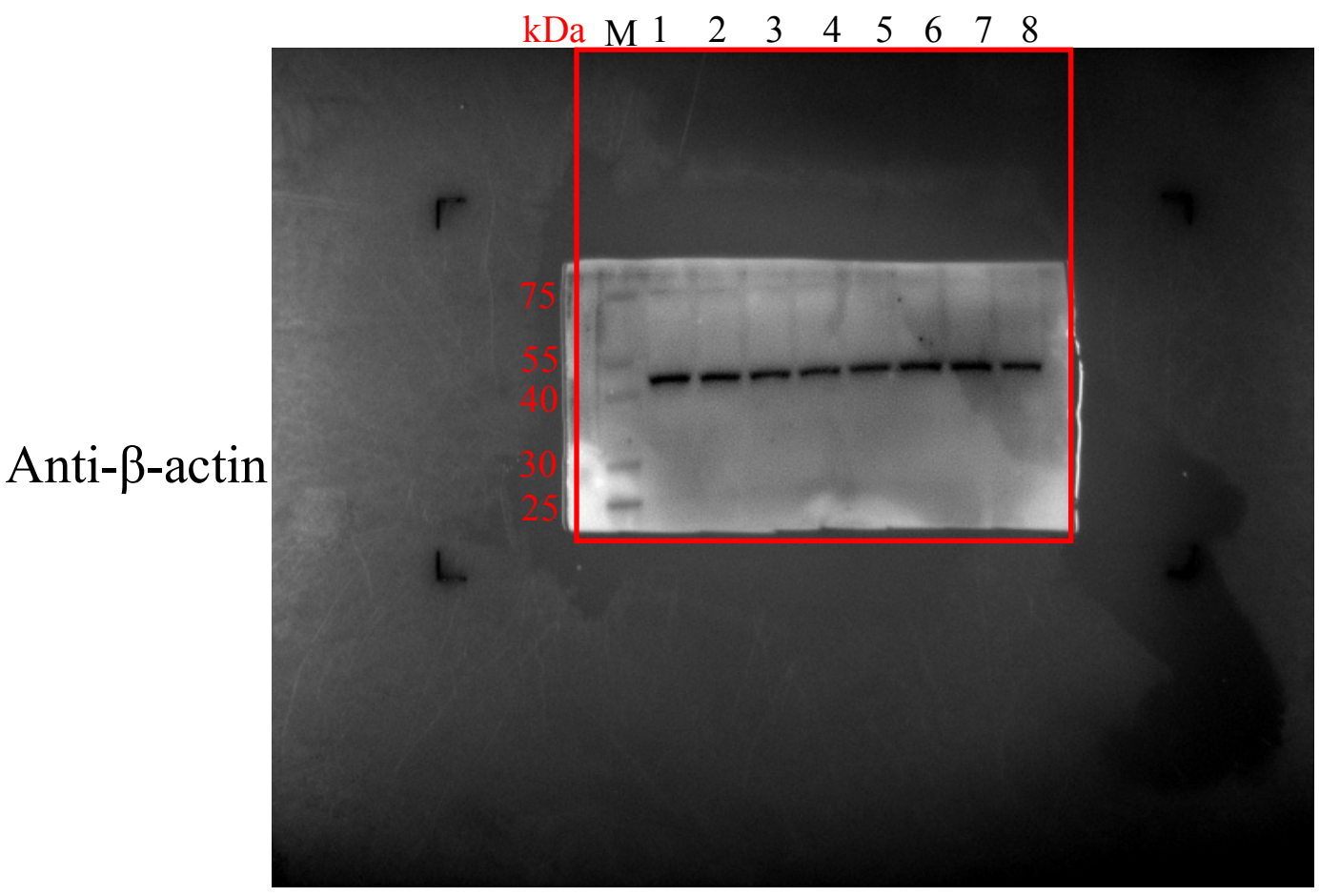

1: CRFK HA-Vector FIPV 24 hpi
2: CRFK HA-ZC3HAV1+Flag-Vector FIPV 24 hpi
3: CRFK HA-ZC3HAV1+pCAGGS-N FIPV 24 hpi
4: CRFK HA-ZC3HAV1+pCAGGS-N1 FIPV 24 hpi
5: CRFK HA-ZC3HAV1+pCAGGS-N2 FIPV 24 hpi
6: CRFK HA-ZC3HAV1+pCAGGS-N3 FIPV 24 hpi
7: CRFK HA-ZC3HAV1+pCAGGS-N4 FIPV 24 hpi
8: CRFK HA-ZC3HAV1+pCAGGS-N5 FIPV 24 hpi

S5 FIGB (panel 1)

kDa M 1 2 3

Anti-HA

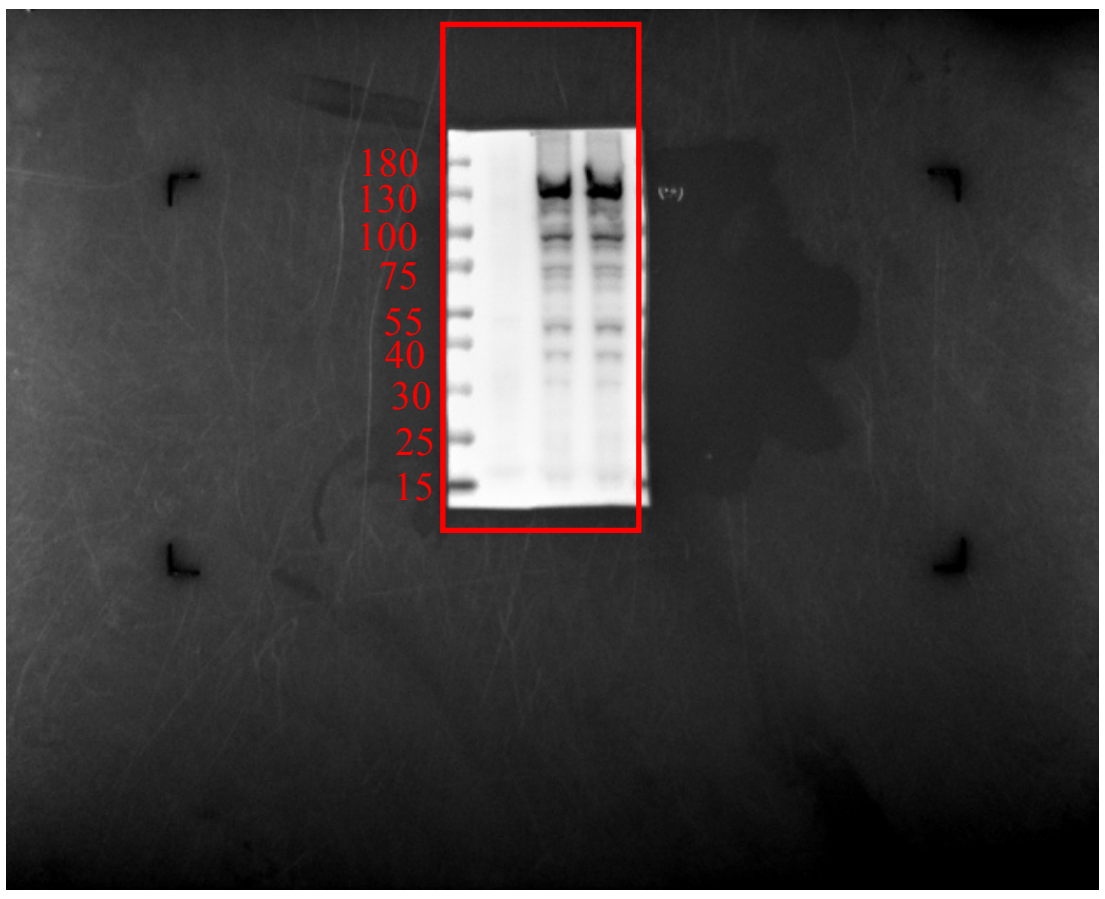

1: Input 293T HA-Vector+Myc-N

2: Input 293T HA-ZC3HAV1+Myc-N

3: Input 293T HA-ZC3HAV1+Myc-Mut-N

S5 FIGB (panel 2)

kDa M 1 2 3

Anti-Myc

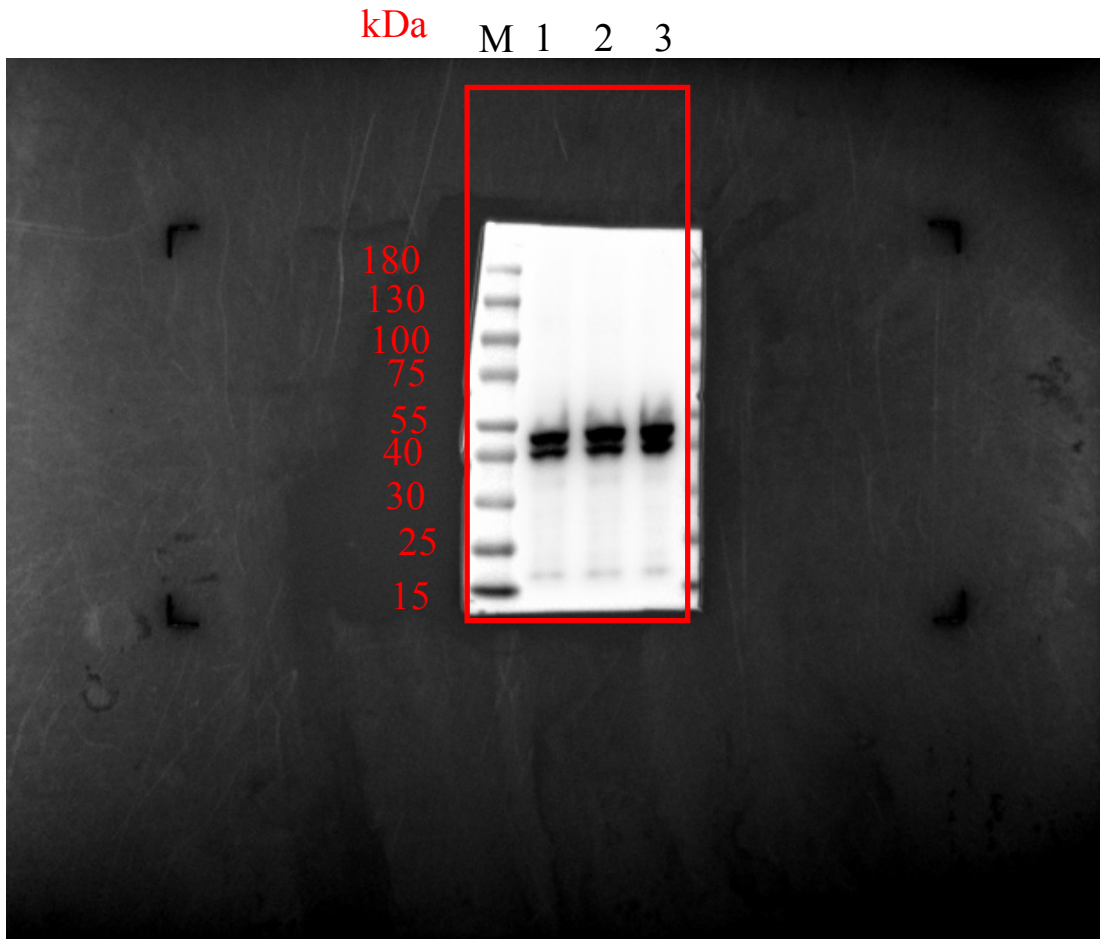

1: Input 293T HA-Vector+Myc-N

2: Input 293T HA-ZC3HAV1+Myc-N

3: Input 293T HA-ZC3HAV1+Myc-Mut-N

S5 FIGB (panel 3)

kDa M 1 2 3

Anti-HA

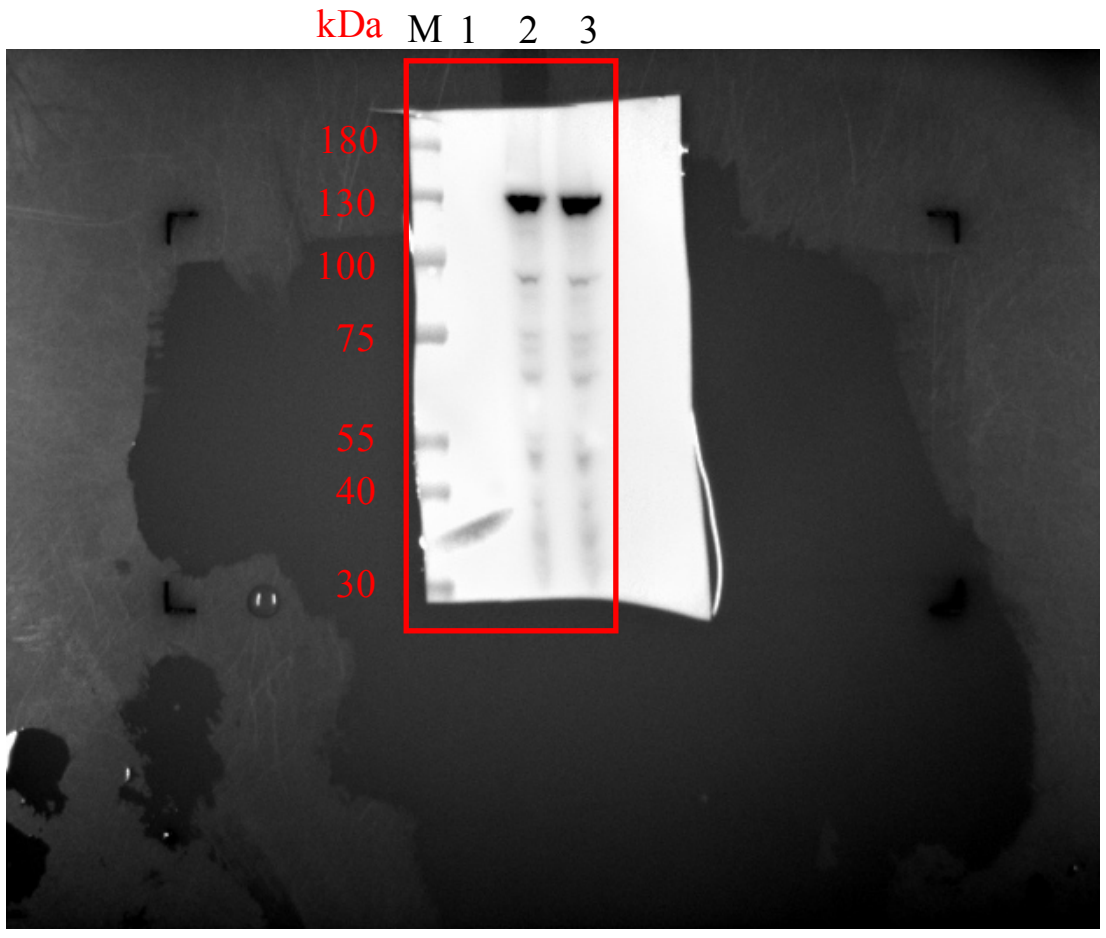

1: Ip (HA) 293T HA-Vector+Myc-N

2: Ip (HA) 293T HA-ZC3HAV1+Myc-N

3: Ip (HA) 293T HA-ZC3HAV1+Myc-Mut-N

S5 FIGB (panel 4)

Anti-Myc

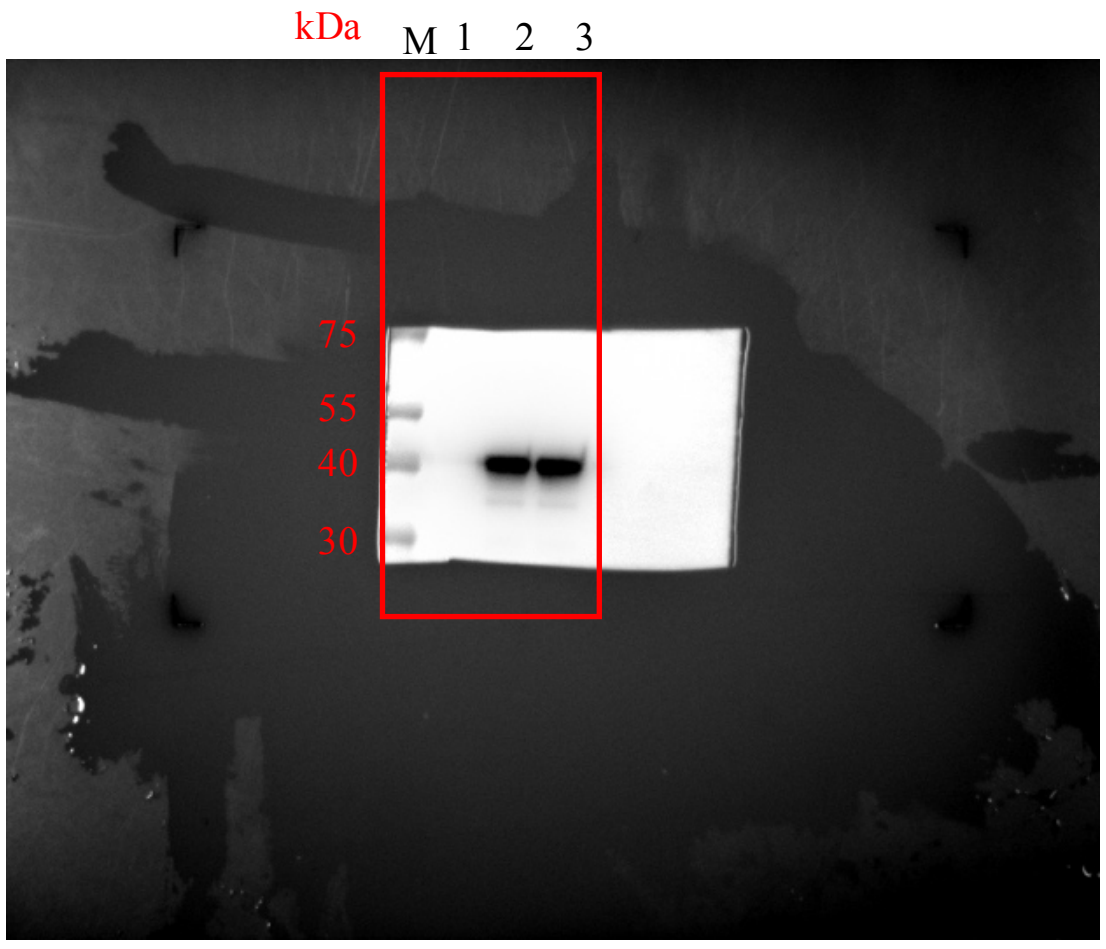

1: Ip (HA) 293T HA-Vector+Myc-N

2: Ip (HA) HA-ZC3HAV1+Myc-N

3: Ip (HA) HA-ZC3HAV1+Myc-Mut-N
