# Supplementary material for: A feline coronavirus nucleocapsid protein disrupts ZC3HAV1–viral RNA association to counteract host RNA-level restriction
Source: PLoS Pathog. 2026 Sep 18;22(9):e1014616. doi: 10.1371/journal.ppat.1014616 (PMC13600618; doi:10.1371/journal.ppat.1014616)
Supplement: Supplementary file 2 [file ppat.1014616.s008.docx]

S1 Table. Primers required for plasmid construction

| Amplicon | Primer Name | Primer Sequence (5'-3') |
| --- | --- | --- |
| N(Myc) | N(Myc)-F | CGCGGGCCCAGGCCCGAATTCGCCACCATGGCCACACAGGGACAACGCGT |
|  | N(Myc)-R | CAGCTTCTGCTCGCCGGTACCGTTCGTAACCTCATCAATCAT |
| N(Flag) | N(Flag)-F | AGGTCTATATAAGCAGAGCTCGCCACCATGGCCACACAGGGACAACGC |
|  | N(Flag)-R | TTTGTAGTCAGCCCGGGATCCGTTCGTAACCTCATCAATCAT |
| N(GST) | N(GST)-F | CCGCGTGGATCCCCGGAATTCATGGCCACACAGGGACAACGC |
|  | N(GST)-R | GTCACGATGCGGCCGCTCGAGGTTCGTAACCTCATCAATCAT |
| ZC3HAV1  (Flag) | ZC3(F)-F | CATCATTTTGGCAAAGAATTCGCCACCATGGCGGACCCGGAGGTGTGCT |
|  | ZC3(F)-R | GTAATCCATAGATCTGCTAGCACTAATCACGCAGGCTTTGTCT |
| ZC3HAV1  (HA) | ZC3(HA)-F | GGGCCCAGGCCCGAATTCGCCACCATGGCGGACCCGGAGGTGTGCTGCT |
|  | ZC3(HA)-R | ATCGTATGGGTAGCCGGTACCACTAATCACGCAGGCTTTGTCTAG |
| ZC3HAV1  (28a) | ZC3(28a)-F | GACAGCAAATGGGTCGCGGATCCATGGCGGACCCGGAGGTGTGCTGCT |
|  | ZC3(28a)-R | AGTGGTGGTGGTGGTGGTGCTCGAGACTAATCACGCAGGCTTTGTCTAGT |
| ZC3HAV1-Z1 | Z1-F | TTTGGCAAAGAATTCGCCACCATGGCGGACCCGGAGGTGTG |
|  | Z1-R | GTAATCCATAGATCTGCTAGCTTTCTTTCGGCCATGCTTGC |
| ZC3HAV1-Z2 | Z2-F | TTTGGCAAAGAATTCGCCACCATGGCGGACCCGGAGGTGTG |
|  | Z2-R | GTAATCCATAGATCTGCTAGCGTGAACTTTGTTGCAGCTTGC |
| ZC3HAV1-Z3 | Z3-F | TTTGGCAAAGAATTCGCCACCATGCACCCCAGGGGCAAAGCTCCTTGC |
|  | Z3-R | GTAATCCATAGATCTGCTAGGTGAACTTTGTTGCAGCTTGCAAG |
| ZC3HAV1-Z4 | Z4-F | TTTGGCAAAGAATTCGCCACCATGATTTGTCTTGACCATCTGTGT |
|  | Z4-R | GTAATCCATAGATCTGCTAGCGTAAGACATGAATTTTGGCCT |
| ZC3HAV1-Z5 | Z5-F | TTTGGCAAAGAATTCGCCACCATGTTCCACCTGCCTTACCGGTGG |
|  | Z5-R | GTAATCCATAGATCTGCTAGCACTAATCACGCAGGCTTTGTC |
| ZC3HAV1-Z6 | Z6-F | TTTGGCAAAGAATTCGCCACCATGTCAGAGCCGTTAACCTCGACA |
|  | Z6-R | GTAATCCATAGATCTGCTAGCACTAATCACGCAGGCTTTGTC |
| TOP1(Flag) | TOP1-F | CATCATTTTGGCAAAGAATTCGCCACCATGAGCGGGGACCATCTCCAC |
|  | TOP1-R | GTAATCCATAGATCTGCTAGCGAACTCGTAGTCCTCATCAGC |
| SEC13  (Flag) | SEC13-F | CATCATTTTGGCAAAGAATTCGCCACCATGGTATCGGTAATTAACACT |
|  | SEC13-R | GTAATCCATAGATCTGCTAGCCTGCTCGTTCTGCTGGCCCTCTGT |
| DDX18  (Flag) | DDX18-F | CATCATTTTGGCAAAGAATTCGCCACCATGTCGCATCTACCCATGAAAC |
|  | DDX18-R | GTAATCCATAGATCTGCTAGCGTGGGAGAACTGCCTGCTGTCA |
| RPL31  (Flag) | RPL31-F | CATCATTTTGGCAAAGAATTCGCCACCATGGCTCCCGCAAAGAAGGGTG |
|  | RPL31-R | GTAATCCATAGATCTGCTAGCGTTCTCATCCACATTAACAGTCT |
| EIF3M  (Flag) | EIF3M -F | CATCATTTTGGCAAAGAATTCGCCACCATGAGCGTCCCGGCCTTCATTG |
|  | EIF3M-R | GTAATCCATAGATCTGCTAGCTGTATCAGAAAGACTCAAAAGGC |
| EIF3D  (Flag) | EIF3D -F | CATCATTTTGGCAAAGAATTCGCCACCATGGCAAAGTTCATGACACCTG |
|  | EIF3D-R | GTAATCCATAGATCTGCTAGCAGTTTCTTCCTCTTCTTCCTCCT |

S1 Table. Primers required for plasmid construction (continued)

| Amplicon | Primer Name | Primer Sequence (5'-3') |
| --- | --- | --- |
| PKP1  (Flag) | PKP1-F | CATCATTTTGGCAAAGAATTCGCCACCATGAACCACTCGCCGCTCAAGA |
|  | PKP1-R | GTAATCCATAGATCTGCTAGCGAATCGGGAGGTGAAGTTCCTG |
| RPS15  (Flag) | RPS15-F | CATCATTTTGGCAAAGAATTCGCCACCATGGCGGAAGTGGAACAGAAGA |
|  | RPS15-R | GTAATCCATAGATCTGCTAGCCTTGAGGGGGATGAAGCGGGAC |
| ASPH  (Flag) | ASPH -F | CATCATTTTGGCAAAGAATTCGCCACCATGGCCCCGCGCAAGAATGCCA |
|  | ASPH-R | GTAATCCATAGATCTGCTAGCAATCGCAGGAAGGCTCTGTCTCT |
| SPATS2L(Flag) | SPATS2L -F | CATCATTTTGGCAAAGAATTCGCCACCATGGCTGAACTCAATACTCATGT |
|  | SPATS2L -R | GTAATCCATAGATCTGCTAGCGGCCACCAACGTTACAGCTGGGA |
| MOV10 (Flag) | MOV10 -F | CATCATTTTGGCAAAGAATTCGCCACCATGGGAGGAGGGGGAGGGGATTGGAC |
|  | MOV10 -R | GTAATCCATAGATCTGCTAGCGAGCTCATTCCTCCACTCTGGC |
| HNRNPF  (Flag) | HNRNPF -F | CATCATTTTGGCAAAGAATTCGCCACCATGATGCTGGGCCCCGAGGGAG |
|  | HNRNPF -R | GTAATCCATAGATCTGCTAGCGTCATATCCACCCATGCTGTTCT |
| RPL36AL(Flag) | RPL36AL -F | CATCATTTTGGCAAAGAATTCGCCACCATGGTCAATGTTCCGAAAACCCG |
|  | RPL36AL -R | GTAATCCATAGATCTGCTAGCGAACTGGATCACTTGGCCCTTTC |
| OSBPL3  (Flag) | OSBPL3 -F | CATCATTTTGGCAAAGAATTCGCCACCATGAGTGATGAGAAGAACCTTGG |
|  | OSBPL3 -R | GTAATCCATAGATCTGCTAGCCCACAAGACAGGATGGTCCAGT |
| RPL35A  (Flag) | RPL35A -F | CATCATTTTGGCAAAGAATTCGCCACCATGCCTGAGGGCGGGGCGGAGA |
|  | RPL35A -R | GTAATCCATAGATCTGCTAGCAATCCTTGAGGGGTACAGCATCA |
| RALY  (Flag) | RALY -F | CATCATTTTGGCAAAGAATTCGCCACCATGTCCTTGAAGATCCAGACAA |
|  | RALY -R | GTAATCCATAGATCTGCTAGCCTGCAAGGCCCCATCCTCCG |
| PES1  (Flag) | PES1 -F | CATCATTTTGGCAAAGAATTCGCCACCATGGGAGGCCTAGAGAAGAAGA |
|  | PES1 -R | GTAATCCATAGATCTGCTAGCCACGGGCCTGGCCTTCTTGGC |
| PRDX6  (Flag) | PRDX6 -F | CATCATTTTGGCAAAGAATTCGCCACCATGCCTGGAGGTCTCCTTCTTG |
|  | PRDX6 -R | GTAATCCATAGATCTGCTAGCCGGCTGGGGCGTGTAACGGAGG |
| FIPV-N (N1) | N1-F | TTTGGCAAAGAATTCGCCACCATGGCCACACAGGGACAACGC |
|  | N1-R | GTAATCCATAGATCTGCTAGCCACTTCAAGCTGAAACTGTGG |
| FIPV-N (N2) | N2-F | TTTGGCAAAGAATTCGCCACCATGGCCACACAGGGACAACGC |
|  | N2-R | GTAATCCATAGATCTGCTAGCTTTGGAATCACTACGTTCTCT |
| FIPV-N (N3) | N3-F | TTTGGCAAAGAATTCGCCACCATGAATGATATACCTTTGTCATTC |
|  | N3-R | GTAATCCATAGATCTGCTAGCTTTGGAATCACTACGTTCTCT |
| FIPV-N (N4) | N4-F | TTTGGCAAAGAATTCGCCACCATGAATGATATACCTTTGTCATTC |
|  | N4-R | GTAATCCATAGATCTGCTAGCAGAAGGTCGCTTGTAAGCGTCA |
| FIPV-N (N5) | N5-F | TTTGGCAAAGAATTCGCCACCATGCCTAGGGACACAACACCTAAG |
|  | N5-R | GTAATCCATAGATCTGCTAGCGTTCGTAACCTCATCAATCAT |
| Mut-N-1 | Mut-N-1F | ATGGCCACACAGGGACAACGCGTCAACTGG |
|  | Mut-N-1R | GTTGTGGGAGCGTTCATGGCACCATCAGCTGCAA |

S1 Table. Primers required for plasmid construction (continued)

| Amplicon | Primer Name | Primer Sequence (5'-3') |
| --- | --- | --- |
| Mut-N-2 | N-WT-2F | GGTGGTTCTTTGCTTTCTTAGGTAC |
|  | N-WT-2R | GTTCGTAACCTCATCAATCATCTCA |
| pLKO-ZC3HAV1 | Sh-ZC3 | TGGAAAGGACGAAACACCGGTGCACTTTACTCGAGGGATTTGCTCGAGCAAATCCCTCGAGTAAAGTGCTTTTTGAATTCTCGACCTCGAGACAA |
