# Supplementary material for: A feline coronavirus nucleocapsid protein disrupts ZC3HAV1–viral RNA association to counteract host RNA-level restriction
Source: PLoS Pathog. 2026 Sep 18;22(9):e1014616. doi: 10.1371/journal.ppat.1014616 (PMC13600618; doi:10.1371/journal.ppat.1014616)
Supplement: Supplementary file 3 [file ppat.1014616.s009.docx]

S2 Table. Primers used for RT-qPCR analysis of host gene transcription

| Amplicon | Primer Name | Primer Sequence (5'-3') |
| --- | --- | --- |
| GAPDH | GAPDH-qF | AGTATGATTCCACCCACGGCA |
|  | GAPDH-qR | GATCTCGCTCCTGGAAGATGGT |
| ZC3HAV1 | ZC3-qF | CCACCTGCTTCCGCTTCTACTC |
|  | ZC3--qR | TTCCATGTGACCTGTTCCTCCAAG |
| TOP1 | TOP1-qF | TTGTAACCATCAGAGGGCACCAC |
|  | TOP1-qR | GGCATCTGCTAGTTGTTCCTTCTTG |
| SEC13 | SEC13-qF | GAAGCCTCATAGACCAGCCATCG |
|  | SEC13-qR | ATCCTCCTCCTCCTTCCACAGC |
| DDX18 | DDX18-qF | AGCCACACAGCCTCCCAATTC |
|  | DDX18-qR | GGCACCTCAGCGTCTTCTTCC |
| RPL31 | RPL31-qF | GCTTTCCCTCTCCCACAATACCTC |
|  | RPL31-qR | TCTTCTCGCCACCCTTCTTTGC |
| EIF3M | EIF3M-qF | GATTGGAGCCGATGATGTTGAAGC |
|  | EIF3M-qR | TCACAACTACTTTCCTCTGGGTCTG |
| EIF3D | EIF3D-qF | AAGCGACCTACATCAACCACAAC |
|  | EIF3D-qR | GCGACGGAGGCAACTTCATTC |
| PKP1 | PKP1-qF | GCTATCCGCACCTACCTGAACC |
|  | PKP1-qR | AACTGGCTCATACCACTGGACATC |
| RPS15 | RPS15-qF | GGCGAGTTCTCCATCACCTACAAG |
|  | RPS15-qR | GGGATGAAGCGGGACGAGTG |
| ASPH | ASPH-qF | CCACGGGCGAGATACGGAAAG |
|  | ASPH-qR | CTTCAAGGTCAGCTTCAGCAAGTC |
| SPATS2L | SPATS2L-qF | GAAGCAAGTCTAAGCAGCATCAAGG |
|  | SPATS2L-qR | TGGCAGAATCGGTGGATGAGTTG |
| MOV10 | MOV10-qF | ACATCAGCAAACACCACAAGTCAC |
|  | MOV10-qR | GTTCCTCCATTCCGAAGCCTCAG |
| HNRNPF | HNRNPF-qF | CAGATACGGAGACGGCGAGTTC |
|  | HNRNPF-qR | AGGGTTGAGTGGAGAGAAGAAGTTG |
| RPL36AL | RPL36AL-qF | TGTCGCTCACGCACAGTTCC |
|  | RPL36AL-qR | CAGCACGATTCAAACGGCAAATTC |
| OSBPL3 | OSBPL3-qF | GGCTGAGAGGAGAGATGAGTTACAC |
|  | OSBPL3-qR | ATCGGTTTGGCTCTTGGCATATTTC |
| RPL35A | RPL35A-qF | CGCCTCTCTCTTTCCGCCATC |
|  | RPL35A-qR | GCTGTGTGCTCCCTCTGGTTC |

S2 Table. Primer information for qPCR detection of host gene transcription levels (continued)

| Amplicon | Primer Name | Primer Sequence (5'-3') |
| --- | --- | --- |
| RALY | RALY-qF | GCTGTGCTGGGAGAGAATGGG |
|  | RALY-qR | CCTTGGGTCTGTTGGGCTTGG |
| PES1 | PES1-qF | GTTGCCTCCACACCTCTCACC |
|  | PES1-qR | CCACATCTTCTTCCTCCTCCTCTTC |
| PRDX6 | PRDX6-qF | CTTCACCAGCATTCTGTTCTGTTCC |
|  | PRDX6-qR | TTCTCCACCATCAACTGACACTACC |
| HSPG2 | HSPG2-qF | CAGACCCTGGATCTGAACTGCAT |
|  | HSPG2-qR | GACACCTGATTCAGCCTTAGCAGG |
