# Supplementary material for: A feline coronavirus nucleocapsid protein disrupts ZC3HAV1–viral RNA association to counteract host RNA-level restriction
Source: PLoS Pathog. 2026 Sep 18;22(9):e1014616. doi: 10.1371/journal.ppat.1014616 (PMC13600618; doi:10.1371/journal.ppat.1014616)
Supplement: Supplementary file 4 [file ppat.1014616.s010.docx]

S3 Table. siRNA sequences targeting ZC3HAV1

| Primer Name | | Primer Sequence (5'-3') |
| --- | --- | --- |
| siZC3-1 | Sense | CAUGCAUUUGGGAAGUCAA(dT)(dT) |
|  | Antisense | UUGACUUCCCAAAUGCAUG(dT)(dT) |
| siZC3-2 | Sense | GGACGAAACCACUGAUGUA(dT)(dT) |
|  | Antisense | UACAUCAGUGGUUUCGUCC(dT)(dT) |
| siZC3-3 | Sense | GGGAGAAGGUCGACAAACA(dT)(dT) |
|  | Antisense | UGUUUGUCGACCUUCUCCC(dT)(dT) |
