# Supplementary material for: A feline coronavirus nucleocapsid protein disrupts ZC3HAV1–viral RNA association to counteract host RNA-level restriction
Source: PLoS Pathog. 2026 Sep 18;22(9):e1014616. doi: 10.1371/journal.ppat.1014616 (PMC13600618; doi:10.1371/journal.ppat.1014616)
Supplement: Supplementary file 5 [file ppat.1014616.s011.docx]

S4 Table. Primers used to establish qPCR assays for FIPV gRNA and sgRNAs.

| Amplicon | Primer Name | | Primer Sequence (5'-3') |
| --- | --- | --- | --- |
| 5' UTR(8～30) | | LP1-F | AAGTAAAGTGAGTGTAGCGTGGC |
| 5' UTR(36～59) | | LP2-F | CTCTTCTTTTACTTTAACTAGCC |
| 5' UTR(50～74) | | LP3-F | TAACTAGCCTTGTGCTAGATTTGT |
| 5' UTR(62～85) | | LP4-F | TGCTAGATTTGTCTTCGGACACC |
| 5' UTR(77～90) | | LP5-F | CGGACACCAACTCGAACTAAACG |
| 5'UTR(108～131) | | LP6-F | GTCTCTCTATGAAACCATAGAAG |
| 5'UTR (131～153) | | LP7-F | ACAAGCGTTGATTATTTCACCAG |
| 5'UTR (169～191) | | LP8-F | TAGGAACGGGGTTGAGAGAACG |
| 5'UTR (203～226) | | LP9-F | TCCGTCCCTGTTTGGTAAGTCGT |
| 5'UTR (216～241) | | LP10-F | GGTAAGTCGTCTAGTATTAGCTGCG |
| 5'UTR (247～269) | | LP11-F | CTGCCCGTCGTAGTTGGGTAGA |
| 5'UTR (274～296) | | LP12-F | TTCCGTCCTGTGATCTCCCTCG |
| S(206) | | S-sgRNA-R | GAGCAGTTGTACCACACCTCTGTAG |
| N(122) | | N-sgRNA-R | AGGGTAATGGGGTTGTAGAATGA |
| M(178) | | M-sgRNA-R | CAGACCAGCTGAAGTTCCAGTTAG |
| E(194) | | E-sgRNA--R | TAGGCATCATATGCATGGCGTGC |
| 7a(153) | | 7a-sgRNA-R | TACACGCAGACTACTGTCAGGCA |
| 7b(209) | | 7b-sgRNA-R | GTGCAATTGAAACCTTCGATACC |
| 3a(56) | | 3a-sgRNA-R | CAAGTTCGTCAAGTACAGCGTCT |
| 3c(73) | | 3c-sgRNA-R | TTTATGCCACCATACAATGTGAC |
| pp1a/pp1b (20027) | | gRNA-F | ATTGAATGAAATGGTCGTTGGAT |
| S(120) | | gRNA-R | GATAAGGTTTTCATTGCCAGCCA |
