# Supplementary material for: A feline coronavirus nucleocapsid protein disrupts ZC3HAV1–viral RNA association to counteract host RNA-level restriction
Source: PLoS Pathog. 2026 Sep 18;22(9):e1014616. doi: 10.1371/journal.ppat.1014616 (PMC13600618; doi:10.1371/journal.ppat.1014616)
Supplement: Supplementary file 6 [file ppat.1014616.s013.docx]

**S6 Table. Functional overview of 18 representative FIPV N-associated host proteins selected for validation.**

| **Protein accession** | **Protein name** | **Protein function** | **Peptide coverage** |
| --- | --- | --- | --- |
| M3VUI2 | EIF3M | Eukaryotic translation initiation factor 3 subunit M | 53 |
| M3VW18 | EIF3D | Eukaryotic translation initiation factor 3 subunit D | 38 |
| A0A2I2U3C8 | ASPH | Aspartate beta-hydroxylase | 11 |
| M3VVC3 | DDX18 | RNA helicase | 12 |
| A0A2I2V0T7 | RPL31 | Uncharacterized protein | 43 |
| A0A337SQ02 | RPS15 | Ribosomal protein S15 | 41 |
| M3WIM4 | MOV10 | Mov10 RISC complex RNA helicase | 5 |
| A0A2I2U6Y3 | OSBPL3 | Oxysterol binding protein like 3 Gene | 4 |
| M3X2R1 | ZC3HAV1 | Poly [ADP-ribose] polymerase | 2 |
| A0A337SK14 | RPL35A | Ribosomal protein L35a | 19 |
| A0A2I2UL25 | PRDX6 | Peroxiredoxin 6 | 4 |
| M3WFV3 | RPL36 | 60S ribosomal protein L36 | 29 |
| M3XBR2 | HNRNPF | Heterogeneous nuclear ribonucleoprotein F | 55 |
| M3W2I5 | PKP1 | Plakophilin 1 | 5 |
| A0A2I2V4T4 | SPATS2L | Spermatogenesis associated serine rich 2 like | 2 |
| A0A337S9A5 | SEC13 | SEC13 homolog, nuclear pore and COPII component | 26 |
| M3WAN6 | TOP1 | DNA topoisomerase I | 12 |
| A0A5F5XHL0 | RALY | RALY heteroous nuclear ribonucleoprotein | 13 |
